# Integrative clustering of multi-level ‘omic data based on non-negative matrix factorization algorithm

**DOI:** 10.1371/journal.pone.0176278

**Published:** 2017-05-01

**Authors:** Prabhakar Chalise, Brooke L. Fridley

**Affiliations:** 1 Department of Biostatistics, University of Kansas Medical Center, Kansas City, Kansas, United States of America; 2 Department of Biostatistics and Bioinformatics, Moffitt Cancer Center, Tampa, Florida, United States of America; National Institute of Environmental Health Sciences, UNITED STATES

## Abstract

Integrative analyses of high-throughput ‘omic data, such as DNA methylation, DNA copy number alteration, mRNA and protein expression levels, have created unprecedented opportunities to understand the molecular basis of human disease. In particular, integrative analyses have been the cornerstone in the study of cancer to determine molecular subtypes within a given cancer. As malignant tumors with similar morphological characteristics have been shown to exhibit entirely different molecular profiles, there has been significant interest in using multiple ‘omic data for the identification of novel molecular subtypes of disease, which could impact treatment decisions. Therefore, we have developed *intNMF*, an integrative approach for disease subtype classification based on non-negative matrix factorization. The proposed approach carries out integrative clustering of multiple high dimensional molecular data in a single comprehensive analysis utilizing the information across multiple biological levels assessed on the same individual. As *intNMF* does not assume any distributional form for the data, it has obvious advantages over other model based clustering methods which require specific distributional assumptions. Application of *intNMF* is illustrated using both simulated and real data from The Cancer Genome Atlas (TCGA).

## Introduction

Identification of molecular subtypes of disease has received a great deal of attention using disparate types of high throughput ‘omic data sets. Due to the advent of high throughput microarray and next-generation sequencing (NGS) technologies, vast amounts of multi-level molecular data have been accumulated lending to the study of “systems biology”. The underlying principal of integrative analysis is that the biological mechanisms of disease are attributed to the complex relationship and interplay within and between several levels of biological processes [1]. Therefore, collective understanding of relationships between the various biological levels (e.g., genome, transcriptome, epigenome, proteome), in addition to variations within each biological level, are critical to the understanding of disease etiology, treatment, and progression.

One such integrative analyses approach is classifying the subjects into various subgroups using clustering techniques. There exist countless different clustering methodologies using a single data type at a time and only a few methods that use multiple data sets in a single comprehensive step [2]. The purpose of such methods is to group the objects across a discrete set of classes (i.e. clusters) such that the objects within the same class are more similar to one another as compared to objects in different classes. With the application of clustering to a data set, one can either cluster the features (i.e., genes) or the samples (i.e., tumors from patients). In this article we focus on clustering the samples with the goal of identifying molecular subtypes of disease.

Conventional approaches for clustering samples based on multiple ‘omics datasets have involved the manual integration of results obtained from individual clustering of each of such ‘omics data types. Such methods require great deal of understanding of all the data types and the biology associated with them in order to fully utilize the available information. Although such approaches will be able to capture a strong effect across multiple assays, it may miss possible weak but consistent relationship across the multiple data types that may be equally informative.

Depending on the technology and unit of measurement used to assess the biological process, the data can follow a wide range of distributions. Therefore it is very difficult to model the statistical distributions of all such datasets in a single integrative analysis. One of the most commonly used integrative clustering method, iCluster, models the tumor subtypes as an unobserved latent variable assuming that the data follow Gaussian probability distribution [3]. The extended version iCluster+ has flexibility of incorporating dichotomous data that follows Binomial and count data that follows Poisson distribution [4]. Another example of integrative clustering method uses a Gaussian mixture model [5]. In contrast to these methods based on latent variables approach, Kirk et al. [6] have proposed Bayesian mixture modeling framework in which each dataset is modelled using Dirichlet-multinomial allocation mixture model. However, if the model assumptions are not satisfied, the model based methods can provide misleading results. To this end, non-negative matrix factorization (NMF) algorithm first proposed by Lee & Seung [7] and several variants of it [8–10], have been proposed for clustering a single high-dimensional data. Zhang et al.[11] extended the NMF algorithm for multiple data to identify the subsets of multidimensional genomic data (blocks of data) that have correlated profiles, termed as multidimensional module, across several types of data. However, the method cannot classify the subjects into disjoint set of clusters in order to discover the disease subtypes.

In this article, we propose integrative clustering method based on NMF, *intNMF*, for classifying subjects into disjoint set of clusters using multiple sources of molecular data. The proposed method does not assume any distributional form of the data. The method is illustrated and compared to iCluster using both simulated data and data collected within the TCGA network for Breast and Glioblastoma cancers.

## Materials & methods

### Non-negative matrix factorization

NMF approach has been applied in several fields after it was formally proposed in 1994 by Paatero & Tapper [12], with the algorithm outlined in 1999 by Lee & Seung [7]. Brunet et al. [8] utilized the algorithm as it is in cancer subtype discovery, while others have added additional regularization constraints to create sparse solutions [9, 10]. Suppose $X_{n\times p}\in R^{n\times p}$ is a matrix, having all non-negative entries, with *n* subjects and *p* measured features. NMF factorizes the matrix ***X***_*n×p*_ into two non-negative matrices such that. ***X***_*n×p*_ ≈ ***W***_*n×k*_***H***_*k×p*_, where *k* represents the pre-set number of groups or clusters, ***W***_*n×k*_ is a matrix of basis vectors, and ***H***_*k×p*_ is the matrix of coefficient vectors. Each column of ***X*** can be written as ***X***[,*col*] ≈ ***WH***[,*col*], where ***X****[*,*col]* and ***H****[*,*col]* are the corresponding columns in ***X*** and ***H*** respectively. Each data vector ***X***[,*col*] is approximated by a linear combination of the columns of ***W*** weighted by the components of ***H****[*,*col]*. Therefore ***W*** is regarded as a matrix of basis vectors which is optimized for the linear approximation of the data in ***X*** and can be used to classify the subjects into groups or clusters.

### Integrative NMF (*intNMF*)

We propose the following extension of NMF to allow for the clustering of subjects using multiple biological sources of data (e.g., mRNA expression, DNA methylation, protein expression). Let ***X***^***i***^, *i = 1*, *2*,*…*, *m* be matrices representing *m* data types profiled on *n* samples with *p*_*i*,_ *i = 1*, *2*,*…*, *m* features (i.e., ***X***^1^ would be a *n* × *p*_1_ matrix). Integrative clustering with NMF is carried out by estimating common basis matrix ***W*** and data specific coefficient matrices ***H***^***i***^ such that

$$X_{n\times p_{i}}^{i}\approx W_{n\times k}H_{k\times p_{i}}^{i},\;i=1,2,\ldots m,$$ (1)

where all entries of ***W*** and ***H***^***i***^ are non-negative. The objective function is then defined as the weighted Frobenius norm

$$Q={\text{min}}_{W,H}\sum_{i=1}^{m}\theta^{i}||X^{i}-WH^{i}|{|}_{2}.$$ (2)

*θ*^*i*^ > 0 is the user specified weight for *i*^th^ data. For example, the weights can be calculated as the maximum of the mean sums of squares among all data divided by the mean sums of squares of each data $\left(\theta^{i}=\frac{\;Max\;\left\{mean\{||X^{i}|{|}_{2}\},\;i=1,\ldots ,m\right\}}{mean\{||X^{i}|{|}_{2}\}},\;i=1,\ldots ,m\right)$. The function *Q* is convex with respect to ***W***; however, the function is not convex when ***W*** and all ***H***^***i***^ are considered together. Therefore, there is no unique global minimum of the NMF problem [12, 13]. However, a local optimum can be achieved by minimizing the objective function *Q* using numerical optimization methods.

As NMF imposes the non-negativity constraint, the linear combination has only the additive effect if the effect is present (because the effect is positive if it is present otherwise it is zero), and is compatible with the intuitive notion of combining parts to form the whole. Many studies have shown that a good local minima can provide desirable properties such as pattern recognition, grouping of the variables in the data etc [7–11]. The strategy to finding the “best” local minima is to determine numerous local minimums using several initializations of ***W*** and ***H***^*i*^ and then choosing the one for which objective function *Q* with the smallest value.

The higher order generalized singular value decomposition (HO GSVD) [14] and its variants [15, 16] have also been proposed for integrative matrix factorization. These methods are the extensions of singular value decomposition (SVD). SVD factorizes the matrix ***X*** into ***UΣV***^***T***^ where ***U*** and ***V*** are orthogonal matrices having both positive and negative elements. ***Σ*** is diagonal matrix with non-negative numbers on the diagonal. NMF approximately factorizes the non-negative matrix ***X*** into non-negative matrices ***W*** and ***H***. The main difference between the SVD and NMF is that the matrices ***U*** and ***V*** contains both positive and negative elements while matrices ***W*** and ***H*** contain only the non-negative elements. Lee & Seung (1999)[7], in their seminal work, have extensively carried out the comparison between SVD based method and NMF in the application of pattern recognition and have concluded that NMF is more efficient than SVD for pattern recognition studies. This is because the negative values in the factor loadings of ***U*** or ***V*** can result in contradicting physical realities because some of the effects for some important features cancel each other. The extended higher order generalized SVD (HO GSVD) factorizes the matrices ***X***^***i***^ into ***U***^***i***^***Σ***^***i***^***V***^***T***^, for *i = 1*,*…*,*m* where ***V*** is common across the data and contains the pattern-structure shared across the several data sets. intNMF factorizes the matrices ***X***^***i***^ into ***WH***^***i***^ for *i = 1*,*…*,*m* where ***W*** is common across the data sets that contains the cluster structure across the multiple data sets. Since both of these methods are extensions of the method designed for single data matrix, the similar differences in the performance of pattern recognition exist in HO SVD and intNMF.

### NMF algorithms

The available algorithms to find a solution to the system of equations defined by *Q* can be divided into three general classes: (i) multiplicative update algorithms; (ii) gradient descent algorithms; and (iii) alternating least square algorithms [13]. Lee & Seung [7] proposed an multiplicative update algorithm using mean squared error as objective function and used properties of gradient and continual descent (continual non-increase) to show that the algorithm converges to a local minimum. This claim has been questioned by a few papers with an argument that the continual descent property does not preclude descent to a saddle point instead of local minima [17, 18]. A few papers have proposed sparse NMF using multiplicative update rule [9, 19, 20]. In contrast, gradient descent algorithms [20, 21] update the elements by moving in the direction of negative gradient at a speed depending on the step size. Without proper choice of the step size, little can be said about the convergence of the gradient descent method as the convergence properties have yet to be determined [13].

For fitting intNMF we utilize the non-negative Alternating Least Square (ALS) algorithm [12], where the algorithm carries out the estimation of the matrices ***W*** and ***H*** alternatively using least squares. The basic idea lies in the fact that the objective function becomes convex in ***W*** given ***H*** and vice versa. The ALS approach is also called the “block coordinate descent” method in bound constraint optimization [22]. Unlike multiplicative algorithm where one must initialize both ***W*** and ***H***, in ALS only ***W*** has to be initialized. Moreover, in our implementation of the algorithm, no matter how many datasets are being used for the integrative analyses, only one initialization of ***W*** is required; whereas for the multiplicative algorithm both ***W*** and ***H***^*i*^,*i = 1*,*…m* matrices have to be initialized [11]. Another limitation of the multiplicative algorithm is that once the elements in ***W*** or ***H*** become 0, it must remain 0 in the successive iterative steps resulting in a “locking effect” [13, 23]. This is not an issue with ALS as the iterative procedure allows escaping from a poor path / solutions.

To ensure the non-negativity condition on ***W*** and ***H***^*i*^,*i = 1*,*…m* matrices, non-negativity constrained least square algorithm is implemented. The non-negativity constrained alternating least square (NNALS) algorithm was first proposed by Lawson & Hanson [24], with the convergence properties of NNALS having been described in detail [18, 22, 24, 25]. Solving the non-negatively constrained least squares is computationally expensive compared to un-constrained least squares. In order to overcome with this computation time, faster versions of NNALS have been proposed [26, 27]. The algorithm proposed by Van Benthem & Keenan [27] has been utilized in intNMF in order to solve for ***W*** and ***H***^*i*^ *i = 1*,*2*,*…m*, as outlined below. Derivation of the algorithm is provided within the S1 File.

**Algorithm for fitting *intNMF***

1. Initialize ***W*** randomly from uniform distribution (U[0,1]) and/or using non-negative double singular value decomposition (NNDSVD) method proposed by Boutsidis & Gallopoulos [28].
2. Solve for each ***H***^*i*^ *i = 1*,*2*,*…m* individually using NNALS and the current value for ***W***.
  $$\underset{\;}{Q_{H^{i}}=}{\text{argmin}}_{H^{i}}\;||X^{i}-WH^{i}|{|}_{2}\;i=1,2,\ldots ,m\;\text{such that }H_{k\times p_{i}}^{i}\geq 0$$ (3)
3. Solve for ***W*** using ***X***^*i*^ and current values for ***H***^*i*^ *i = 1*,*2*,*…m* using NNALS.
  $${\text{Q}}_{W}={\text{argmin}}_{W}\sum_{i=1}^{m}\theta^{i}||X^{i}-WH^{i}|{|}_{2}\;\text{such that }W_{n\times k}\geq 0$$ (4)
4. Repeat Step 2 and 3 until convergence.

Using the solution for ***W***, the cluster membership for each sample is then determined by the highest entry in each column (i.e., sample *j* is assigned in cluster *c* if ***W***[j, c] is the largest element in ***W***[j,] where *j* represents rows, *j* = *1*,*…*,*n*, and *c* represents columns in *W*, *c = 1*,*…*,*k*).

### Initialization and stopping criteria

NMF algorithms are sensitive to initialization of the matrices ***W*** and ***H***^*i*^, *i = 1*,*2*,*…m*. In our implementation of intNMF only ***W*** needs to be initialized. Many NMF algorithms utilize simple random initialization, while a few initialization methods are based on singular value decomposition (SVD) [23]. One such SVD based initialization method is non negative double singular value decomposition (NNDSVD) [28]. The algorithm contains no random numbers and is based on two SVD processes, one approximating the data matrix and the other approximating positive sections of the resulting partial SVD factors. In our algorithm we initialize several ***W*** matrices (one of them using NNDSVD and remaining using uniform distribution), one for each run of the algorithm, so that we can choose the factorization that results in the minimum value of the objective function out of those runs.

The stopping criterion in the algorithm is based on the stability in connectivity matrix [8, 29]. For each run of the algorithm, a *n×n* connectivity matrix ***C*** with all the entries either 0 or 1 is defined based on the sample assignment to the clusters. If two samples *i* and *j* belong to the same cluster then the corresponding entry of the connectivity matrix is 1 (*c*_*ij*_ = *1*) otherwise it is 0 (*c*_*ij*_ = *0*). The algorithm stops when ***C*** does not change for a pre-set number of consecutive iterations e.g. 50 iterations. Stopping criteria can also be defined based on the relative change in the reconstruction error. For each iteration, the sum of the difference between the original data and the reconstructed data $\left(\sum_{i=1}^{m}\left|X^{i}-{\left(WH^{i}\right)}_{jth\;iter}\right|\right)$ is calculated and compared with the similar value computed in the previous (*(j-1)*^*th*^) iteration. When the change in such reconstruction error falls below user specified threshold (e.g. 10^−4^), the algorithm stops. Our algorithm generates the reconstruction errors which can be used to create a plot against iterative steps in order to view the convergence trajectory but utilizes stability in ***C*** as a stopping rule.

### Estimation of optimum number of clusters (k)

The most important parameter to estimate in any clustering method is the optimum number of clusters *k* for the data, where *k* needs to be small enough to reduce noise but large enough to retain important information. A couple of methods have utilized consensus matrix defined by Monti et al. [29] to determine *k*. Consensus matrix, $\bar{C}$, is defined as the average of the connectivity matrices constructed over many iterative steps until convergence. The entries of the consensus matrix that ranges from 0 to 1, reflects the probability of clustering the two samples *i* and *j* together. Brunet et al. (2004) [8] proposed cophenetic correlation coefficient and Kim and Park (2007) [10] proposed dispersion coefficient based on consensus matrix for each pre-assigned *k*. The value of *k* that results in maximum of the coefficient is chosen as optimum.

Another approach that can be utilized based on dissimilarity measure derived from consensus matrix is the silhouette width [30]. The entries in the consensus matrix ($\bar{C}$) can be considered similar to Gower’s similarity coefficient [31] with similarity defined as the proportion of iterative runs the two samples *i* and *j* are grouped together in the same cluster. $1-\bar{C}$ can then be used as a new distance matrix in place of usual measures such as Euclidean distance [29]. Average silhouette width (*s*) is computed using $1-\bar{C}$ for each value of *k* and the value of *k* corresponding to maximum *s* is the optimum.

The method proposed by Hutchins et al (2008) [32] utilizes the variation of the residual sums of squares (RSS) between the original data ***X*** and the estimated data $\hat{X}(\approx \hat{W}\hat{H})$. RSS is calculated for each choice of *k* and plotted against *k*. The value of *k* at which the plot of RSS shows an inflection point is chosen as optimum.

Frigyesi et al. (2008) [33] indicated that the cophenetic correlation based on the consensus matrix might over fit the data. In order to minimize this possible issue we propose resampling based cross validation technic in estimating optimum number of clusters *k*. A few resampling based methods for finding optimum *k* and assessment of predictability of the clusters can be found in Dudoit and Fridlyand (2002) [34], Tibshirani and Walther (2005) [35], Kapp and Tibshirani (2007) [36] and Shen et al (2012)[37]. The idea is to partition the data into training ($X_{n_{1}\times p_{i}}^{i}$ for *i = 1*,*…*,*m*) and testing ($X_{n_{2}\times p_{i}}^{i}$ for *i = 1*,*…*,*m*) sets repeatedly. At each repetition, intNMF algorithm is applied to the training data $X_{n_{1}\times p_{i}}^{i}$ in order to estimate coefficient matrices $H_{k\times p_{i}\left(train\right)}^{i}$ *i = 1*, *2*,*…*,*m*. The coefficient matrices are then used to estimate the common basis matrix ($W_{n_{2}\times k}$) using the test data and solving the following optimization problem,

$${\text{Q}}_{W_{n_{2}\times k}}={\text{argmin}}_{W_{n_{2}\times k}}\sum_{i=1}^{m}\theta^{i}||X_{n_{2}\times p_{i}}^{i}-W_{n_{2}\times k}{\left(H^{i}\right)}_{p_{i\times k\left(train\right)}}^{T}|{|}_{2}\;\text{such that }W_{n_{2}\times k}\geq 0$$ (5)

Cluster memberships of the samples in the test data are predicted (“*predicted*”) using the $W_{n_{2}\times k}$ matrix as mentioned before. In parallel, intNMF algorithm is used in the test data $X_{n_{2}\times p_{i}}^{i}$ independently to compute the clustering assignments (“*observed*”) of the samples in test data. Under the true model, there should be a good consensus between the *predicted* and the *observed* clustering assignments as measured by adjusted rand index [38]. The process is repeated several times and average of the adjusted rand indices are computed which we call as “*Cluster Prediction Index*”. The value of *k* that results in maximum value of *Cluster Prediction Index (CPI)* is chosen as optimum number of clusters for the data.

### Simulation study

An R package *InterSIM* [39] was used to generate three related datasets involving DNA methylation, mRNA gene expression and protein expression. The simulation method is based on the real ovarian cancer datasets from the Cancer Genome Atlas (TCGA) [40]. The datasets are generated for a set of samples with realistic biological correlation between and within the dataset. Using CpG and protein to gene annotation information 367 CpGs and 160 protein map to 131 common genes. The annotation for methylation of CpG sites to genes was provided by Illumina and the protein to gene annotation was obtained from MD Anderson Cancer Center. Based on these 3 data types measured on 384 common subjects with the common mapped features, the intra- and inter- relationship between the features are estimated for use in the simulation of realistic data sets. Five different scenarios of true number of clusters, *k* = 2:6, were simulated setting 25% of the genomic features differentially expressed across the clusters for varying effect sizes of 0, to 4 in the increment of 0.5.

In order to make the input data fit non-negativity constraint of intNMF, the values of the data were shifted to positive direction by adding absolute value of the smallest negative number. Further, each data was rescaled by dividing by maximum value of the data to make the magnitudes comparable (between 0 and 1) across the several datasets. We assess five methods of finding optimum number of clusters: three of them using consensus matrix (Silhouette width, Cophenetic correlation and Dispersion), fourth using residual sums of squares (*RSS*) and fifth (*CPI*) based on cross validation. For the comparison equal weights were provided for each data (i.e. *θ*^*i*^ = 1 for all *i*). Optimum number of cluster was searched over the range of *k* = 2:8. intNMF was applied to the data generated for each scenario followed by computation of five parameters of estimating optimum *k*. The algorithm was run for 30 initializations of ***W***.

### TCGA breast and glioma studies

We illustrate the use of proposed intNMF with two problems in cancer subtype discovery. The multisource datasets for both examples are from The Cancer Genome Atlas (TCGA) studies on breast cancer and glioblastoma. The purpose of these two examples is to show how the results of proposed algorithm compare with the previously published results using two different approaches of integrative clustering. The first example comes from the TCGA network study [41] which utilized cluster-of-clusters approach in order to find out the breast cancer subtype and the second example is from the glioblastoma study by Shen et.al [37] that utilized iCluster method for subtype discovery.

#### Breast cancer

The dataset involves mRNA gene expression (17,814 genes), microRNA (1046 genes), Reverse phase protein array (RPPA, 171 proteins), DNA methylation (574 probes) and DNA Copy Number (20,630) available on 348 common tumor samples. The data set is publicly available at TCGA data portal https://tcga-data.nci.nih.gov/docs/publications/brca_2012/. The clinical data is available at https://gdc.cancer.gov/. Previous studies have found distinct clusters of tumors ranging from 2 to 10 using various characteristics of the genomic assays [42, 43]. TCGA network carried out integrative clustering of the five multisource datasets using cluster-of-clusters (C-of-C) approach [41]. Individual clustering identified 12 clusters using mRNA gene expression data, 7 clusters using microRNA, 5 clusters using DNA methylation, 5 clusters using DNA copy number and 7 clusters using protein data. Four distinct clusters were concluded using consensus clustering (cluster-of-clusters) on these individual platform specific clusters. These clusters correspond closely with the 4 well known intrinsic molecular subtypes: Basal-like, HER2-enriched, Luminal A and Luminal B.

#### Glioblastoma

The original data, both molecular and clinical, can be found at TCGA data portal https://gdc.cancer.gov/ but the preprocessed and sub-setted data are available in R package iCluster and described in Shen et al. [37]. The data involves DNA copy number variation (1599 genes), DNA methylation (1515 CpGs) and gene expression (1740 genes) measured on 55 common subjects across the three data types. Using gene expression data, Verhaak et al. [44] identified four distinct subtypes of samples: Classical, Messenchymal, Neural and Proneural. In addition, previous integrative analysis using iCluster has found 3 clusters based on these three datasets [37]. It should be noted that both the analysis using iCluster by Shen et al [37] and our application of intNMF do not include mutation status for genes such as IDH1 and TP53, whereas, such information was incorporated manually into the cluster characterization by Verhaak et al.

## Results

### Simulation study

**Fig 1:**
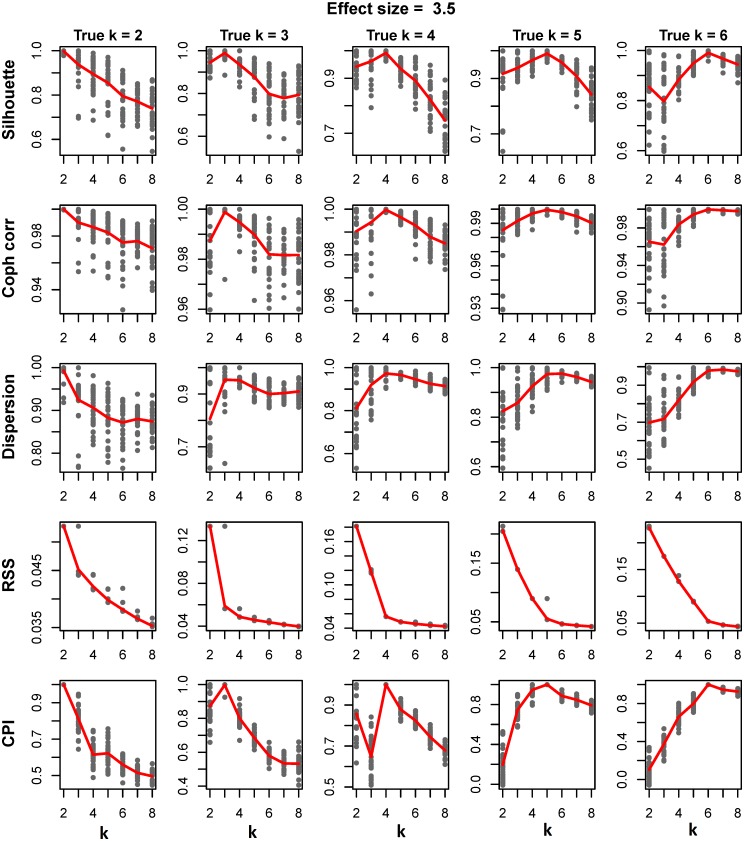
Finding optimum number of clusters. The plots represent the comparison of five different methods of finding optimum number of clusters on the dataset generated using moderate effect size of 3.5. First row represents silhouette width over k = 2:8 for each of five different scenarios of true clusters 2, 3, 4, 5 and 6 over 30 runs of simulation. The average value of the silhouette widths over 30 runs are overlaid on the plots as a line. Cophenetic correlation, Dispersion, Residual Sums of Squares and Cluster Prediction Index are shown on second, third, fourth and fifth rows respectively.

In the simulation study to look at the abilities of the various methods to determine the optimal number of clusters (*k)*, analyses were run on datasets in which the number of “true” clusters was varied from 2 to 6. Fig 1 represents the plot of the five measurements for determining *k* against the search range of *k* where the effect size was set to 3.5 (see S2 File for results for other effects sizes). The points on the plot represent the values for each of the 30 runs of the algorithm at each *k*. The average values of the parameters over the 30 runs are computed and overlaid on the plots as a line.

*Cluster Prediction Index (CPI)*, *silhouette* width and *cophenetic correlation* clearly peak at true number of cluster. In contrast, the optimal number of clusters is hard to distinguish in the plots for the *dispersion* measure. Similarly, although RSS shows the point of inflection at the true number of clusters in general, in some settings it was difficult to discern the point of inflection for estimate of *k*. Comparison of the methods over five different strengths of effect sizes for true number of cluster *k = 4* is given in S1 Fig. With the exception of the *dispersion* measure, the signal to noise ratio is maximum at true number of clusters for the various methods, with best precision with *CPI* (S2 and S3 Figs).

**Fig 2:**
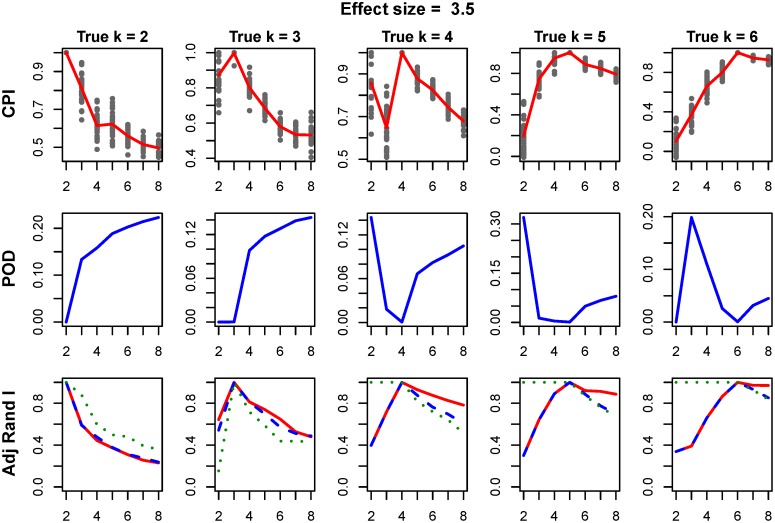
Comparison of intNMF and iCluster over varying k. First row represents the cluster prediction index, second row represents the plot of proportion of deviance (POD) given by iCluster method and third row represents adjusted rand index between (i) true and intNMF-clusters (red), (ii) true and iCluster-clusters (blue) and (iii) intNMF-clusters and iCluster-clusters (green). The POD is expected to result in minimum at true number of clusters. In other plots, maximum is expected at true number of clusters.

Next, we compared the performance of intNMF clustering to iCluster[3] (tuning parameter λ = 0.01), with results presented in Fig 2. First and second rows of Fig 2 represent the plot of *CPI* and proportion of deviance (POD), measure given by iCluster method, against search range of *k*. The third row represents the plot of adjusted rand index between true cluster membership and the clustering assignment. Both intNMF and iCluster result in optimum results (maximum value of *CPI* for intNMF and minimum value of *POD* for iCluster) at true number of simulated clusters.

Performances of the methods were further assessed with respect to cluster *purity* and *entropy*. *Purity* is defined as the proportion of samples assigned to a class that truly belongs to that class and *entropy* measures the amount of possible misclassification of the objects. Clustering performance is best when the value for *purity* is large and the value for *entropy* is small. Both methods show highest value of *purity* and smallest value of *entropy* at true number of clusters, S4 Fig. Provided at least a moderate effect size, both clustering methods determine the correct number of clusters, S5 Fig.

### TCGA data analyses

#### Breast cancer

**Fig 3:**
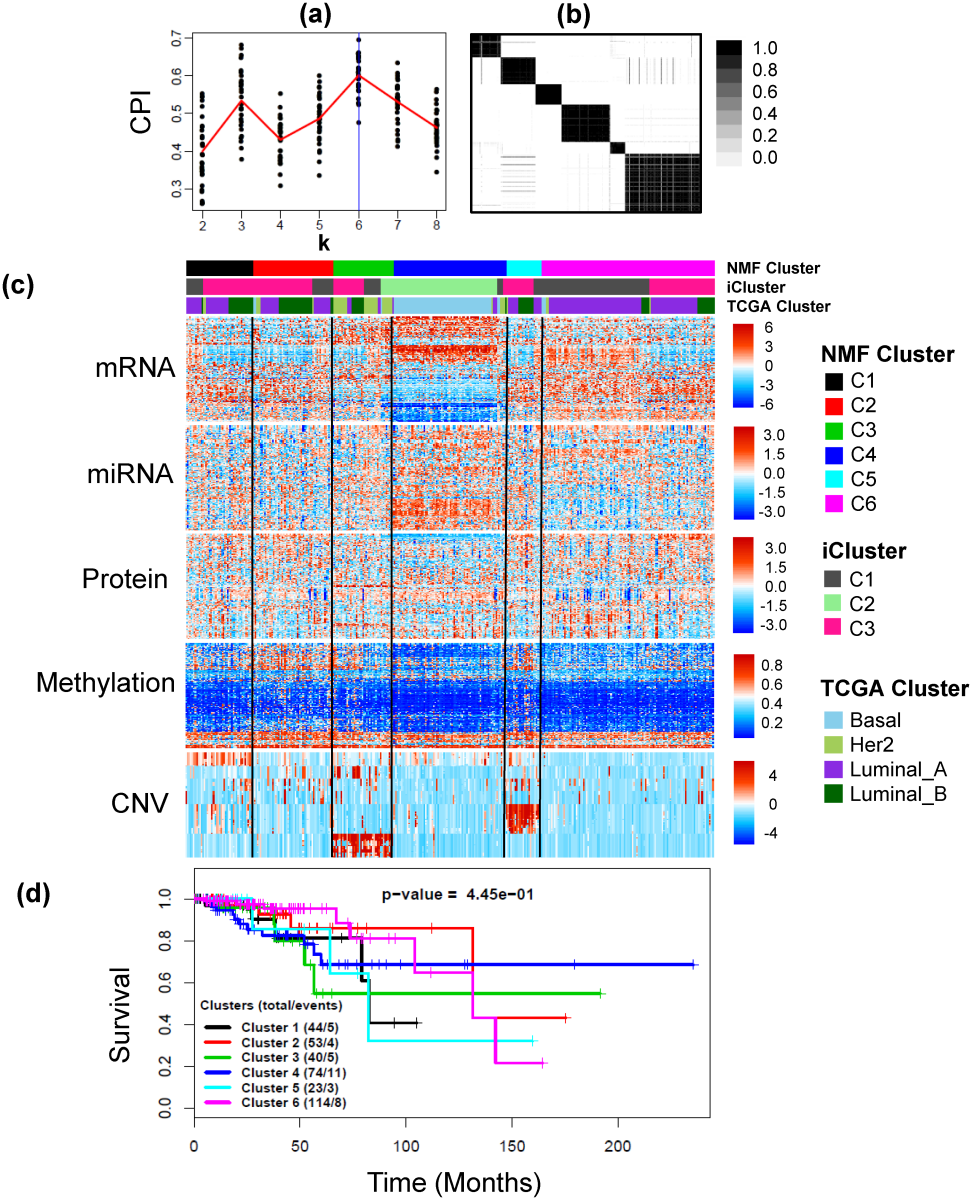
Example 1: Breast cancer data. (a) Plot of *CPI* over the search range of number of clusters from 2 to 8 for 30 runs of intNMF algorithm at each *k*. The red line represents the mean values of *CPI* at each *k*. (b) The cluster pattern as shown by the consensus matrix. (c) Heatmap of five types of data, mRNA, miRNA (log transformed and scaled), Protein, Methylation and CNV with clustering assignment from intNMF and TCGA subtypes overlaid on top with legends on the side. (d) Kaplan Meier Survival curves with p-value from log-rank test.

In order to minimize noise and optimize computational cost, the dimensionality of the mRNA, miRNA and CNV data were reduced prior to applying intNMF; mRNAs having standard deviation of at least 1.5, miRNAs having less than 50% zeros and CNVs having standard deviations of at least 0.9 were selected. The final data for integrative clustering had 645 mRNAs, 574 available methylation probes, 423 miRNAs, 409 CNVs and 171 available proteins on 348 common samples. The weights were calculated as mentioned in Methods section. intNMF algorithm resulted in 6 distinct clusters as displayed in Fig 3(a) and 3(b). Table 1 represents the cross-tabulation match between the intNMF clusters with TCGA clusters and iClusters. A moderate, but significant, overlap between the TCGA clusters and intNMF clusters was found (p-value < 2.2×10^−16^, Chi-square test). intNMF-C3 includes most of HER2-enriched tumors, while intNMF-C4 includes most of Basal-like tumors. intNMF-C1 and intNMF-C6 are enriched with Luminal A tumors, with intNMF-C2 and intNMF-C5 are comprised of both Luminal A and Luminal B tumors. TCGA network [41] found that basal like tumor had the most distinct multiplatform signature which closely agreed with results from intNMF (Fig 3(c)). iCluster-C2 is made up of Basal subtype (S1 Table) and overlaps with intNMF-C4. iCluster-C1 overlaps more with intNMF-C6 while iCluster-C3 overlaps more with intNMF clusters C1, C2, C3 and C5.

**Table 1:** Cross tabulation of intNMF subtypes with TCGA subtypes and iCluster subtypes using multiplatform Breast cancer data. The summary table, followed by cross tabulation tables, represents the receptor status for estrogen (ER), progesterone (PR) and human epidermal growth factor 2 (HER2) presented as percentage of their presence in each of the six intNMF clusters; and somatic mutations in four genes *TP53*, *PIK3CA*, *GATA3* and *MAP3K1*.

	TCGA Subtypes	intNMF						Total
		C1	C2	C3	C4	C5	C6	
**Cluster of Clusters**	**(1) HER2**	2	4	25	4	1	3	**39**
	**(2) Basal**	0	2	1	66	0	3	**72**
	**(3) Luminal A**	25	23	6	3	12	92	**161**
	**(4) Luminal B**	17	24	8	1	10	16	**76**
	**Total**	**44**	**53**	**40**	**74**	**23**	**114**	**348**
**iCluster**	**C1**	11	14	11	4	5	71	**116**
	**C2**	0	0	9	68	0	0	**77**
	**C3**	33	39	20	2	18	43	**155**
	**Total**	**44**	**53**	**40**	**74**	**23**	**114**	**348**
	**ER+ (%)**	95.5	94.3	60.0	13.5	95.7	97.4	
	**PR+ (%)**	86.4	71.7	42.5	5.4	82.6	86.8	
	**HER2+ (%)**	4.5	9.4	95.0	1.4	4.3	6.1	
	**TP53 (%)**	31.8	32.1	57.5	85.1	26.1	13.2	
	**PIK3CA (%)**	29.5	33.9	27.5	8.1	21.7	55.3	
	**GATA3 (%)**	6.8	16.9	10.0	1.4	8.7	17.5	
	**MAP3K1 (%)**	29.5	5.7	0.0	0.0	13.0	15.8	

Analysis of the long-term survival for this study data is limited because of the short follow-up time (median 1.9 years) and low number of events (36 events out of 348). Because of this, the TCGA network study [41] did not present survival analysis. Clear differences in the survival trajectories can be seen in Fig 3(d), however the differences in the survival probabilities across the six identified clusters were not up to statistical significance (p-value = 0.445, log-rank test). Also, the survival difference across the three subtypes as identified by iCluster is not statistically significant too (S6 Fig). One reason for statistical non-significance is the small number of events. In time-to-event analysis, power of statistical test depends more on the number of events than on total sample size and in this example although the sample size is decent (348) the number of events is low (36).

Somatic mutations in genes *TP53*, *PIK3CA*, *GATA3* and *MAP3K1* which were highlighted by TCGA studies [41] as subtype-associated mutations, have been presented in Table 1 as percentage of their presence in each of the six integrative clusters. The results are consistent with TCGA study findings. For example, intNMF cluster C4 is characterized by *TP53* mutations (85.1%) that includes most of Basal-like tumors (84% mutation, TCGA[41]) and integrative cluster C6 is characterized by mutation in *PIK3CA* (55.3%) that is enriched with Luminal A tumors (49% mutation, TCGA[41]). Moreover, the mutations are significantly associated across the six integrative clusters (p-value<0.001 for *TP53*, *PIK3CA and MAP3K1*; p-value = 0.011 for *GATA3*; Fisher Exact test). Graphical representation of this table has been provided with S7 Fig.

#### Glioblastoma

**Fig 4:**
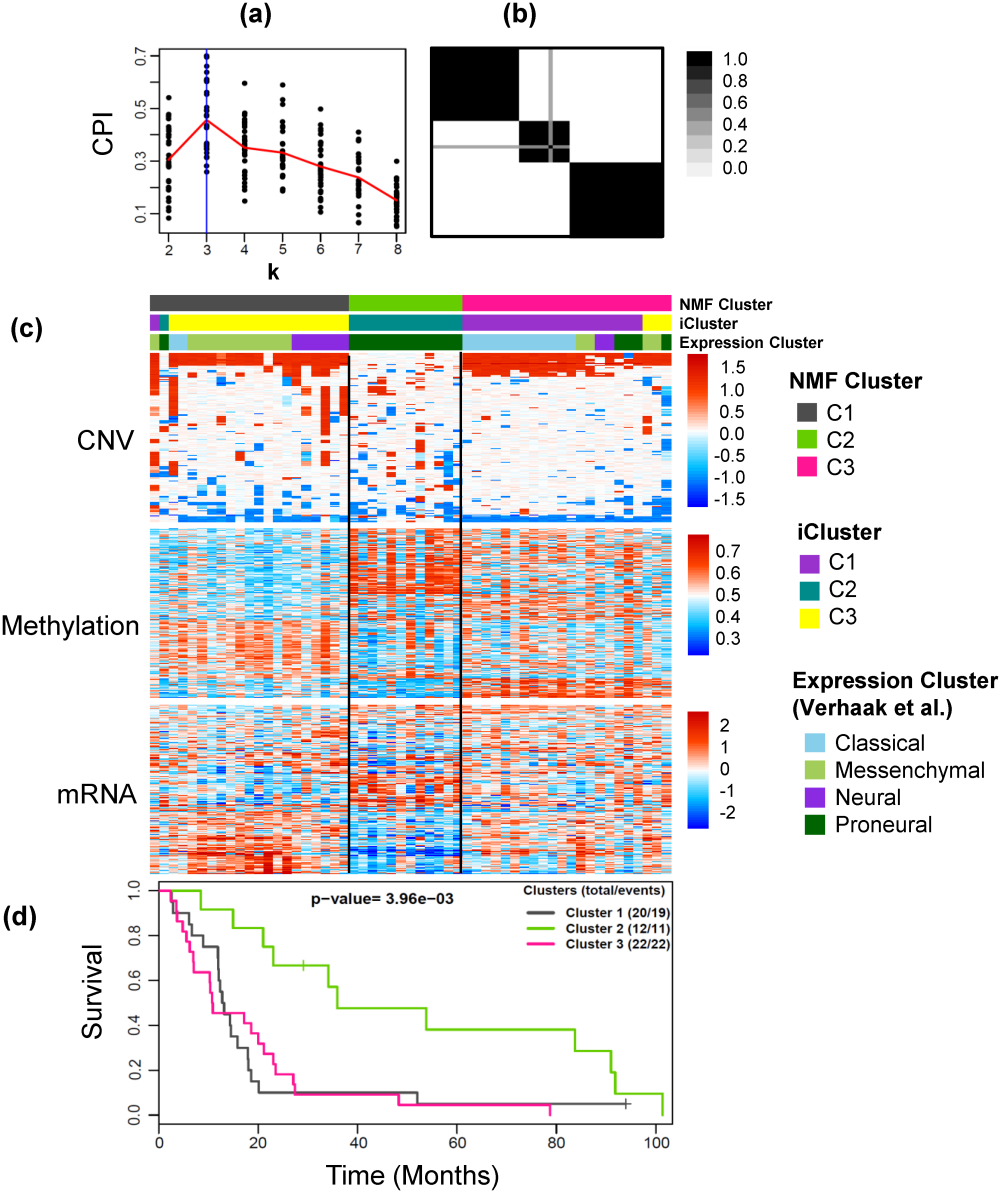
Example 2: Glioblastoma data. (a) Plot of *CPI* over the search range of number of clusters from 2 to 8 for 30 runs of intNMF algorithm at each *k*. The red line represents the mean values of *CPI* at each *k*. (b) The cluster pattern as shown by the consensus matrix. (c) Heatmap of three types of data, CNV, Methylation and mRNA with clustering assignment from intNMF, iCluster and Expression subtypes overlaid on top with legends on the side. (d) Kaplan Meier Survival curves with p-value from log-rank test.

The intNMF results in three optimum number of clusters, Fig 4(a) and 4(b). The weights used in the method were calculated as mentioned in the Methods section. The cross tabulation match with the expression-subtypes and iCluster-subtypes are shown in Table 2 and heatmaps of the three datasets are shown in Fig 4(c). There was very strong association between the clusters identified by intNMF and the previous clusters [37, 44]. The intNMF cluster C2 matched with the proneural-subtype and iCluster-C2. intNMF-C1 was enriched with Messenchymal and Neural, along with overlapping most with iCluster-C3. Lastly, intNMF-C3 tumors were enriched for Classical type tumors and match with most of iCluster-C1. Additionally, the cluster assignments from intNMF (Fig 4(d)), iCluster [37], and clusters based only on mRNA data [44] were associated with overall survival (intNMF clusters, p-value = 3.96×10^−3^; iCluster clusters, p-value = 1.0×10^−2^; mRNA only clusters, p-value = 1.84×10^−2^). Somatic mutations in a few genes highlighted by TCGA studies [45] and Verhaak et al.[44] have been presented in Table 2 as percentages of their presence in each integrative cluster. Consistent with previous studies [44], Proneural made integrative cluster C2 is characterized by mutations in *TP53*, Mesenchymal enriched integrative cluster C1 is characterized by mutations in *NF1*. Similar trends were seen in other gene mutations with *PIK3R1* and *PIK3CA* not present in integrative cluster C1 and *RB1* not present in C2. Except *EGFR*, none of the mutations were statistically significant across the integrative clusters.

**Table 2:** Cross tabulation of intNMF cluster subtypes with (i) Expression cluster subtypes [44] and (ii) iCluster subtypes [37] using Glioblastoma data. The summary table, followed by cross tabulation, represents somatic mutations in a few genes (highlighted by previous studies [44, 45]) presented as percentage of their presence in each of the three integrative clusters. Graphical representation of this table has been provided with S8 Fig.

		intNMF			Total
		C1	C2	C3	
**Expression** **Subtype**	**Classical**	2	0	12	**14**
	**Messenchymal**	12	0	4	**16**
	**Neural**	6	0	2	**8**
	**Proneural**	1	12	4	**17**
	**Total**	**21**	**12**	**22**	**55**
**iCluster**	**C1**	1	0	19	**20**
	**C2**	1	12	0	**13**
	**C3**	19	0	3	**22**
	**Total**	**21**	**12**	**22**	**55**
**Somatic** **Mutation**	**TP53 (%)**	30	66.7	31.8	
	**NF1 (%)**	30	16.7	4.5	
	**PTEN (%)**	25	8.3	31.8	
	**EGFR (%)**	5	8.3	31.8	
	**PIK3R1 (%)**	0	25	18.2	
	**PIK3CA (%)**	0	8.3	9.1	
	**RB1 (%)**	15	0	4.5	
	**ERBB2 (%)**	15	16.7	9.1	

## Discussion

A fundamental problem in many high dimensional data analysis is to find a suitable lower dimensional representation of the data. In this article, we have presented a clustering approach that integrates multiple data types collected on the same set of subjects to find such a representation. In application of intNMF to two cancer studies from TCGA, we demonstrated that intNMF is efficient in extracting the clusters inherent in the data. Both examples show that the subtypes identified by the intNMF method match closely with the subtypes identified by previous studies. A challenge of any clustering methods is the unsupervised nature of the problem; that is, how many clusters are inherent in the data. To address this issue, we describe a resampling based cross-validation method of model selection to find out optimum number of clusters. Most importantly, the proposed intNMF method does not require any statistical distribution assumption of the data, and therefore has robust application to studies involving diverse data types. The proposed intNMF clustering method is different than the integrative NMF method proposed by Zhang et al. [11] with respect to both purpose and algorithm utilized by the methods. The method by Zhang et al. was designed to identify the modules (blocks of data) comprising the correlated variables while the proposed method in this article carries out sample clustering and subtype discovery. Zhang et al. utilize multiplicative update rule while the proposed method uses even better alternating least squares algorithm.

During the flow of genetic information within a biological system, the DNA is transcribed to mRNA and mRNA is translated to protein. Also epigenetic modifications of genes by methylation and deletions/amplifications of sections of genome further alter the gene expression. During this molecular process, the latent structure may or may not be seen consistently across all genomic assays. Therefore platform specific data clustering may not be able to reveal such latent structure. The integrative clustering not only strengthens this weakness but also improves the statistical power of detection. Furthermore, all types of data may not be equally informative and therefore context specific approach may be necessary in order to assign the relative importance (weights) for the data in the clustering method when more is known about the underlying properties of the data. The proposed integrative clustering approach allows such user specified weights in the method.

In summary, as multiple types of data are increasingly available due to high throughput technologies, an essence of integrative method of clustering has been more evident and attention has been diverted appreciably towards that direction. To this end, we propose unified framework of clustering using intNMF for classifying the disease into distinct subtypes. Application of the method in both simulated and real data examples show that the method performs as well as or better than existing methods by adding more flexibility and robustness for using diverse types of data. The method is implemented in an R package available in CRAN and is named *intNMF*.

## Supporting information

S1 File Supplementary materials. (PDF)

S2 File Complete set of supplementary figures. (PDF)

S1 Fig Finding optimum number of clusters. Plots showing the comparison of five different methods of finding optimum number of clusters on the dataset generated using varying effect sizes for true number of clusters k = 4. First row represents silhouette width over k = 2:8 for each of five different scenarios of true clusters 2, 3, 4, 5 and 6 over 30 runs of simulation. The average value of the silhouette widths over 30 runs are overlaid on the plots as line. Cophenetic correlation, Dispersion, Residual Sums of Squares and Cluster Prediction Index are shown on second, third, fourth and fifth rows respectively.
(PDF)

S2 Fig Signal to noise ratio. Plots showing the signal to noise ratio (mean/sd) for the four types of measures, Silhouette, Cophenetic correlation, Dispersion and Cluster Prediction Index for finding optimum number of clusters for cluster mean shift effect size of 3.5 and varying scenarios of true number of clusters. Cluster prediction index has the best ability of finding optimum number of clusters with maximum value at the true number of clusters with best precision.
(PDF)

S3 Fig Signal to noise ratio. Plots showing the signal to noise ratio (mean/sd) for the four types of measures, silhouette, cophenetic correlation, dispersion and cluster prediction index for finding optimum number of clusters for true number of clusters 4 and varying sizes of cluster mean shift effect. Cluster prediction index has the best ability of finding optimum number of clusters with maximum value at the true number of clusters with best precision if the cluster shift effect size is adequate.
(PDF)

S4 Fig Comparison of intNMF and iCluster over varying k. First row represents the plot of *purity* for intNMF (red) and iCluster (blue) and second row represents plot of *entropy* for intNMF and iCluster. Purity is expected to result in maximum and entropy is expected to result in minimum at true number of clusters.
(PDF)

S5 Fig Comparison of intNMF and iCluster over varying effect sizes. First row represents the cluster prediction index, second row represents the plot of proportion of deviance (POD) given by iCluster method, third row represents adjusted rand index between (i) true and intNMF-clusters (red), (ii) true and iCluster-clusters (blue) and (iii) intNMF-clusters and iCluster-clusters (green), fourth row represents the plot of purity for intNMF and iCluster and fifth row represents plot of entropy for intNMF and iCluster. The POD and entropy are expected to result in minimum at true number of clusters. In other plots, maximum is expected at true number of clusters.
(PDF)

S6 Fig Block structure as shown by iCluster and Kaplan Meier survival curves among the three iCluster subgroups using TCGA breast cancer data. (PDF)

S7 Fig Comparison of intNMF subtypes for breast cancer with and (a) TCGA subtypes and (b) iCluster subtypes. These figures are the graphical representation of Table 1 in the paper.
(PDF)

S8 Fig Comparison of intNMF subtypes for glioblastoma with and (a) Expression subtypes and (b) iCluster subtypes. These figures are the graphical representation of Table 2 in the paper.
(PDF)

S1 Table Cross tabulation of iCluster subtypes with TCGA subtypes using multiplatform Breast cancer data. (PDF)
