# Supplementary material for: Integrative clustering of multi-level ‘omic data based on non-negative matrix factorization algorithm
Source: PLoS One. 2017 May 1;12(5):e0176278. doi: 10.1371/journal.pone.0176278 (PMC5411077; doi:10.1371/journal.pone.0176278)
Supplement: S1 File — (PDF) [file pone.0176278.s001.pdf]

Supplementary Materials

1. **Derivation of solution for common *W* matrix:**

Suppose $\boldsymbol{X}^i$, $i=1, 2, \ldots, m$ be matrices representing m types of genomic assays profiled on same set of n samples with p_i features, $\boldsymbol{W}$ be the common basis matrix, $\boldsymbol{H}^i$, $i=1,\ldots,m$ be the individual data specific coefficient matrices and $\theta^i \geq 0$, $i=1,\ldots,m$ be the weights for several data types. Then we can write

$$\boldsymbol{X}^i_{n\times p_i} \approx \boldsymbol{W}_{n\times k}\boldsymbol{H}^i_{k\times p_i} \quad i = 1,2,\ldots m$$

Where $\boldsymbol{W}_{n\times k} \geq 0, \boldsymbol{H}^i_{k\times p_i} \geq 0,\ i = 1,2,\ldots m$

The intNMF problem minimizes the following objective function

$$Q = \sum_{i=1}^{m} \theta^i\left(\boldsymbol{X}^i_{n\times p_i} - \boldsymbol{W}_{n\times k}\boldsymbol{H}^i_{k\times p_i}\right)^2$$

$$Q = \sum_{i=1}^{m} \left[\sqrt{\theta^i}\left(\boldsymbol{X}^i_{n\times p_i} - \boldsymbol{W}_{n\times k}\boldsymbol{H}^i_{k\times p_i}\right)\right]^2$$

There is no global minimum solution for NMF problem. Therefore, it is unrealistic to design an algorithm to find the global minimum of Q. We derive approximate solution for $\boldsymbol{W}$. Since our objective is to approximately factorize $\boldsymbol{X}^i_{n\times p_i}$ in to $\boldsymbol{W}_{n\times k}$ and $\boldsymbol{H}^i_{k\times p_i}$ matrices for $i=1,2,\ldots,m$, and that each of the m terms in the above equation is strictly positive we can write,

$$\sqrt{\theta^i}\left(\boldsymbol{X}^i_{n\times p_i} - \boldsymbol{W}_{n\times k}\boldsymbol{H}^i_{k\times p_i}\right) \approx \boldsymbol{0}_{n\times p_i}$$

$$\sqrt{\theta^i}\boldsymbol{X}^i_{n\times p_i} \approx \sqrt{\theta^i}\,\boldsymbol{W}_{n\times k}\boldsymbol{H}^i_{k\times p_i}$$

For simplicity, writing the expression in the form of equation

$$\sqrt{\theta^i}\boldsymbol{X}^i_{n\times p_i} = \sqrt{\theta^i}\,\boldsymbol{W}_{n\times k}\boldsymbol{H}^i_{k\times p_i} + \boldsymbol{C}^i_{n\times p_i}$$

Where $\boldsymbol{C}^i_{n\times p_i}$ is matrix of constants having small numbers.

Multiplying both sides by transpose of $\boldsymbol{H}^i_{k\times p_i}$ and summing over i

$$\sum_{i=1}^{m}\sqrt{\theta^i}\,\boldsymbol{X}^i_{n\times p_i}\left(\boldsymbol{H}^i\right)^T_{p_i\times k} = \sum_{i=1}^{m}\sqrt{\theta^i}\,\boldsymbol{W}_{n\times k}\boldsymbol{H}^i_{k\times p_i}\left(\boldsymbol{H}^i\right)^T_{p_i\times k} + \sum_{i=1}^{m}\boldsymbol{C}^i_{n\times p_i}\left(\boldsymbol{H}^i\right)^T_{p_i\times k}$$

$$\sum_{i=1}^{m}\sqrt{\theta^i}\,\boldsymbol{X}^i_{n\times p_i}\left(\boldsymbol{H}^i\right)^T_{p_i\times k} = \sum_{i=1}^{m}\sqrt{\theta^i}\,\boldsymbol{W}_{n\times k}\boldsymbol{H}^i_{k\times p_i}\left(\boldsymbol{H}^i\right)^T_{p_i\times k} + \boldsymbol{C}'_{n\times k}$$

Where, $\boldsymbol{C}'_{n\times k} = \sum_{i=1}^{m}\boldsymbol{C}^i_{n\times p_i}\left(\boldsymbol{H}^i\right)^T_{p_i\times k}$ is a constant. Note that $\boldsymbol{H}^i$, i=1,…,m are computed separately.

$$\sum_{i=1}^{m}\sqrt{\theta^i}\,\boldsymbol{X}^i_{n\times p_i}\left(\boldsymbol{H}^i\right)^T_{p_i\times k} = \boldsymbol{W}_{n\times k}\sum_{i=1}^{m}\sqrt{\theta^i}\left(\boldsymbol{H}^i\left(\boldsymbol{H}^i\right)^T\right)_{k\times k} + \boldsymbol{C}'_{n\times k}$$

$$\boldsymbol{W}_{n\times k} = \left(\sum_{i=1}^{m}\sqrt{\theta^i}\,\boldsymbol{X}^i_{n\times p_i}(\boldsymbol{H}^i)^T_{p_i\times k}\right)\left(\sum_{i=1}^{m}\sqrt{\theta^i}(\boldsymbol{H}^i(\boldsymbol{H}^i)^T)_{k\times k}\right)^{-1} - \boldsymbol{C}'_{n\times k}\left(\sum_{i=1}^{m}\sqrt{\theta^i}(\boldsymbol{H}^i(\boldsymbol{H}^i)^T)_{k\times k}\right)^{-1}$$

$$\boldsymbol{W}^T_{k\times n} = \left[\left(\sum_{i=1}^{m}\sqrt{\theta^i}\,\boldsymbol{X}^i_{n\times p_i}\left(\boldsymbol{H}^i\right)^T_{p_i\times k}\right)\left(\sum_{i=1}^{m}\sqrt{\theta^i}\left(\boldsymbol{H}^i\left(\boldsymbol{H}^i\right)^T\right)_{k\times k}\right)^{-1}\right]^T + \boldsymbol{C}''_{k\times n}$$

Where $\boldsymbol{C}''_{k\times n} = \left[\ -\boldsymbol{C}'_{n\times k}\left(\sum_{i=1}^{m}\sqrt{\theta^i}(\boldsymbol{H}^i(\boldsymbol{H}^i)^T)_{k\times k}\right)^{-1}\right]^T$ constant matrix. Writing back in approximate form,

$$\boldsymbol{W}^T_{k\times n} \approx \left[\left(\sum_{i=1}^{m}\sqrt{\theta^i}\ \left(\boldsymbol{H}^i\left(\boldsymbol{H}^i\right)^T\right)_{k\times k}\right)^T\right]^{-1}\left[\left(\sum_{i=1}^{m}\sqrt{\theta^i}\,\boldsymbol{X}^i_{n\times p_i}\left(\boldsymbol{H}^i\right)^T_{p_i\times k}\ \right)^T\right]$$

This equation can be solved using non negative alternating least square method. Finally $\boldsymbol{W}_{n\times k}$ is obtained by the transpose of $\boldsymbol{W}^T_{k\times n}$.

2. Solution for H matrices

$\boldsymbol{H}^i$, *i=1,…,m* matrices are solved using non-negativity constraint least square method.

$$\boldsymbol{H}^i_{k\times p_i} \approx (\boldsymbol{W}^T\boldsymbol{W})^{-1}_{k\times k}\boldsymbol{W}^T_{k\times n}\boldsymbol{X}^i_{n\times p_i}\quad i = 1,2,\dots,m$$

$$\text{such that } \boldsymbol{H}^i_{k\times p_i} \geq 0$$

3. Convergence of intNMF to standard NMF when $m \to 1$

For, *i=1,…,m* the solution for W is given by

$$\boldsymbol{W}_{k\times n}^{T} \approx \left[\left(\sum_{i=1}^{m}\sqrt{\theta^{i}}\ \left(\boldsymbol{H}^{i}\left(\boldsymbol{H}^{i}\right)^{T}\right)_{k\times k}\right)^{T}\right]^{-1}\left[\left(\sum_{i=1}^{m}\sqrt{\theta^{i}}\,\boldsymbol{X}_{n\times p_i}^{i}\left(\boldsymbol{H}^{i}\right)_{p_i\times k}^{T}\right)^{T}\right]$$

If $m \to 1$,

$$\boldsymbol{W}_{k\times n}^{T} \approx \left[\left(\sum_{i=1}^{1}\sqrt{\theta^{i}}\ \left(\boldsymbol{H}^{i}\left(\boldsymbol{H}^{i}\right)^{T}\right)_{k\times k}\right)^{T}\right]^{-1}\left[\left(\sum_{i=1}^{1}\sqrt{\theta^{i}}\,\boldsymbol{X}_{n\times p_i}^{i}\left(\boldsymbol{H}^{i}\right)_{p_i\times k}^{T}\right)^{T}\right]$$

There is no need of weight for a single data. i.e. $\theta^{i} \to 1$

$$\boldsymbol{W}_{k\times n}^{T} \approx \left[\left(\sum_{i=1}^{1}\sqrt{1}\ \left(\boldsymbol{H}^{i}\left(\boldsymbol{H}^{i}\right)^{T}\right)_{k\times k}\right)^{T}\right]^{-1}\left[\left(\sum_{i=1}^{1}\sqrt{1}\,\boldsymbol{X}_{n\times p_i}^{i}\left(\boldsymbol{H}^{i}\right)_{p_i\times k}^{T}\right)^{T}\right]$$

$$= [(\ (\boldsymbol{H}^{1}(\boldsymbol{H}^{1})^{T})_{k\times k})^{T}]^{-1}\left[\left(\boldsymbol{X}_{n\times p_1}^{1}(\boldsymbol{H}^{1})_{p_1\times k}^{T}\ \right)^{T}\right]$$

$$= (\boldsymbol{H}^{1}(\boldsymbol{H}^{1})^{T})_{k\times k}^{-1}\ \boldsymbol{H}_{k\times p_1}^{1}\ \ (\boldsymbol{X}^{1})_{p_1\times n}^{T}$$

Which is solution of

$$(\boldsymbol{X}^{1})_{p_1\times n}^{T} \approx (\boldsymbol{H}^{1})_{p_1\times k}^{T}\boldsymbol{W}_{k\times n}^{T}$$

$$\Rightarrow\ \boldsymbol{X}_{n\times p_1}^{1} \approx \boldsymbol{W}_{n\times k}\boldsymbol{H}_{k\times p_1}^{1}$$

For simplicity, writing p for p_1, $\boldsymbol{X}$ for $\boldsymbol{X}^{1}$ and $\boldsymbol{H}$ for $\boldsymbol{H}^{1}$.

$$\boldsymbol{X}_{n\times p} \approx \boldsymbol{W}_{n\times k}\ \boldsymbol{H}_{n\times p}$$

Which is standard NMF problem.

(Complete set of supplementary figures are provided separately with **S2 File. Complete set of Supplementary Figures**).
