# Supplementary material for: Integrative clustering of multi-level ‘omic data based on non-negative matrix factorization algorithm
Source: PLoS One. 2017 May 1;12(5):e0176278. doi: 10.1371/journal.pone.0176278 (PMC5411077; doi:10.1371/journal.pone.0176278)
Supplement: S2 File — (PDF) [file pone.0176278.s002.pdf]

Complete set of supplementary Figures:

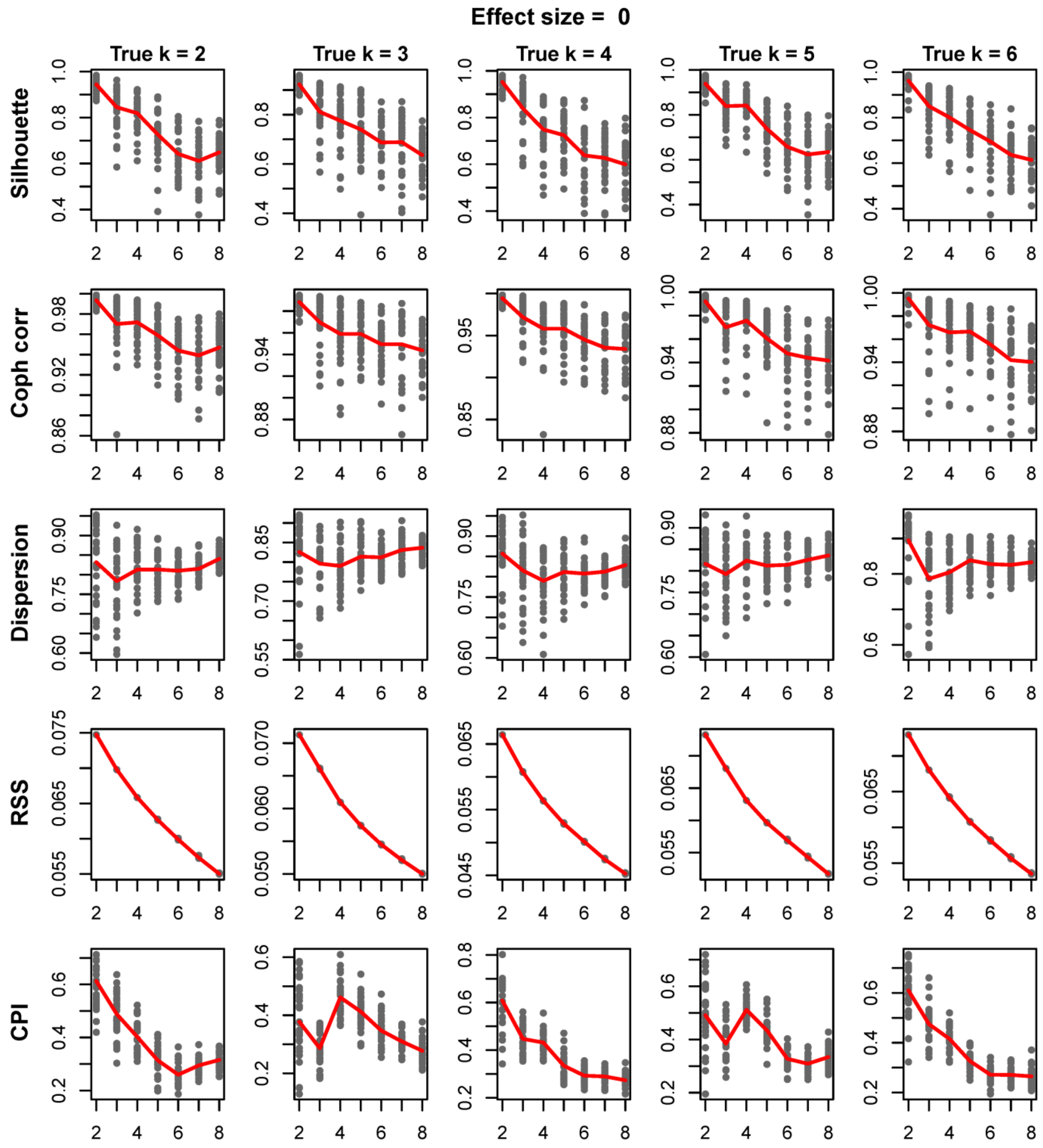

S6 Fig: Finding Optimum number of clusters with effect size 0. The plots show the values of the five parameters over k = 2:8 for each of five different scenarios of true clusters 2, 3, 4, 5 and 6 over 30 runs of simulation. The average values of the parameters over 30 runs are overlaid on the plots as a line.

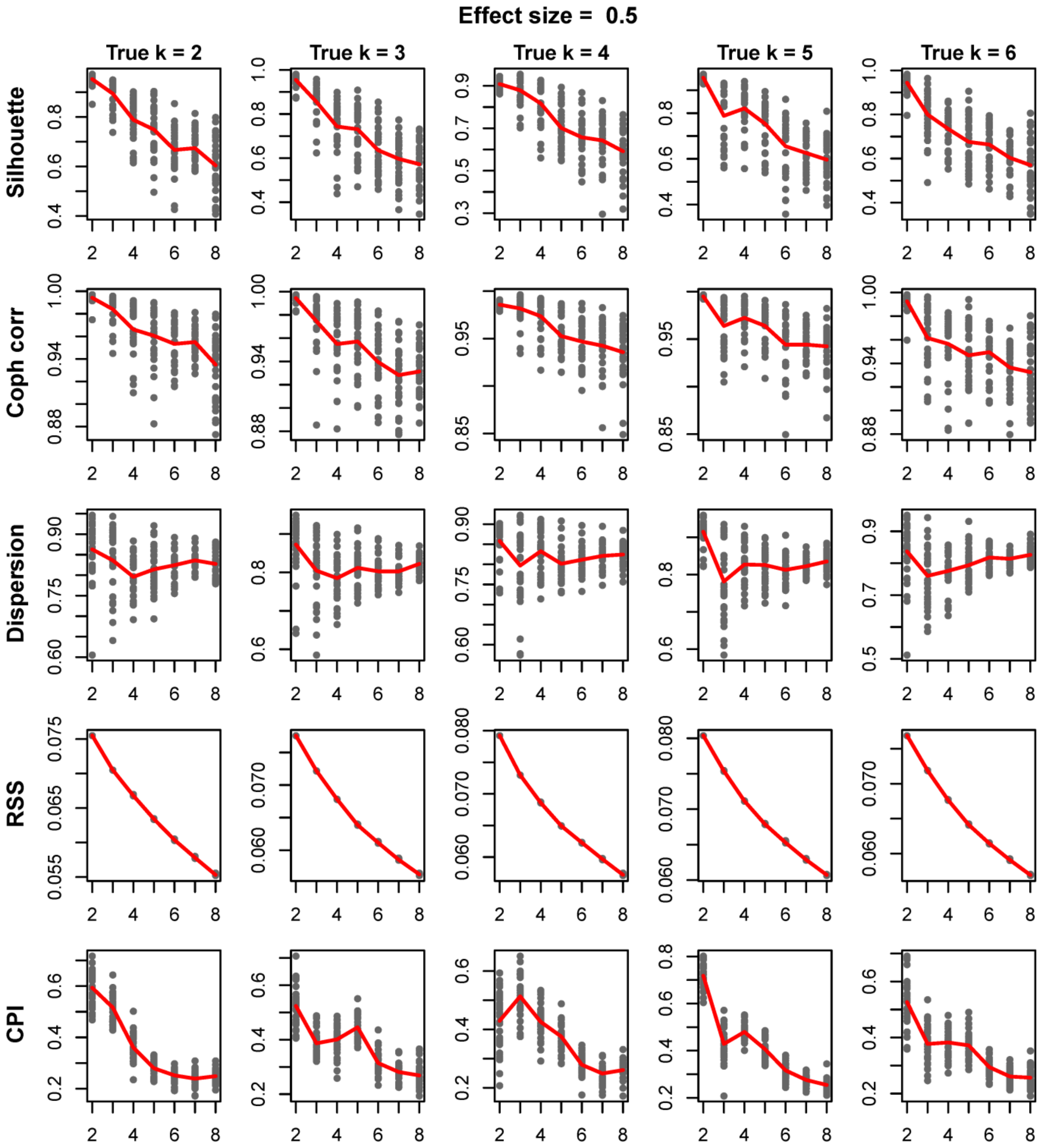

S7 Fig: Finding Optimum number of clusters with effect size 0.5. The plots show the values of the five parameters over k = 2:8 for each of five different scenarios of true clusters 2, 3, 4, 5 and 6 over 30 runs of simulation. The average values of the parameters over 30 runs are overlaid on the plots as a line.

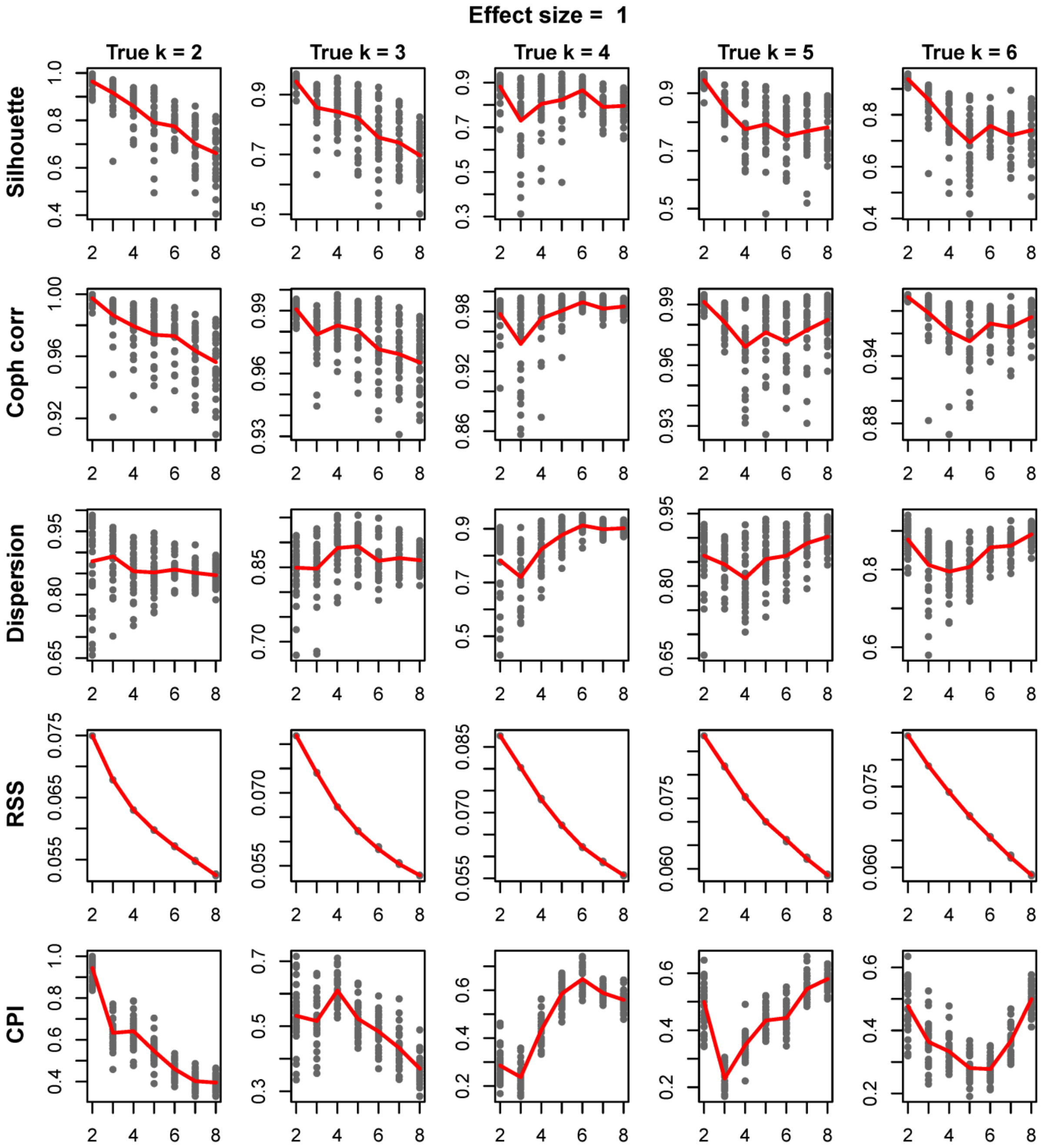

S8 Fig: Finding Optimum number of clusters with effect size 1.0. The plots show the values of the five parameters over k = 2:8 for each of five different scenarios of true clusters 2, 3, 4, 5 and 6 over 30 runs of simulation. The average values of the parameters over 30 runs are overlaid on the plots as a line.

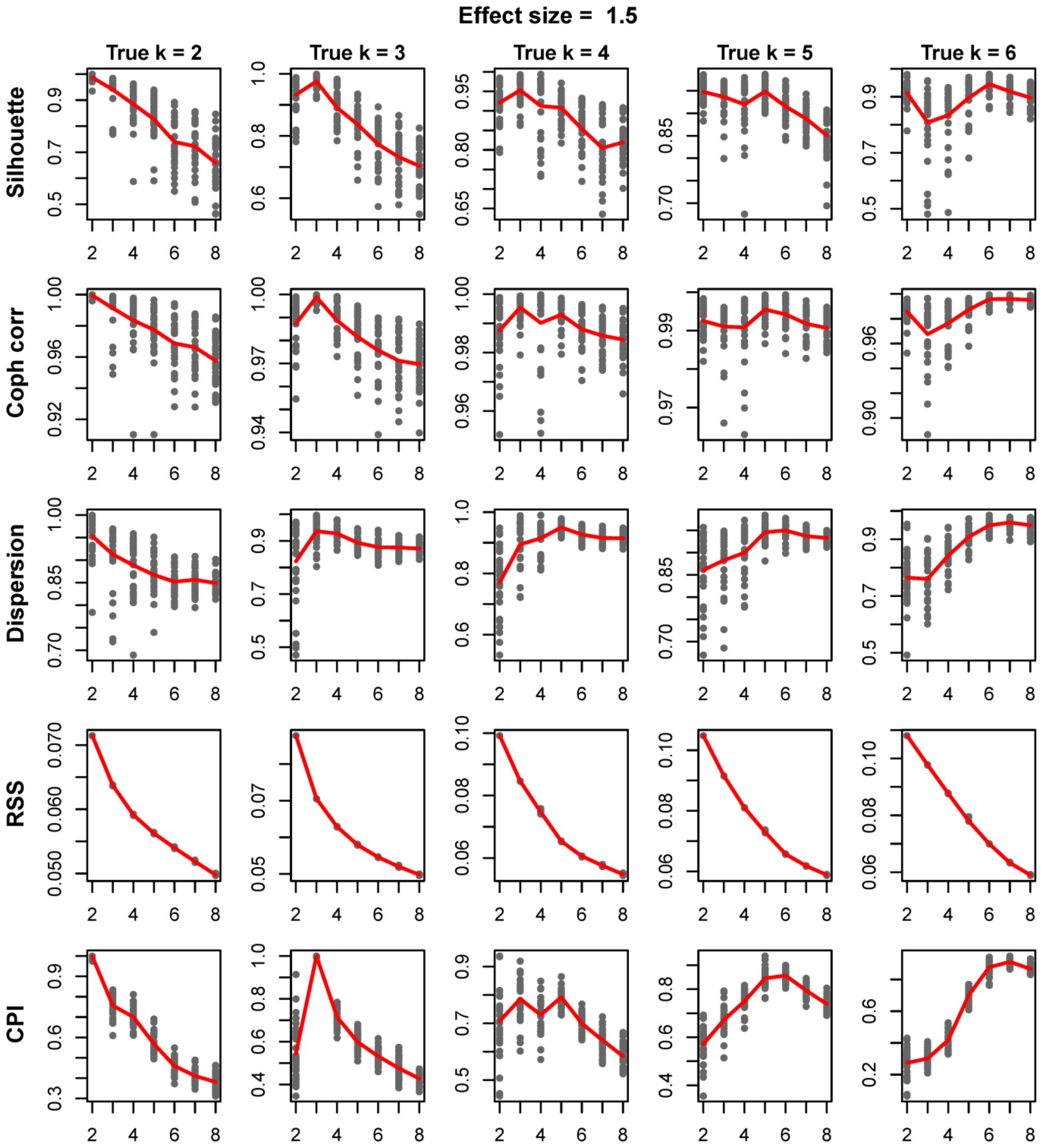

S9 Fig: Finding Optimum number of clusters with effect size 1.5. The plots show the values of the five parameters over k = 2:8 for each of five different scenarios of true clusters 2, 3, 4, 5 and 6 over 30 runs of simulation. The average values of the parameters over 30 runs are overlaid on the plots as a line.

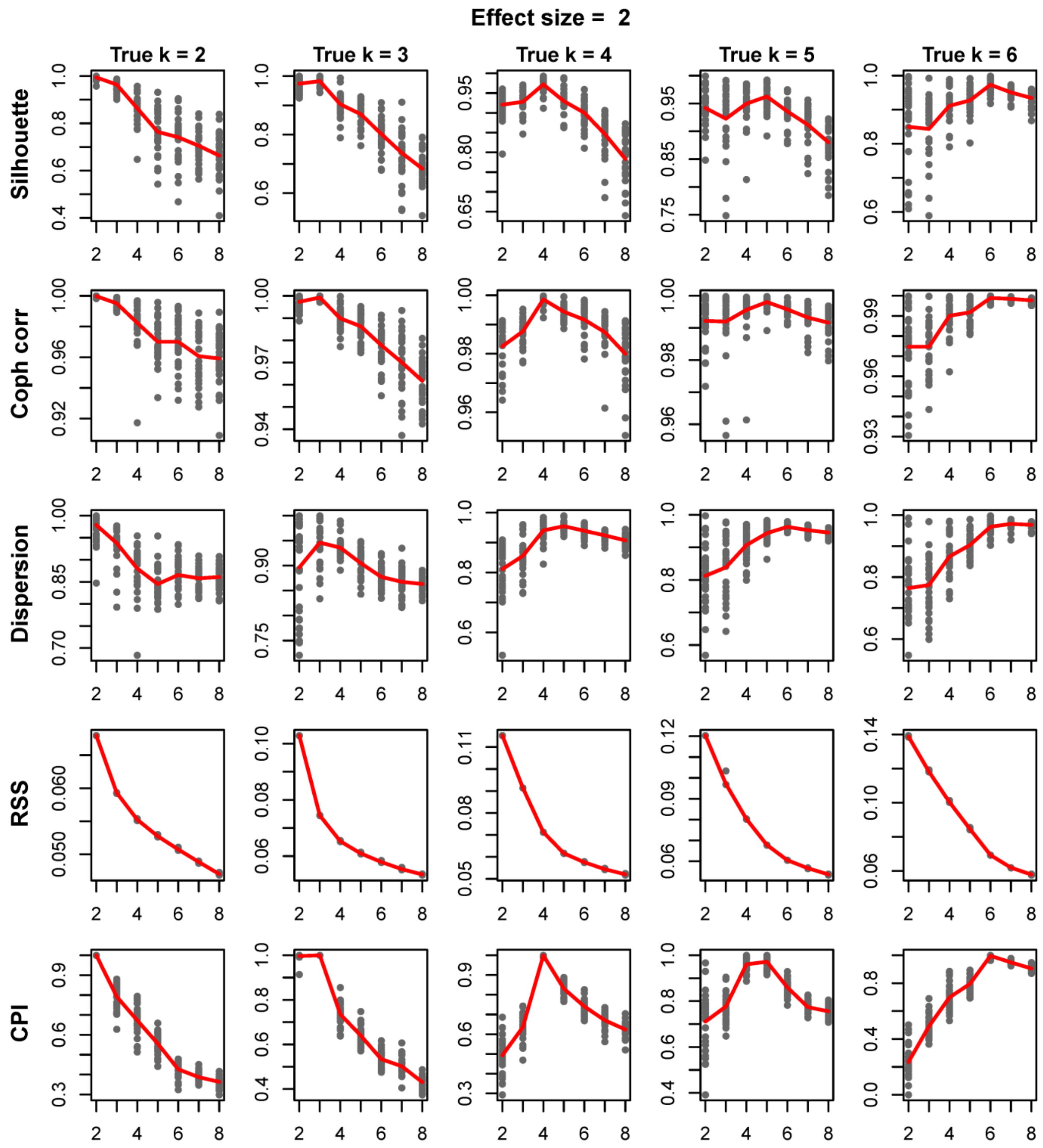

S10 Fig: Finding Optimum number of clusters with effect size 2.0. The plots show the values of the five parameters over k = 2:8 for each of five different scenarios of true clusters 2, 3, 4, 5 and 6 over 30 runs of simulation. The average values of the parameters over 30 runs are overlaid on the plots as a line.

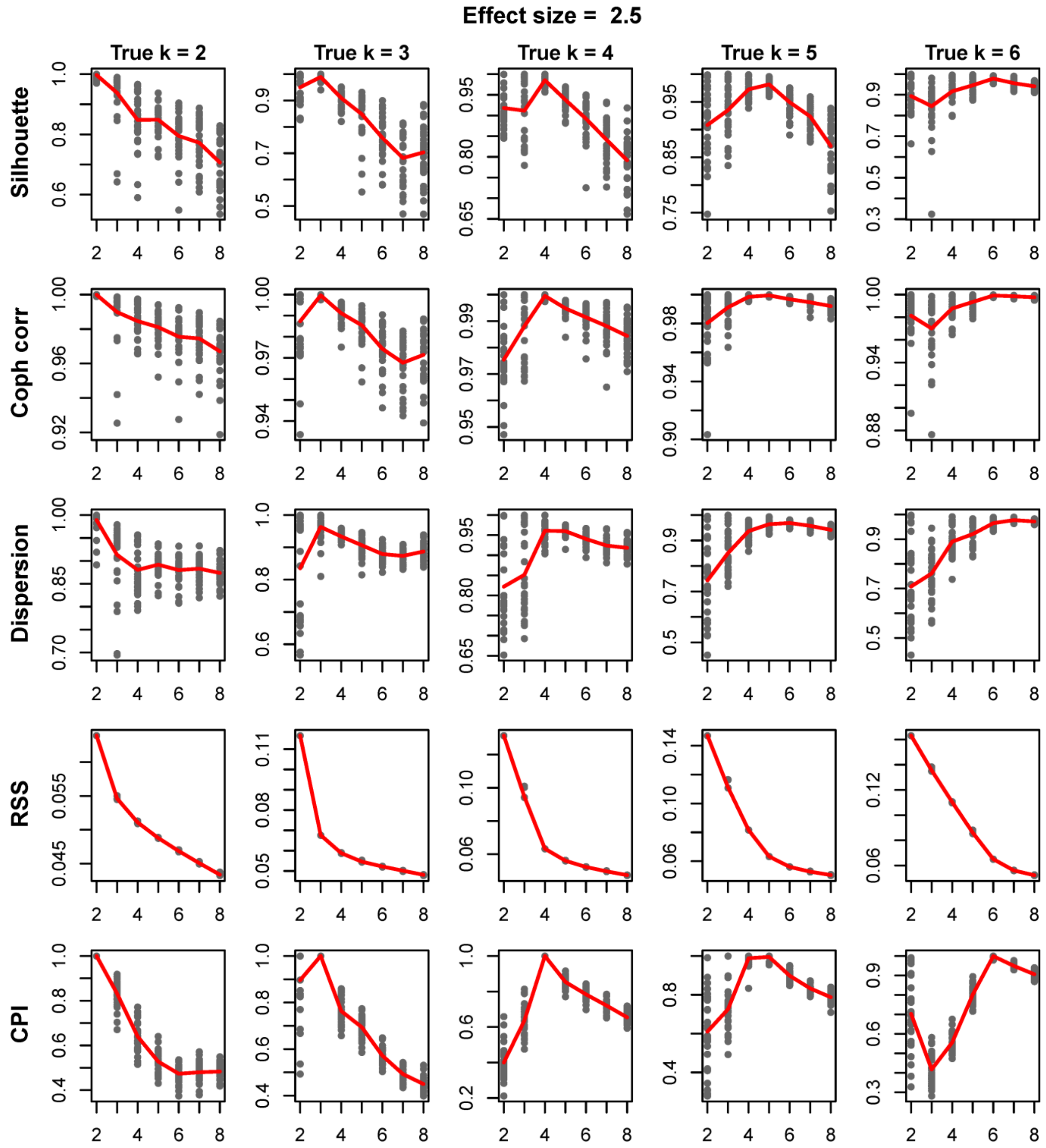

S11 Fig: Finding Optimum number of clusters with effect size 2.5. The plots show the values of the five parameters over k = 2:8 for each of five different scenarios of true clusters 2, 3, 4, 5 and 6 over 30 runs of simulation. The average values of the parameters over 30 runs are overlaid on the plots as a line.

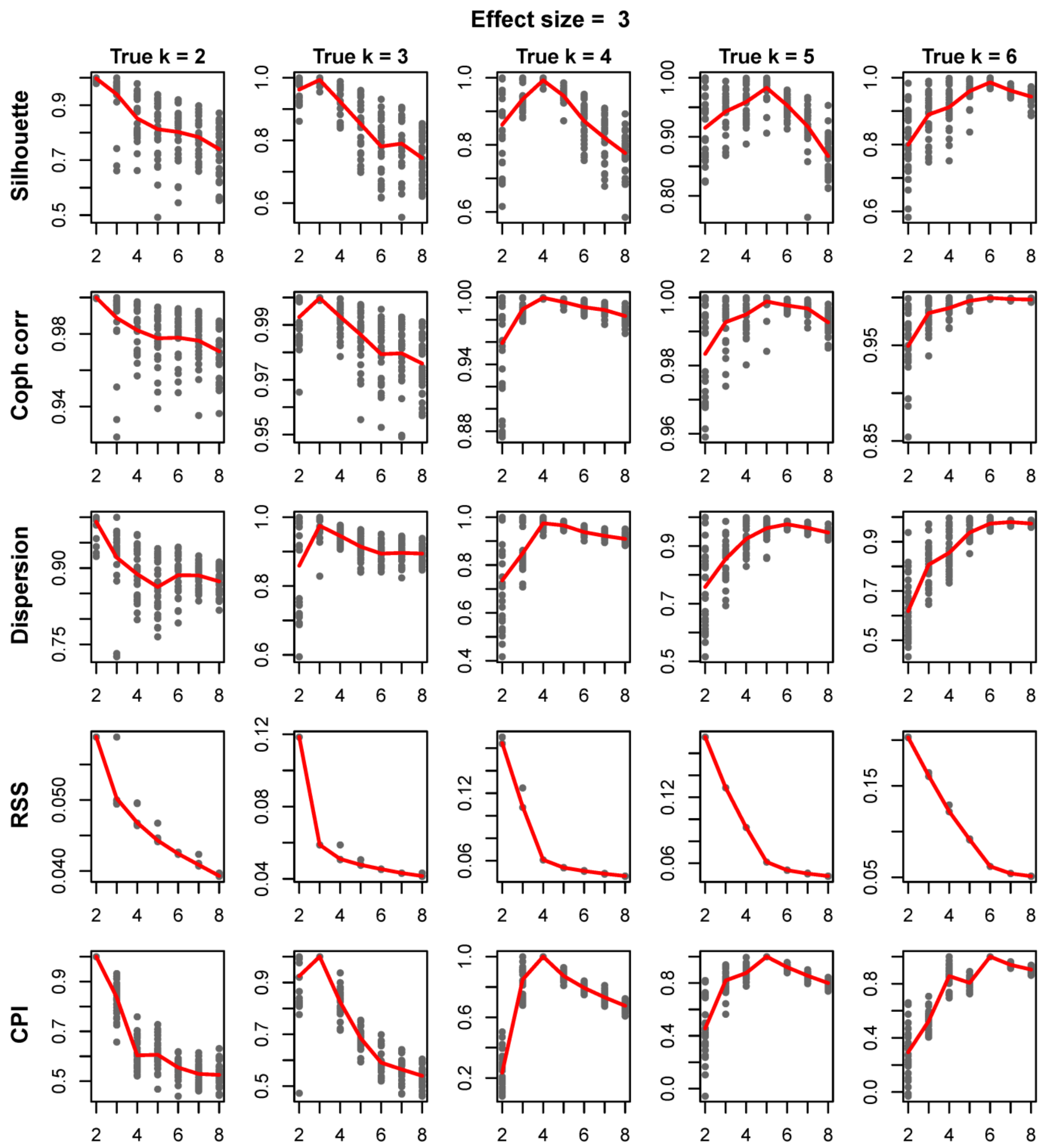

S12 Fig: Finding Optimum number of clusters with effect size 3.0. The plots show the values of the five parameters over k = 2:8 for each of five different scenarios of true clusters 2, 3, 4, 5 and 6 over 30 runs of simulation. The average values of the parameters over 30 runs are overlaid on the plots as a line.

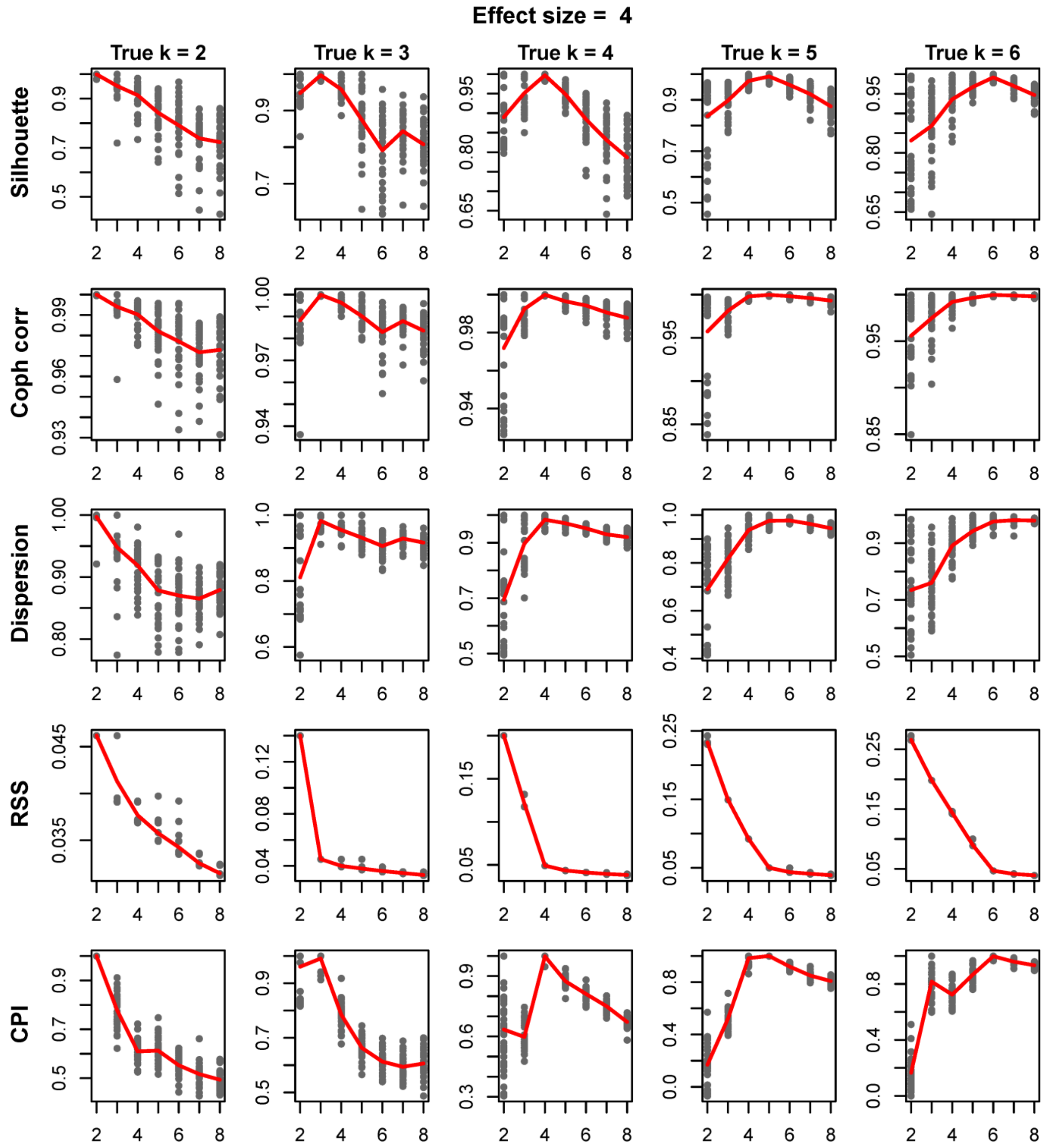

S13 Fig: Finding Optimum number of clusters with effect size 4.0. The plots show the values of the five parameters over k = 2:8 for each of five different scenarios of true clusters 2, 3, 4, 5 and 6 over 30 runs of simulation. The average values of the parameters over 30 runs are overlaid on the plots as a line.

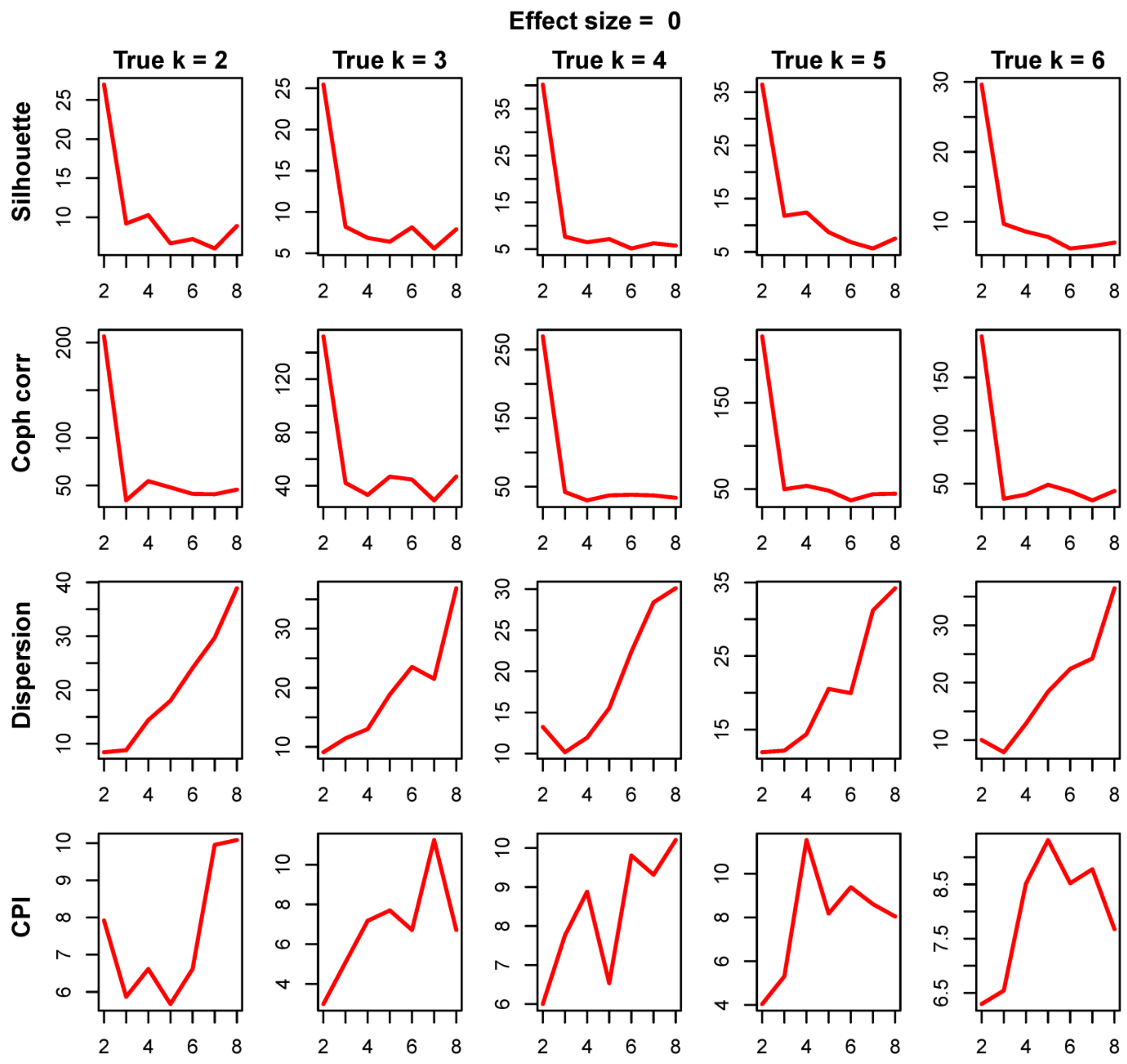

S14 Fig: Signal to noise ratio. Plots showing the signal to noise ratio (mean/sd) for the four parameters for finding optimum number of clusters for cluster mean shift effect size of 0.0 and varying scenarios of true number of clusters.

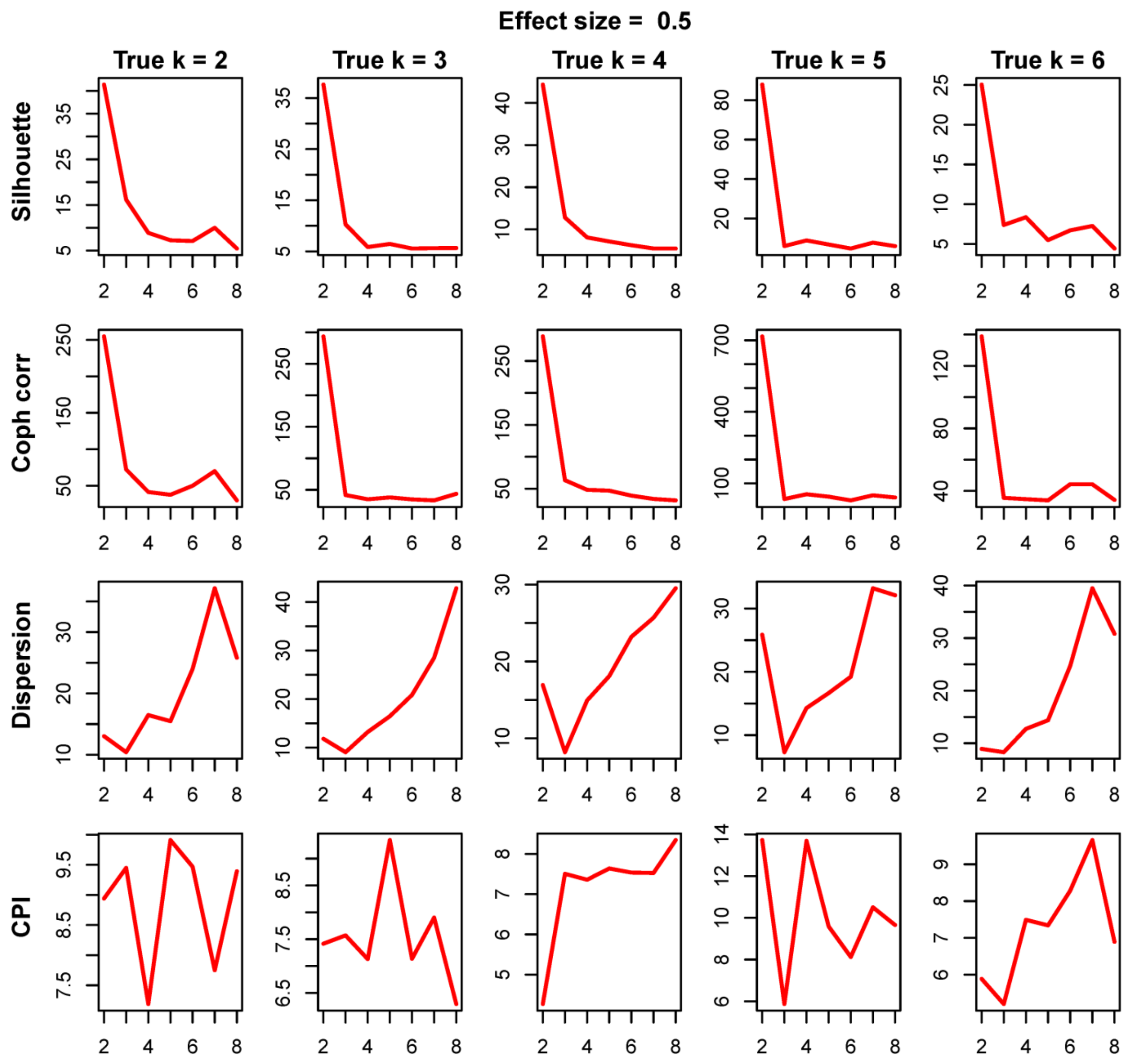

S15 Fig: Signal to noise ratio. Plots showing the signal to noise ratio (mean/sd) for the four parameters for finding optimum number of clusters for cluster mean shift effect size of 0.5 and varying scenarios of true number of clusters.

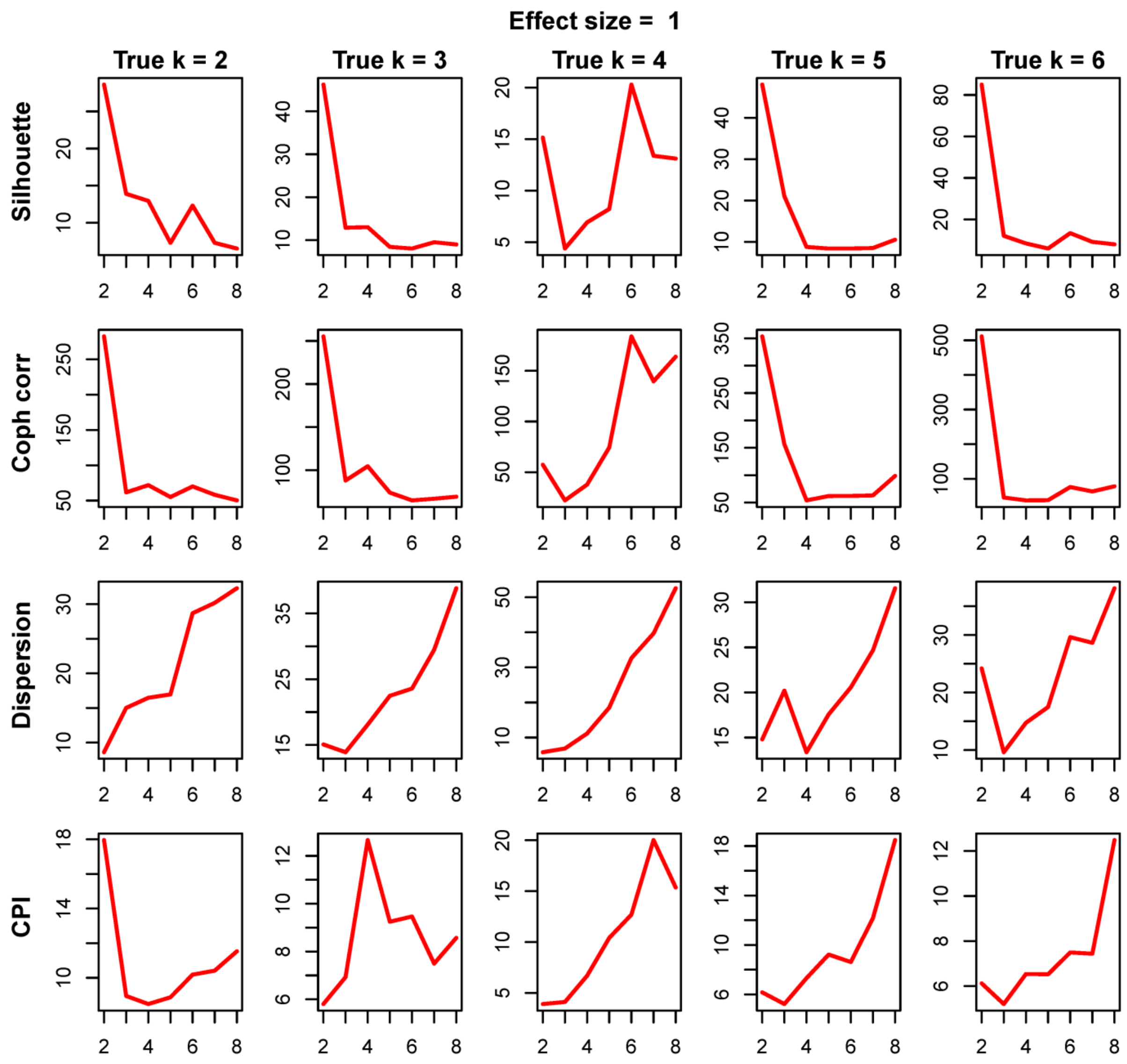

S16 Fig: Signal to noise ratio. Plots showing the signal to noise ratio (mean/sd) for the four parameters for finding optimum number of clusters for cluster mean shift effect size of 1.0 and varying scenarios of true number of clusters.

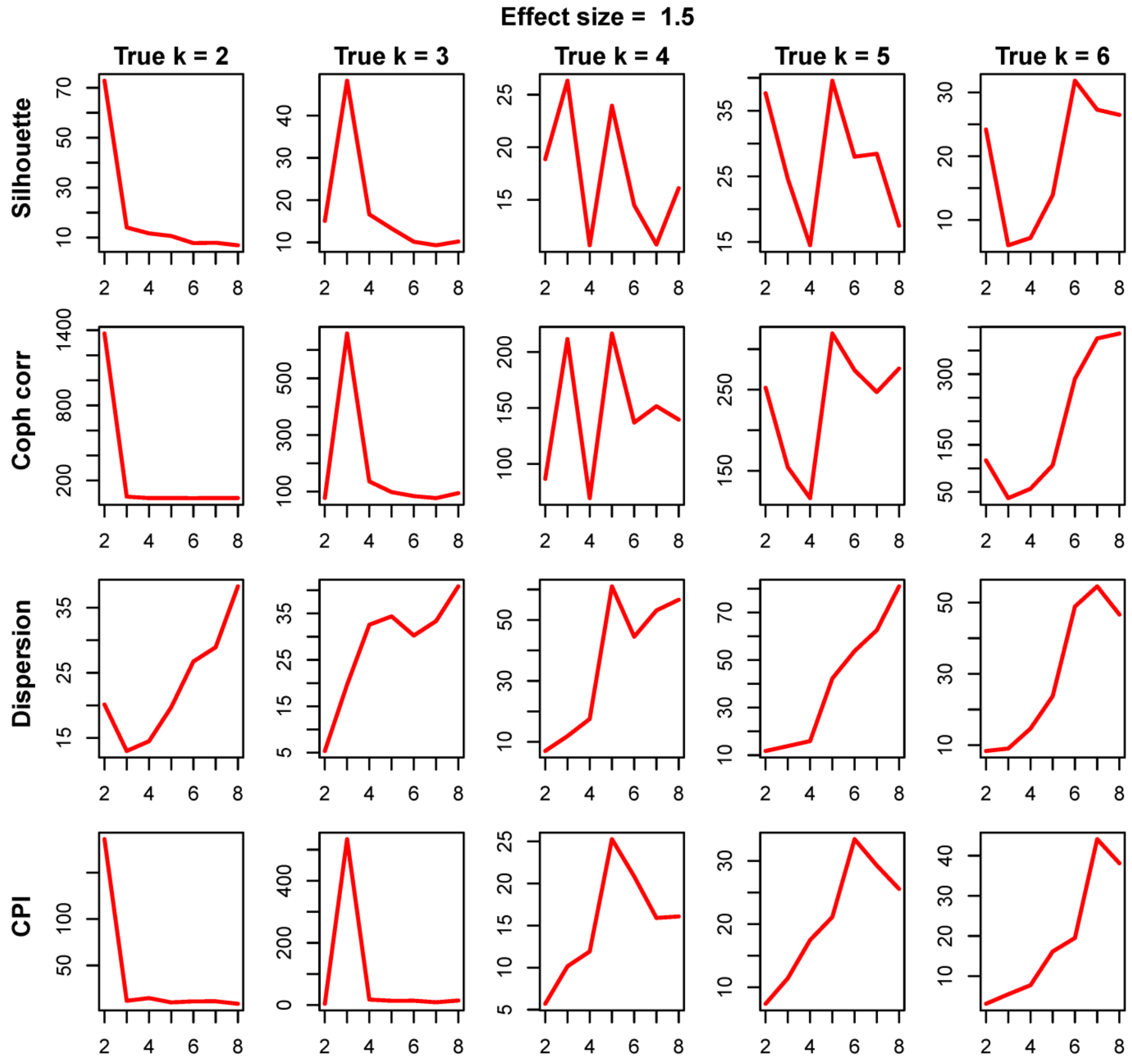

S17 Fig: Signal to noise ratio. Plots showing the signal to noise ratio (mean/sd) for the four parameters for finding optimum number of clusters for cluster mean shift effect size of 1.5 and varying scenarios of true number of clusters.

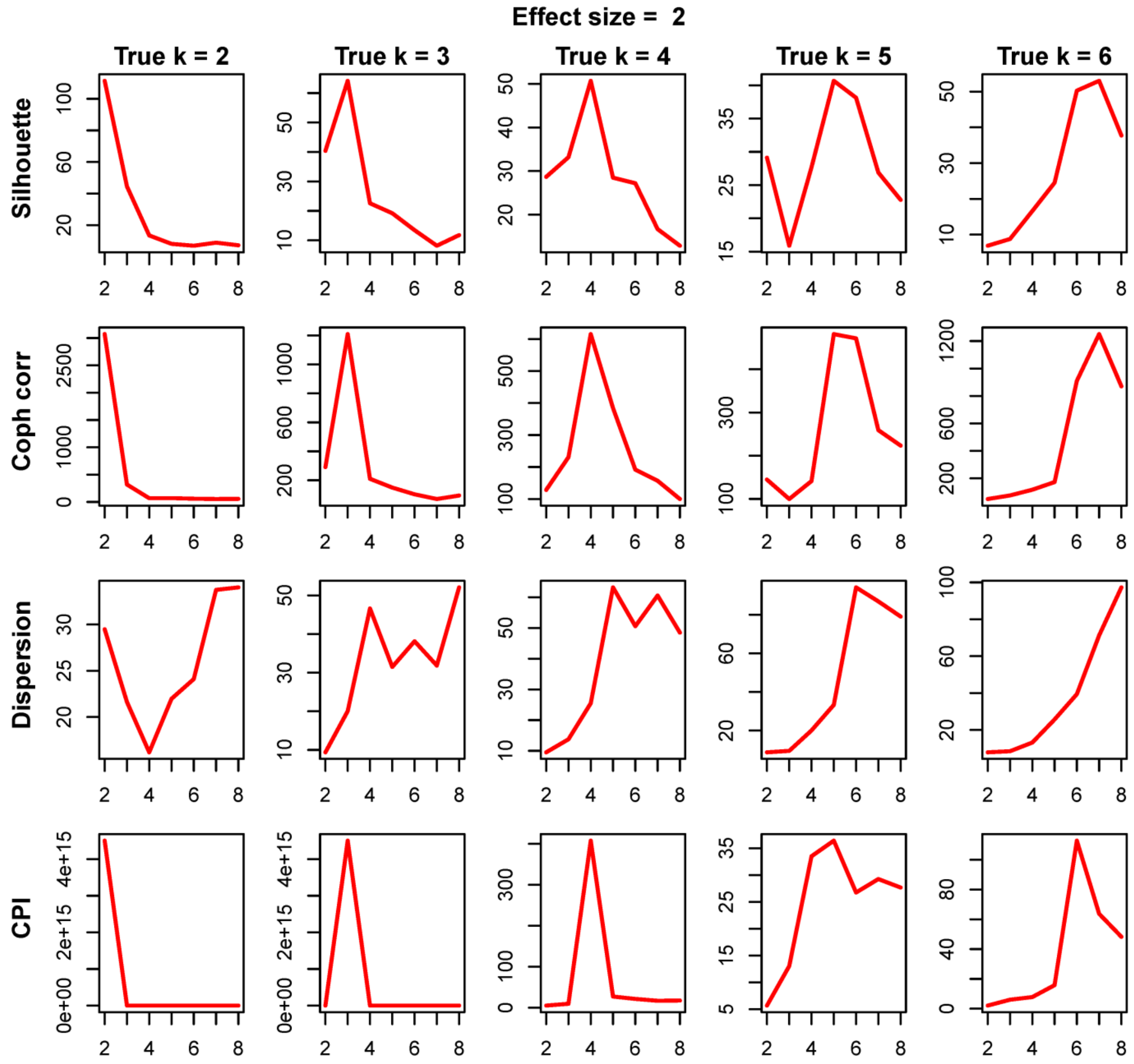

S18 Fig: Signal to noise ratio. Plots showing the signal to noise ratio (mean/sd) for the four parameters for finding optimum number of clusters for cluster mean shift effect size of 2.0 and varying scenarios of true number of clusters.

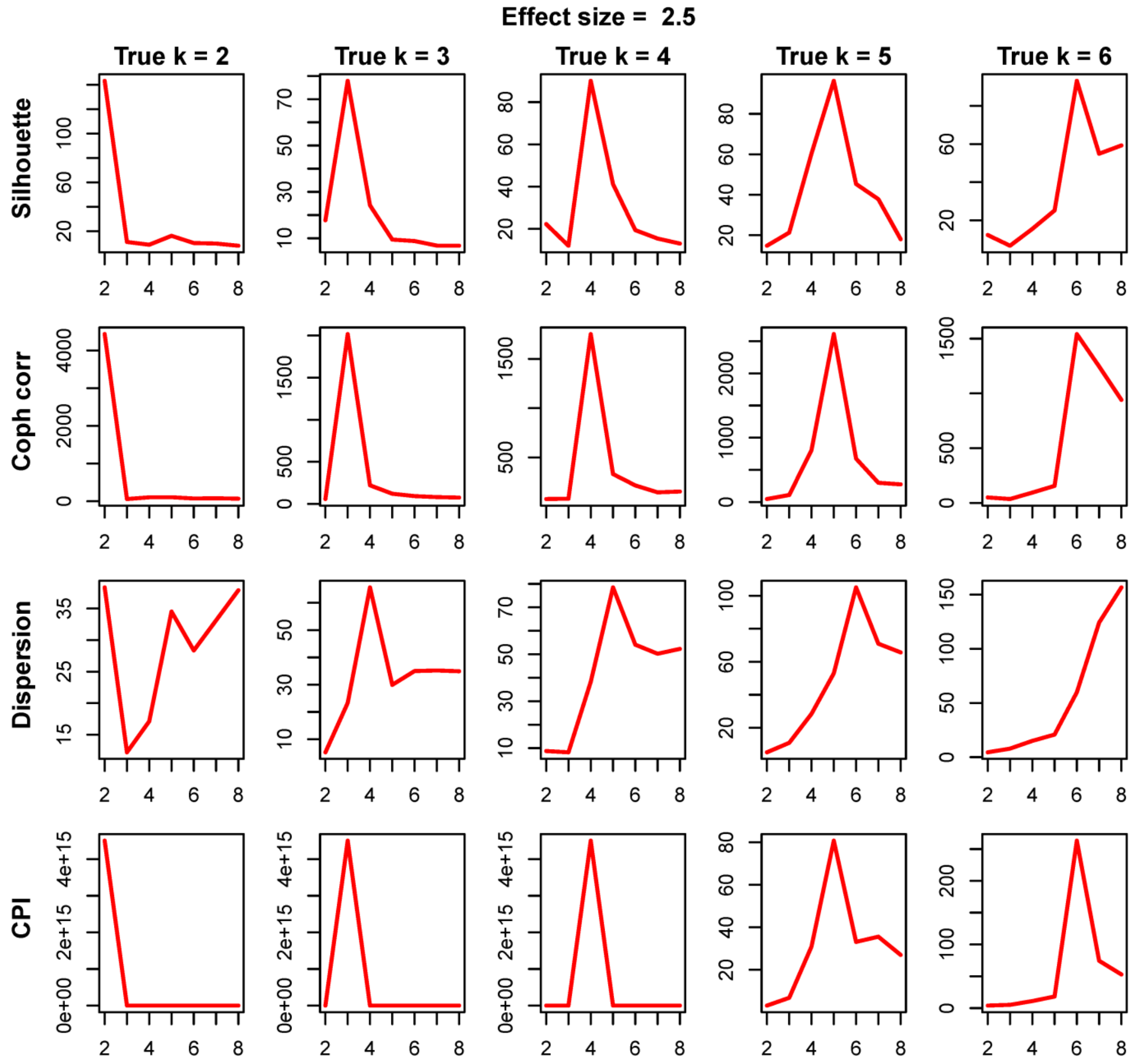

S19 Fig: Signal to noise ratio. Plots showing the signal to noise ratio (mean/sd) for the four parameters for finding optimum number of clusters for cluster mean shift effect size of 2.5 and varying scenarios of true number of clusters.

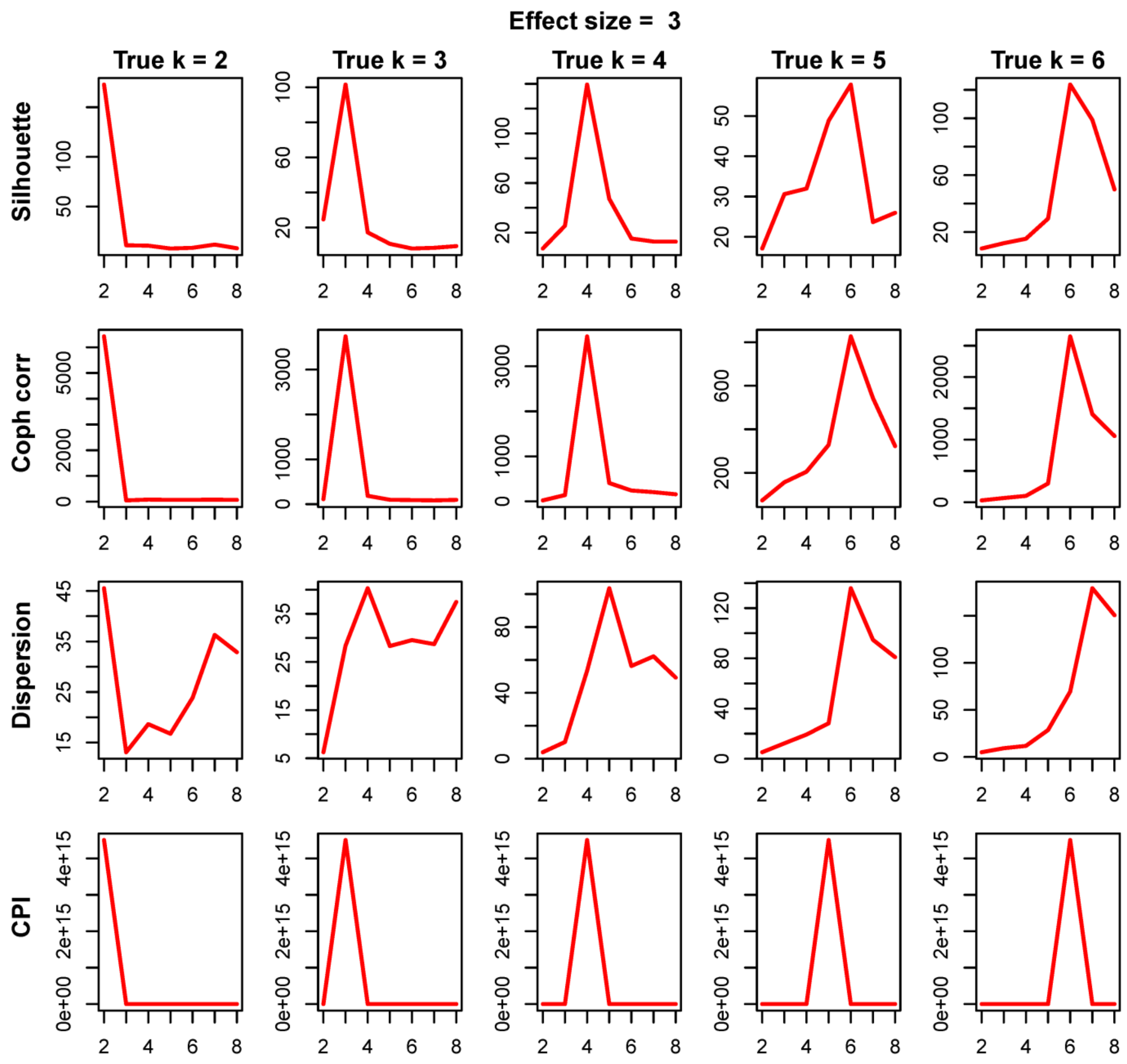

S20 Fig: Signal to noise ratio. Plots showing the signal to noise ratio (mean/sd) for the four parameters for finding optimum number of clusters for cluster mean shift effect size of 3.0 and varying scenarios of true number of clusters.

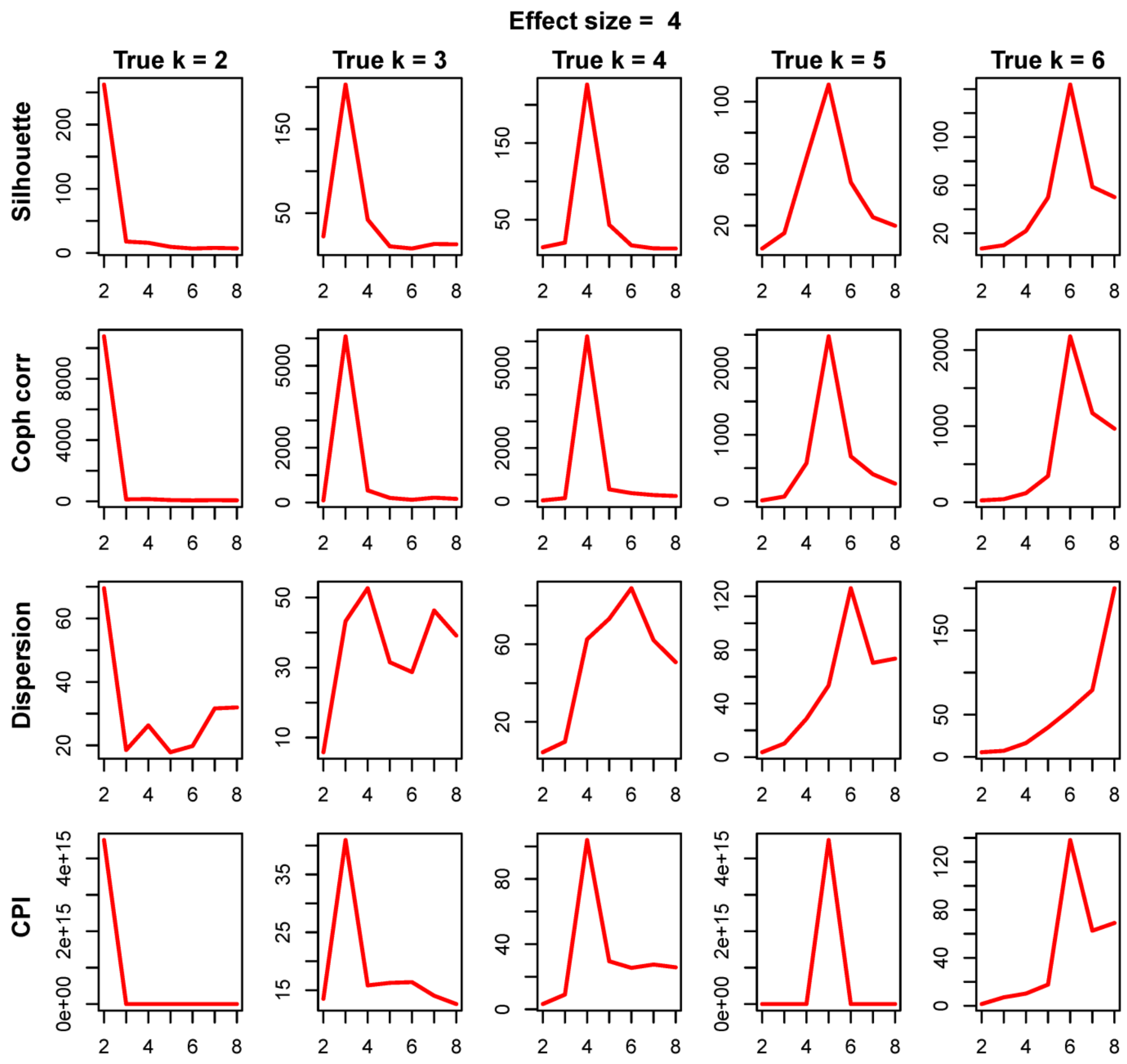

S21 Fig: Signal to noise ratio. Plots showing the signal to noise ratio (mean/sd) for the four parameters for finding optimum number of clusters for cluster mean shift effect size of 4.0 and varying scenarios of true number of clusters.

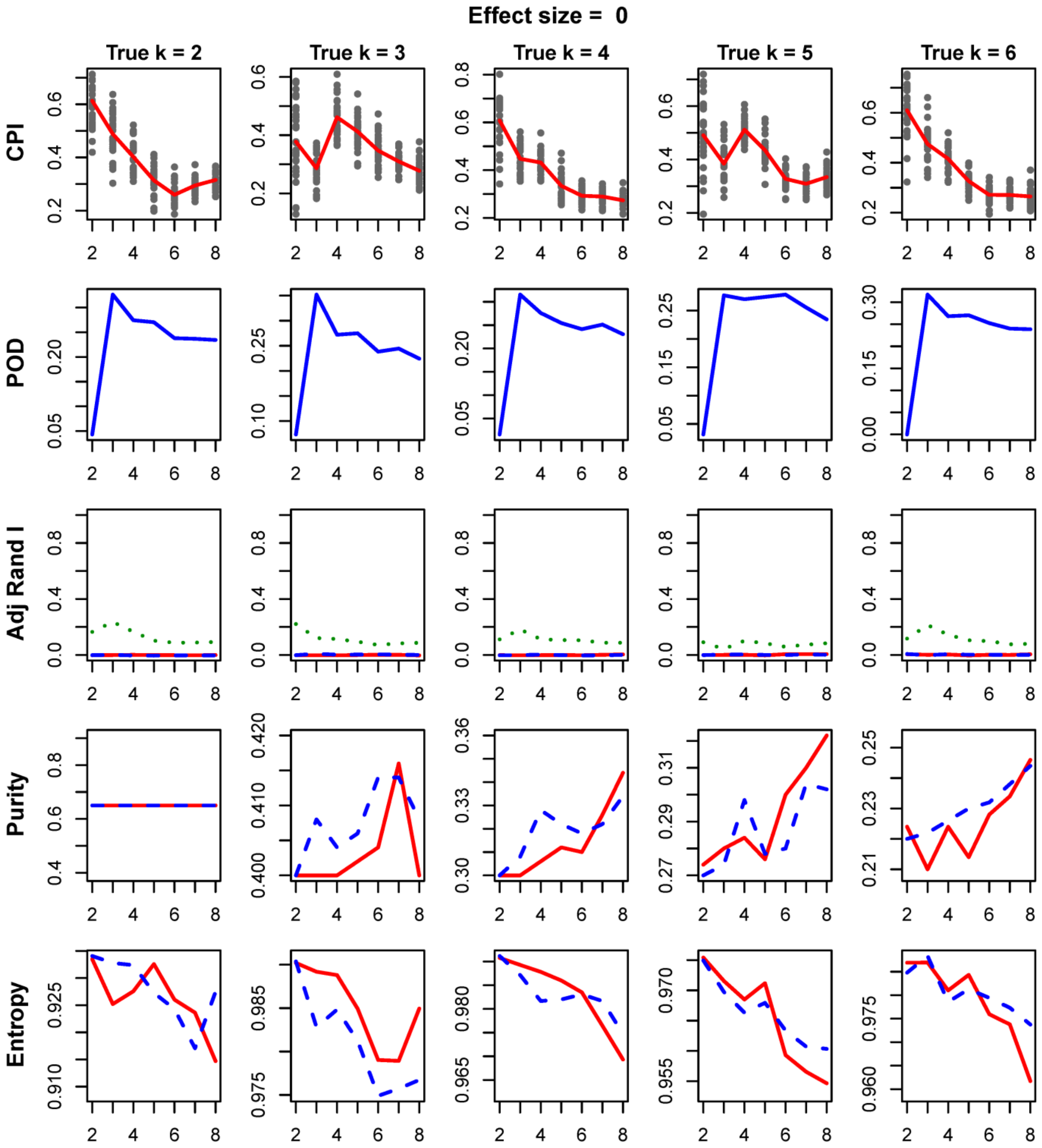

S22 Fig: Comparison of intNMF and iCluster over varying k for effect size 0. First row represents the cluster prediction index, second row represents the plot of proportion of deviance (POD) given by iCluster method and third row represents adjusted rand index between (i) true and intNMF-clusters (red), (ii) true and iCluster-clusters (blue) and (iii) intNMF-clusters and iCluster-clusters (green). Fourth and fifth rows represent the plot of *purity* and *entropy* for intNMF (red) and iCluster (blue).

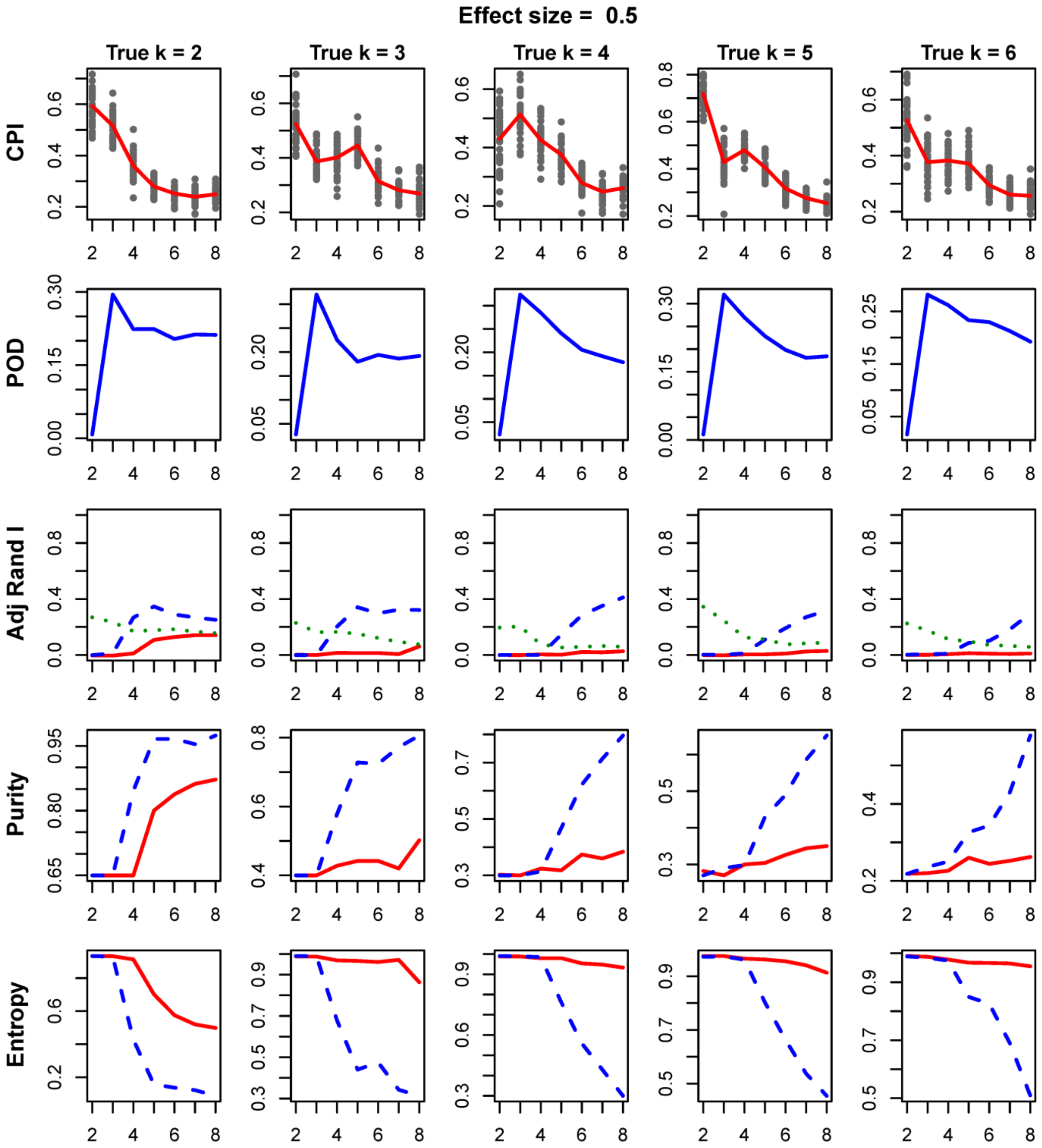

S23 Fig: Comparison of intNMF and iCluster over varying k for effect size 0.5. First row represents the cluster prediction index, second row represents the plot of proportion of deviance (POD) given by iCluster method and third row represents adjusted rand index between (i) true and intNMF-clusters (red), (ii) true and iCluster-clusters (blue) and (iii) intNMF-clusters and iCluster-clusters (green). Fourth and fifth rows represent the plot of *purity* and *entropy* for intNMF (red) and iCluster (blue).

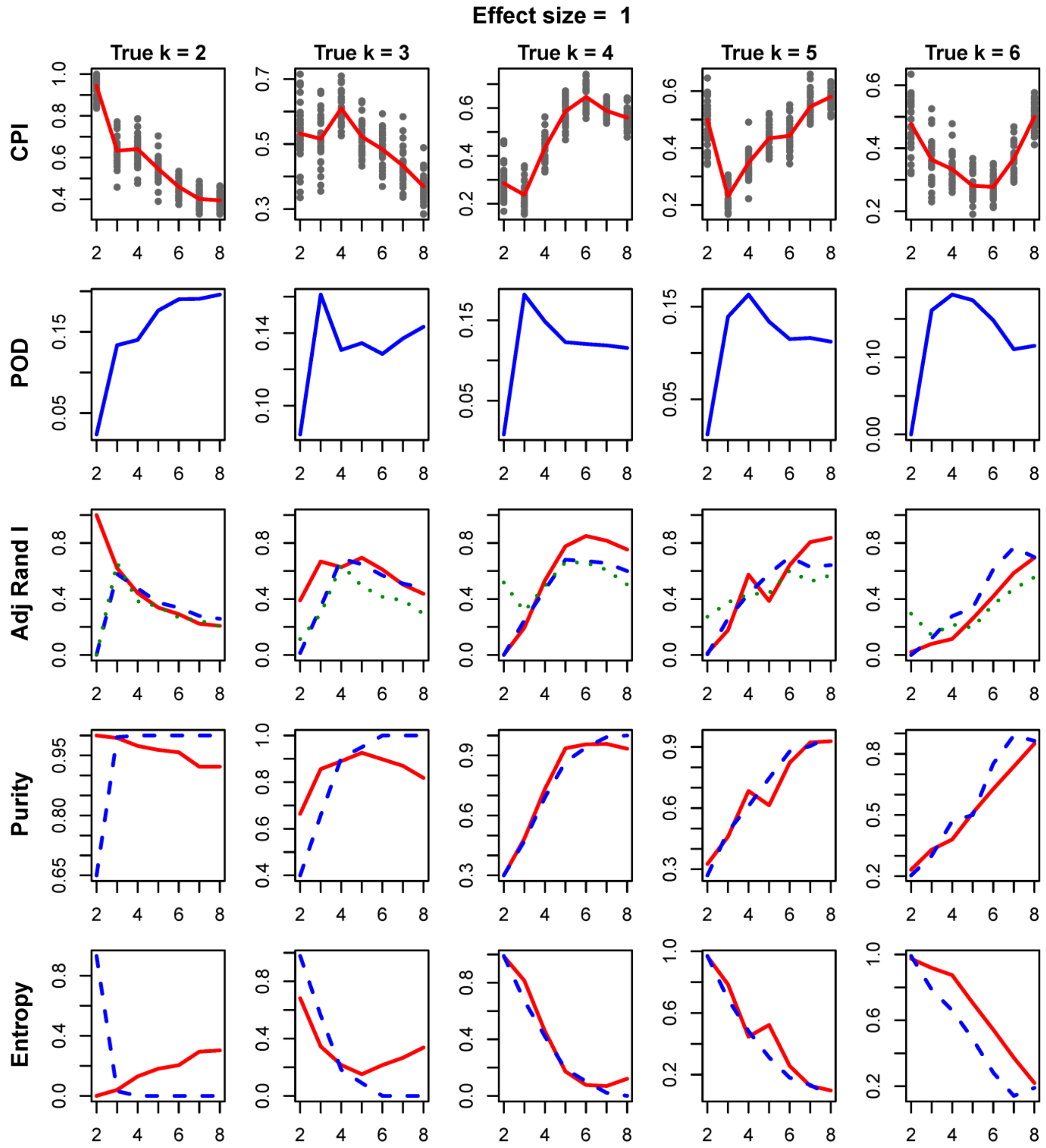

S24 Fig: Comparison of intNMF and iCluster over varying k for effect size 1.0. First row represents the cluster prediction index, second row represents the plot of proportion of deviance (POD) given by iCluster method and third row represents adjusted rand index between (i) true and intNMF-clusters (red), (ii) true and iCluster-clusters (blue) and (iii) intNMF-clusters and iCluster-clusters (green). Fourth and fifth rows represent the plot of *purity* and *entropy* for intNMF (red) and iCluster (blue).

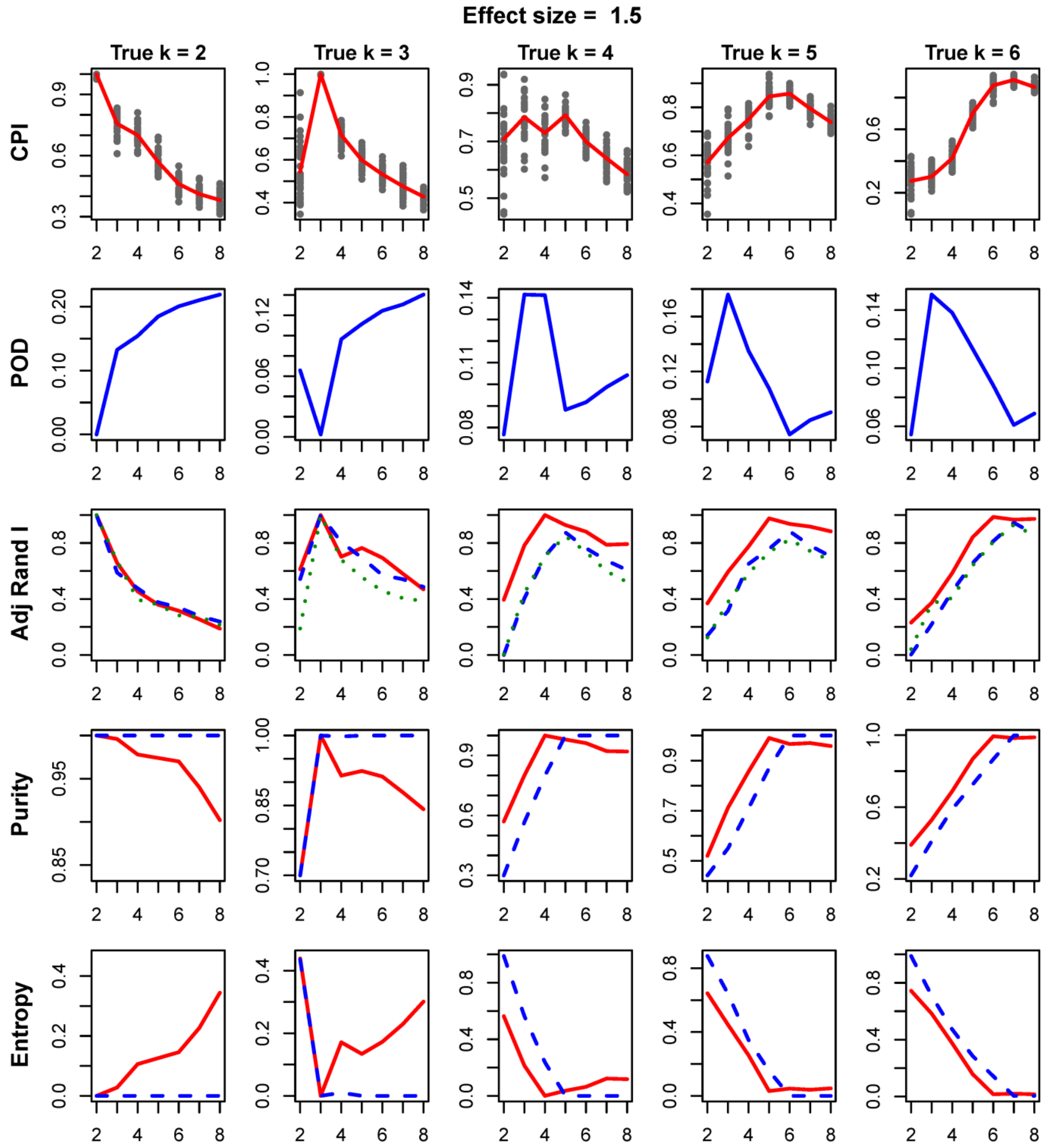

S25 Fig: Comparison of intNMF and iCluster over varying k for effect size 1.5. First row represents the cluster prediction index, second row represents the plot of proportion of deviance (POD) given by iCluster method and third row represents adjusted rand index between (i) true and intNMF-clusters (red), (ii) true and iCluster-clusters (blue) and (iii) intNMF-clusters and iCluster-clusters (green). Fourth and fifth rows represent the plot of *purity* and *entropy* for intNMF (red) and iCluster (blue).

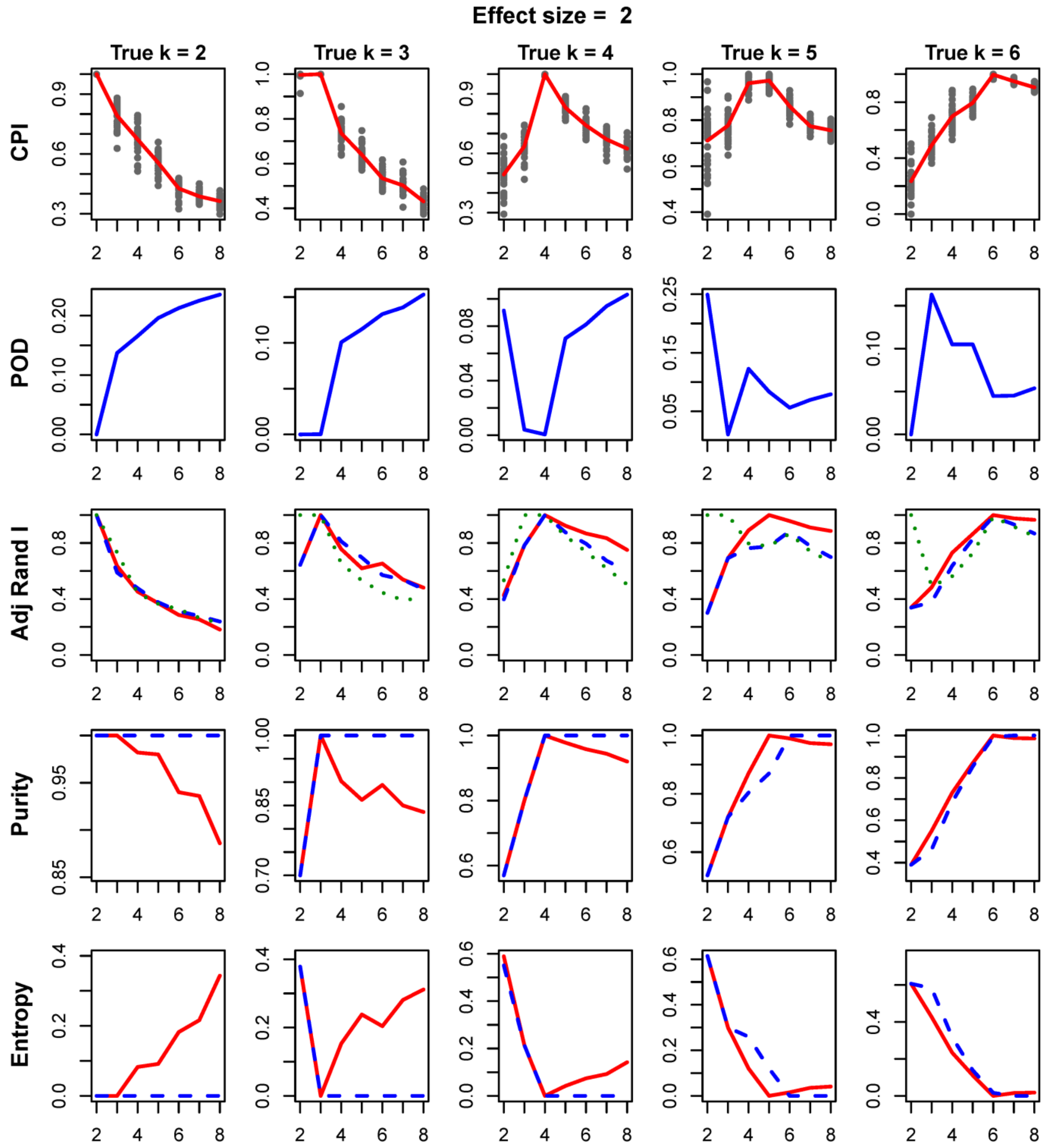

S26 Fig: Comparison of intNMF and iCluster over varying k for effect size 2.0. First row represents the cluster prediction index, second row represents the plot of proportion of deviance (POD) given by iCluster method and third row represents adjusted rand index between (i) true and intNMF-clusters (red), (ii) true and iCluster-clusters (blue) and (iii) intNMF-clusters and iCluster-clusters (green). Fourth and fifth rows represent the plot of *purity* and *entropy* for intNMF (red) and iCluster (blue).

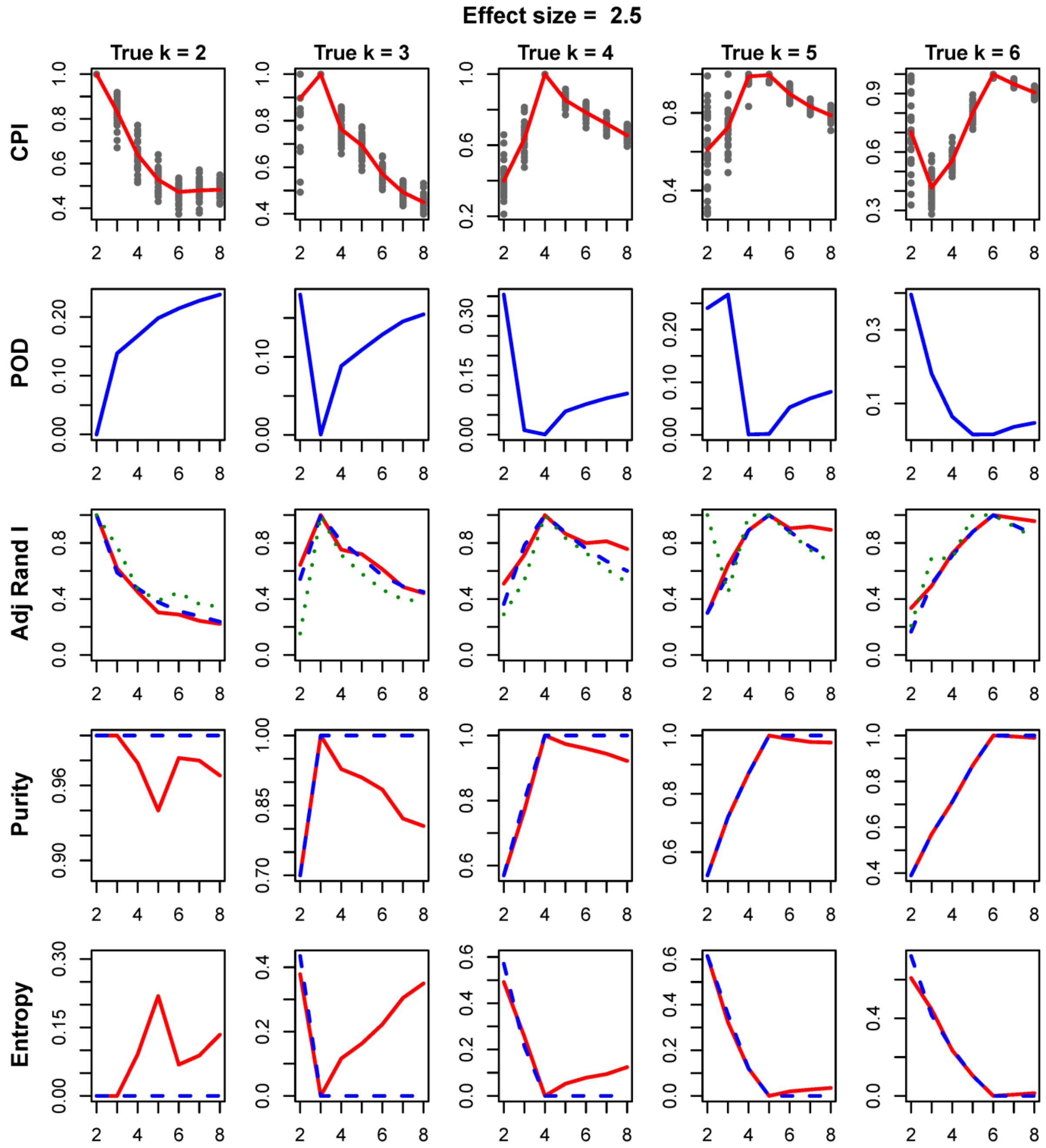

S27 Fig: Comparison of intNMF and iCluster over varying k for effect size 2.5. First row represents the cluster prediction index, second row represents the plot of proportion of deviance (POD) given by iCluster method and third row represents adjusted rand index between (i) true and intNMF-clusters (red), (ii) true and iCluster-clusters (blue) and (iii) intNMF-clusters and iCluster-clusters (green). Fourth and fifth rows represent the plot of *purity* and *entropy* for intNMF (red) and iCluster (blue).

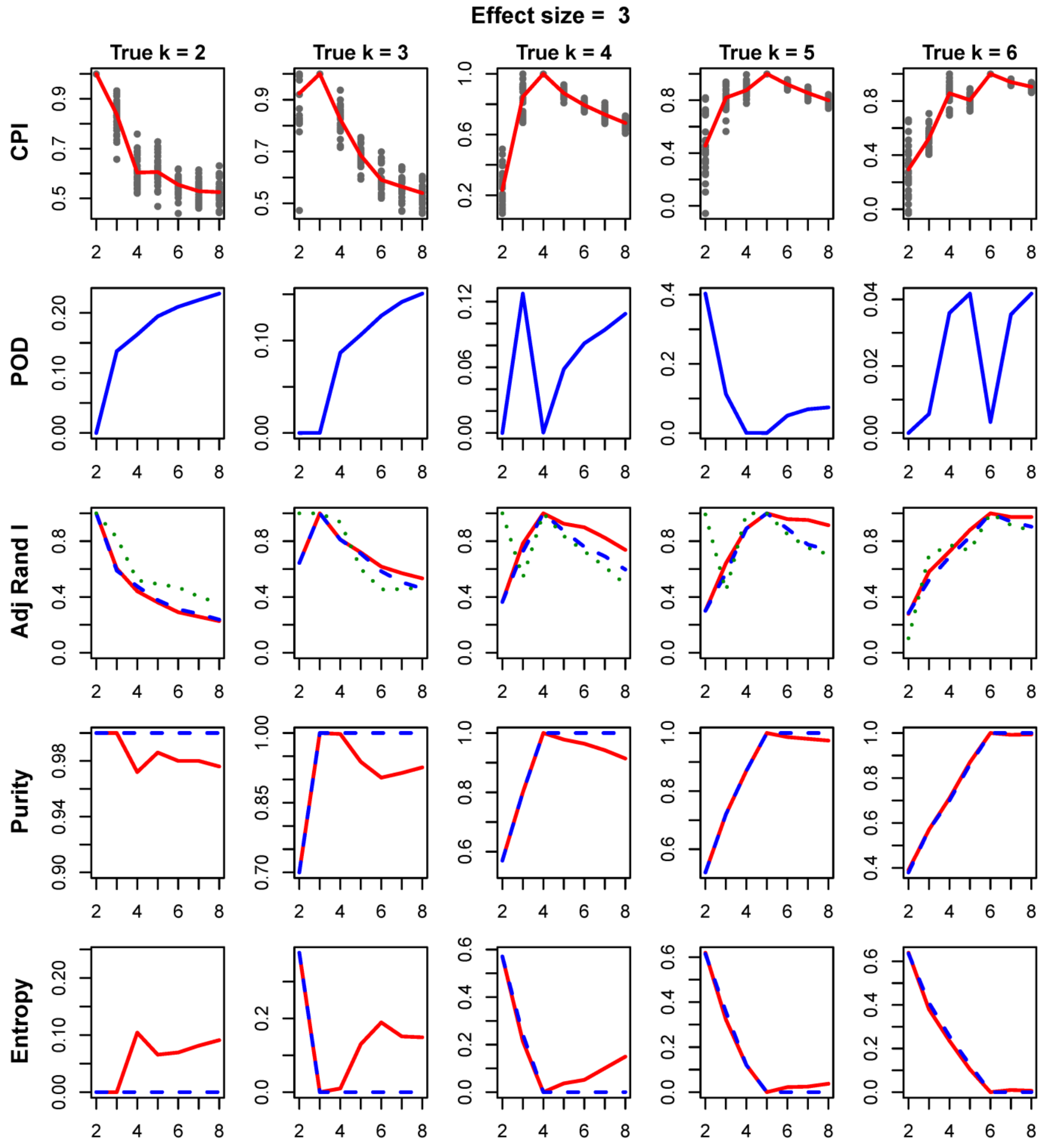

S28 Fig: Comparison of intNMF and iCluster over varying k for effect size 3.0. First row represents the cluster prediction index, second row represents the plot of proportion of deviance (POD) given by iCluster method and third row represents adjusted rand index between (i) true and intNMF-clusters (red), (ii) true and iCluster-clusters (blue) and (iii) intNMF-clusters and iCluster-clusters (green). Fourth and fifth rows represent the plot of *purity* and *entropy* for intNMF (red) and iCluster (blue).

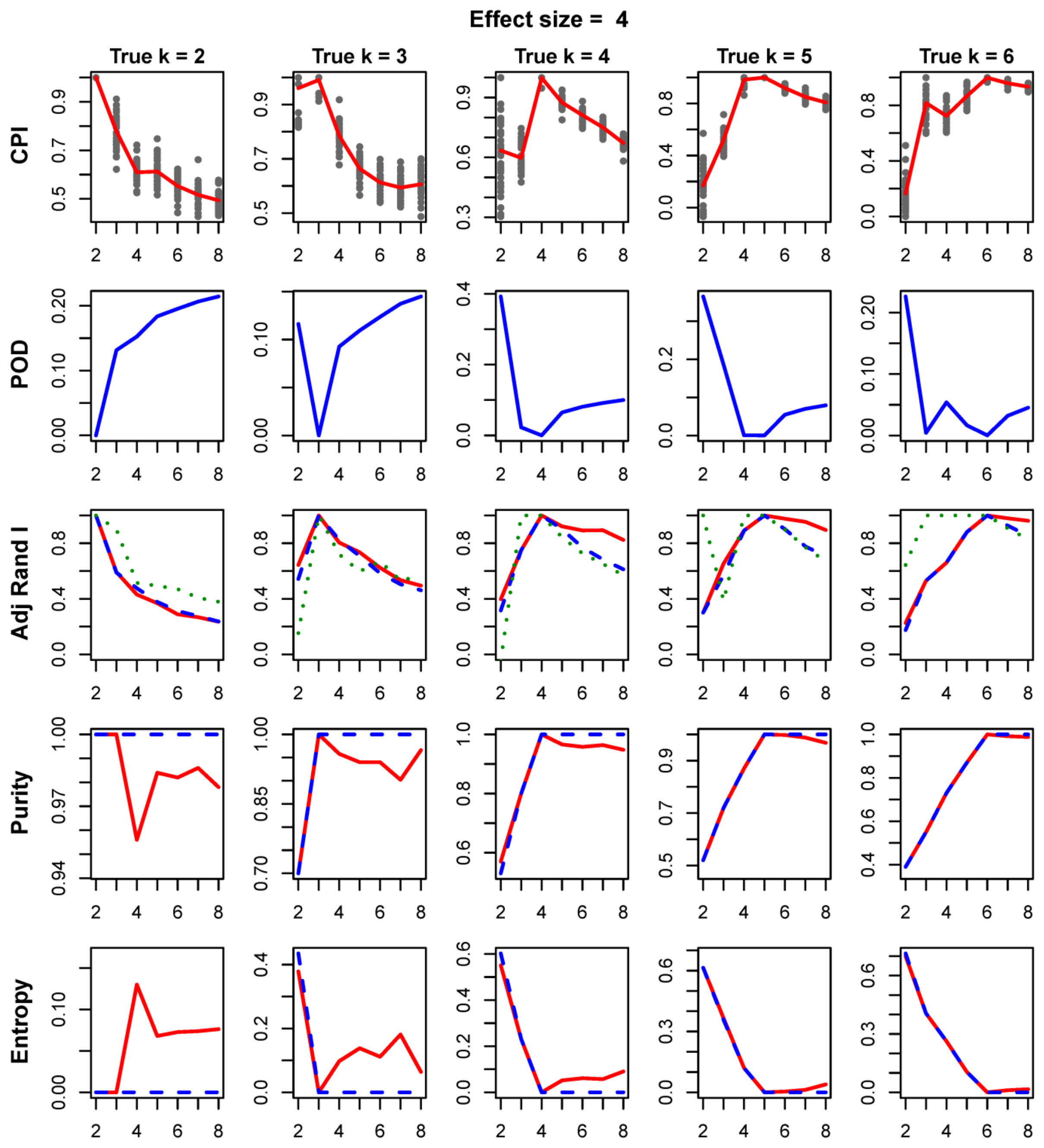

S29 Fig: Comparison of intNMF and iCluster over varying k for effect size 4.0. First row represents the cluster prediction index, second row represents the plot of proportion of deviance (POD) given by iCluster method and third row represents adjusted rand index between (i) true and intNMF-clusters (red), (ii) true and iCluster-clusters (blue) and (iii) intNMF-clusters and iCluster-clusters (green). Fourth and fifth rows represent the plot of *purity* and *entropy* for intNMF (red) and iCluster (blue).

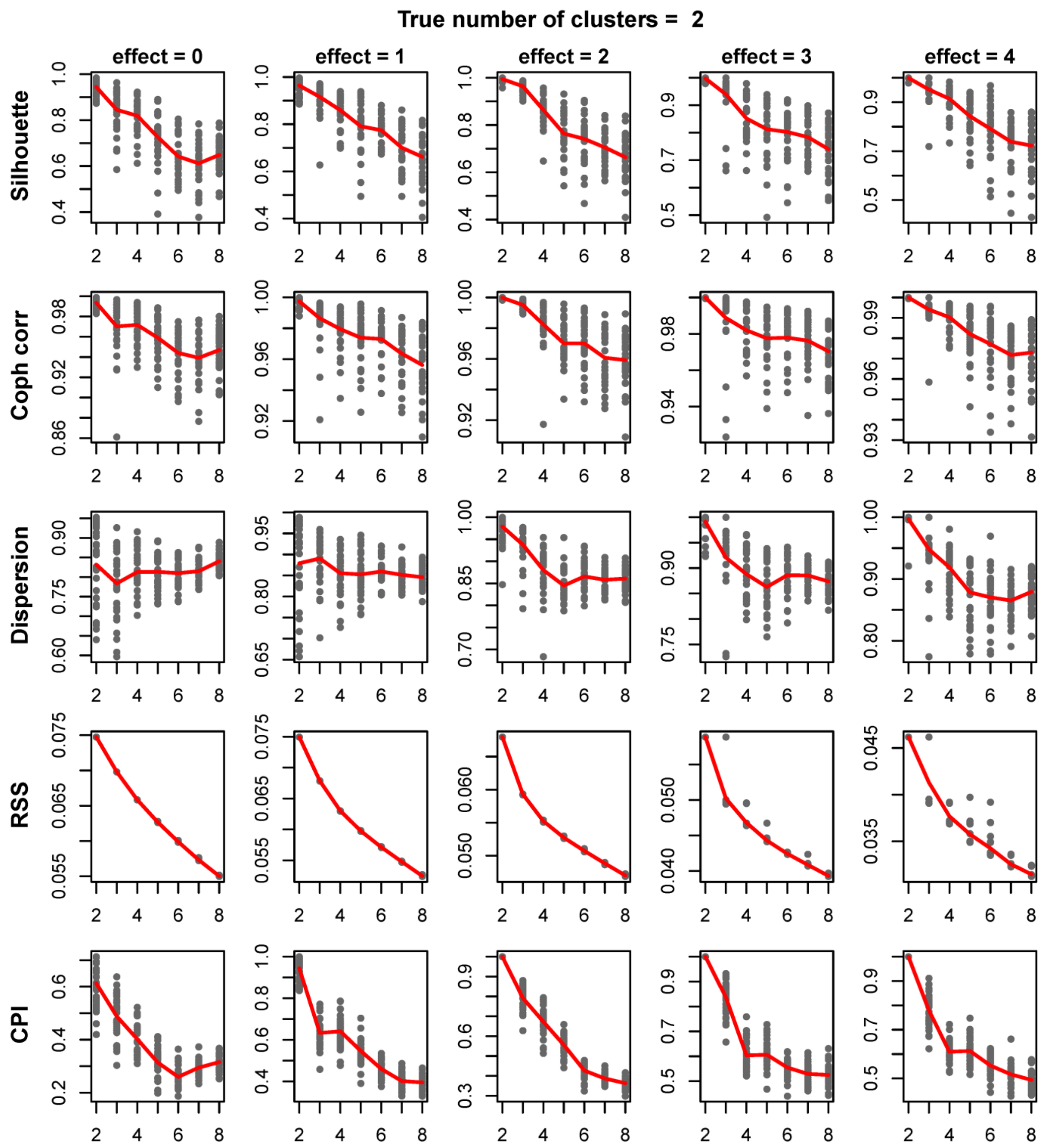

S30 Fig: Finding Optimum number of clusters. Plots showing the comparison of five different methods of finding optimum number of clusters on the dataset generated using varying effect sizes for true number of clusters k = 2. The average values of the parameters over 30 runs are overlaid on the plots as a line.

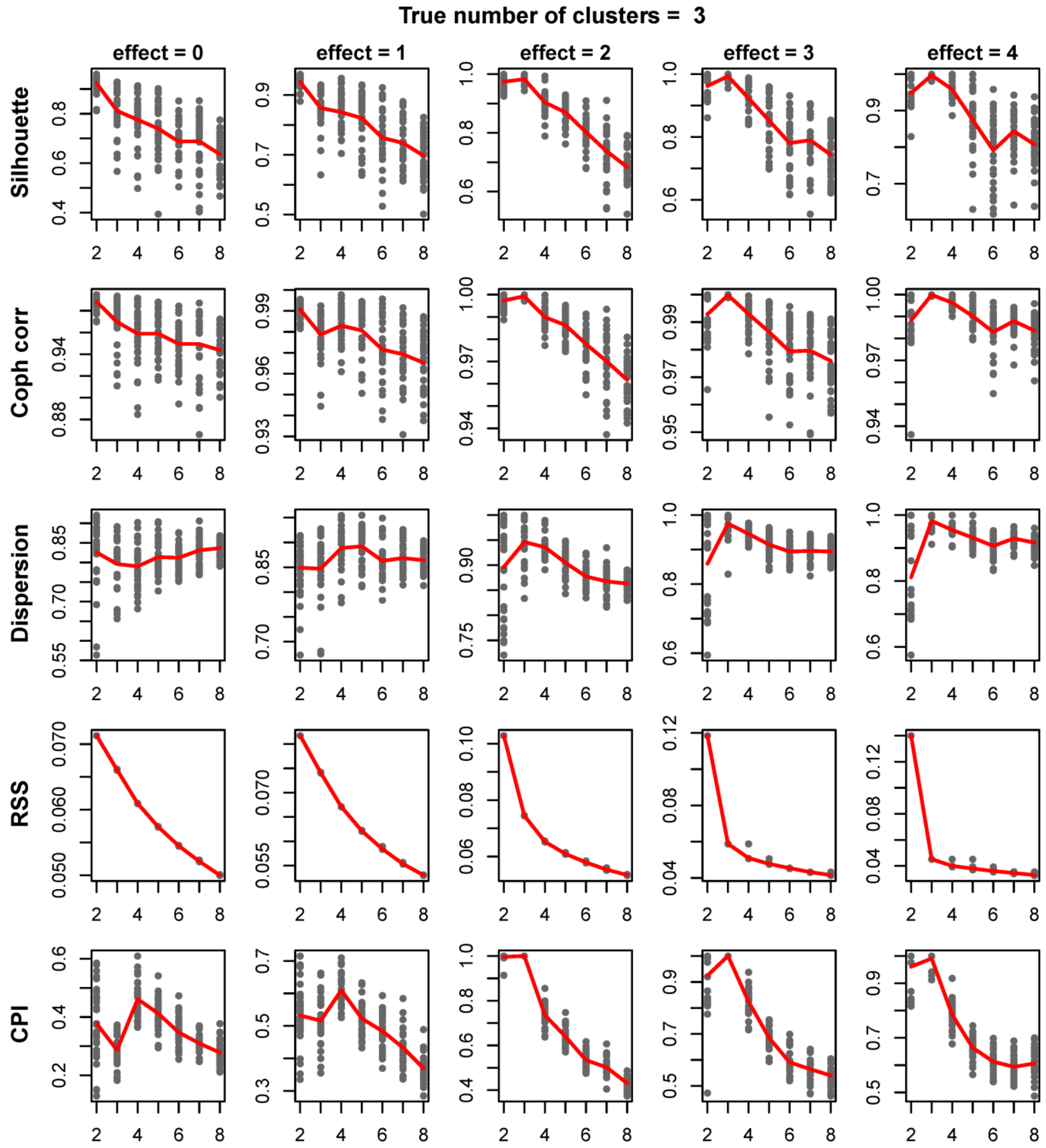

S31 Fig: Finding Optimum number of clusters. Plots showing the comparison of five different methods of finding optimum number of clusters on the dataset generated using varying effect sizes for true number of clusters k = 3. The average values of the parameters over 30 runs are overlaid on the plots as a line.

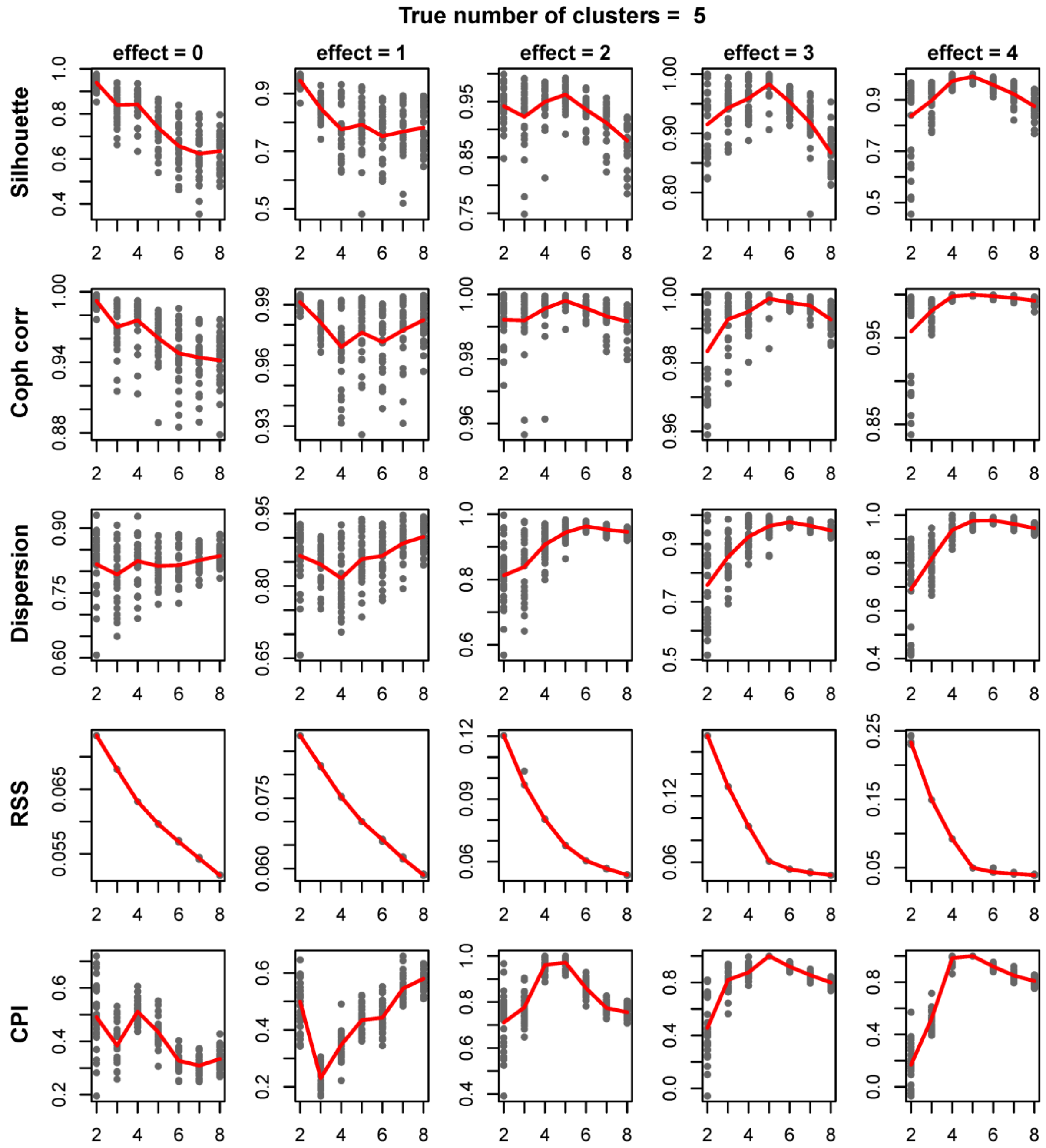

S32 Fig: Finding Optimum number of clusters. Plots showing the comparison of five different methods of finding optimum number of clusters on the dataset generated using varying effect sizes for true number of clusters k = 5. The average values of the parameters over 30 runs are overlaid on the plots as a line.

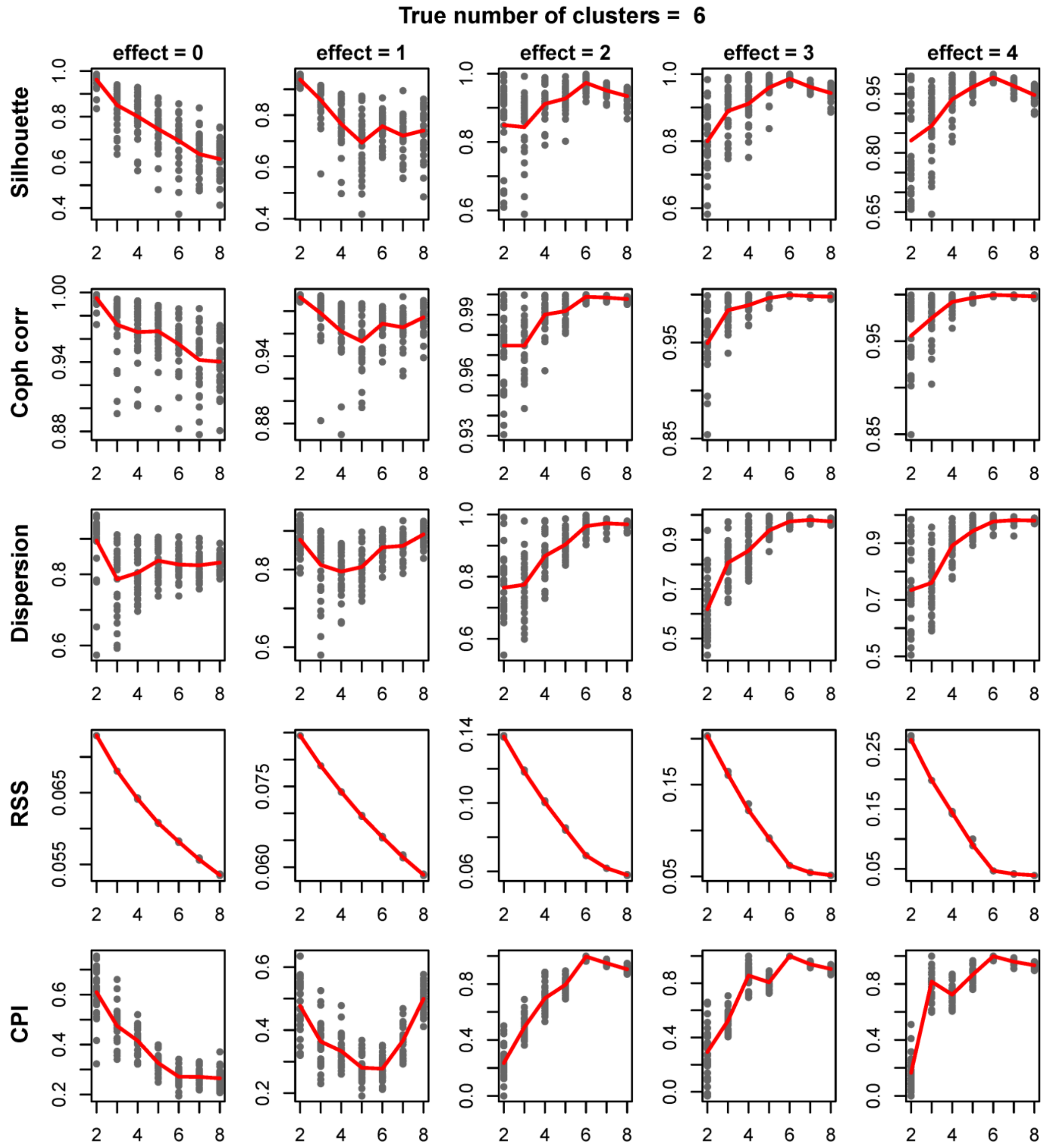

S33 Fig: Finding Optimum number of clusters. Plots showing the comparison of five different methods of finding optimum number of clusters on the dataset generated using varying effect sizes for true number of clusters k = 6. The average values of the parameters over 30 runs are overlaid on the plots as a line.

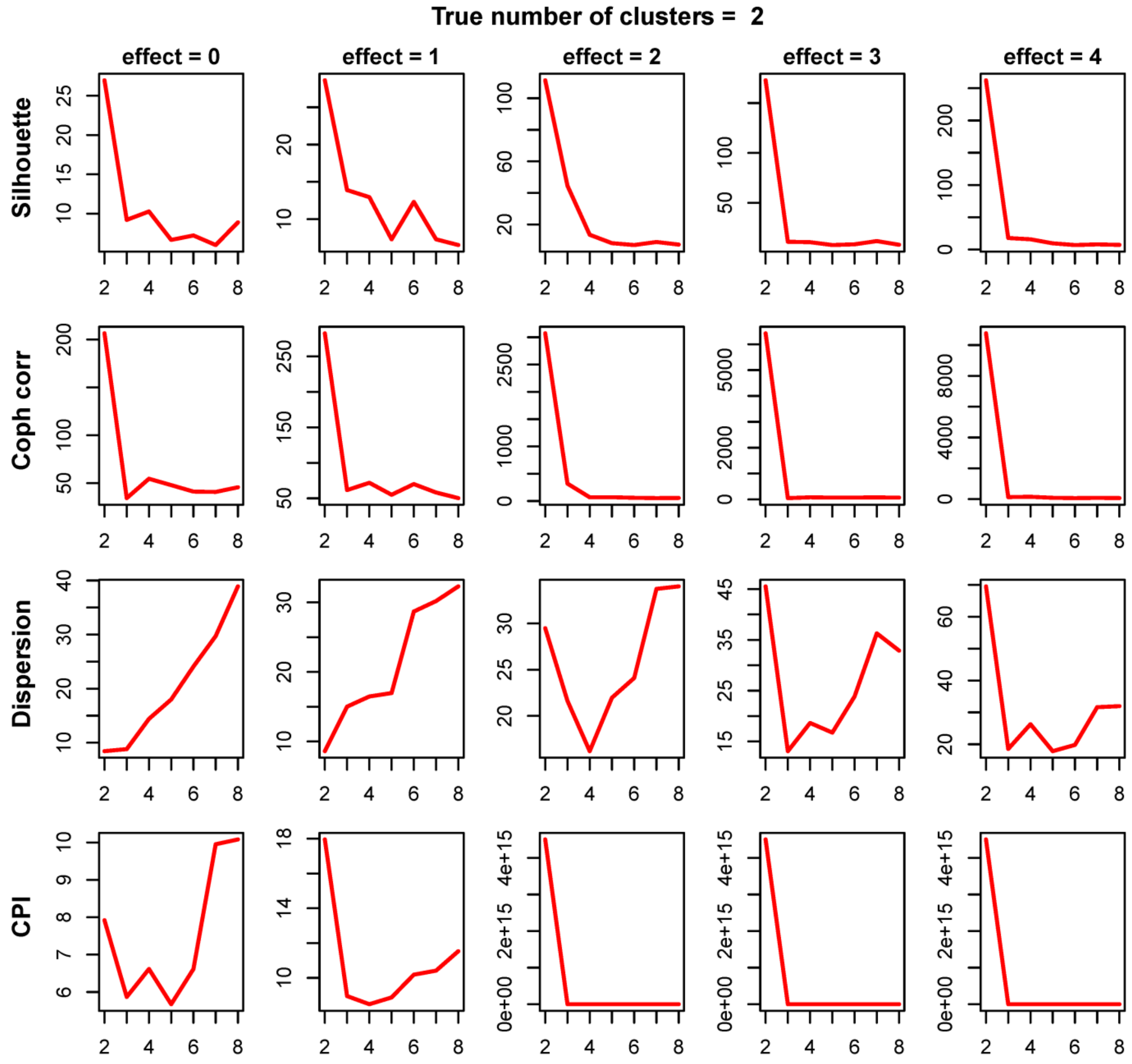

S34 Fig: Signal to noise ratio. Plots showing the signal to noise ratio (mean/sd) for the four parameters for true number of clusters k=2 with varying effect sizes.

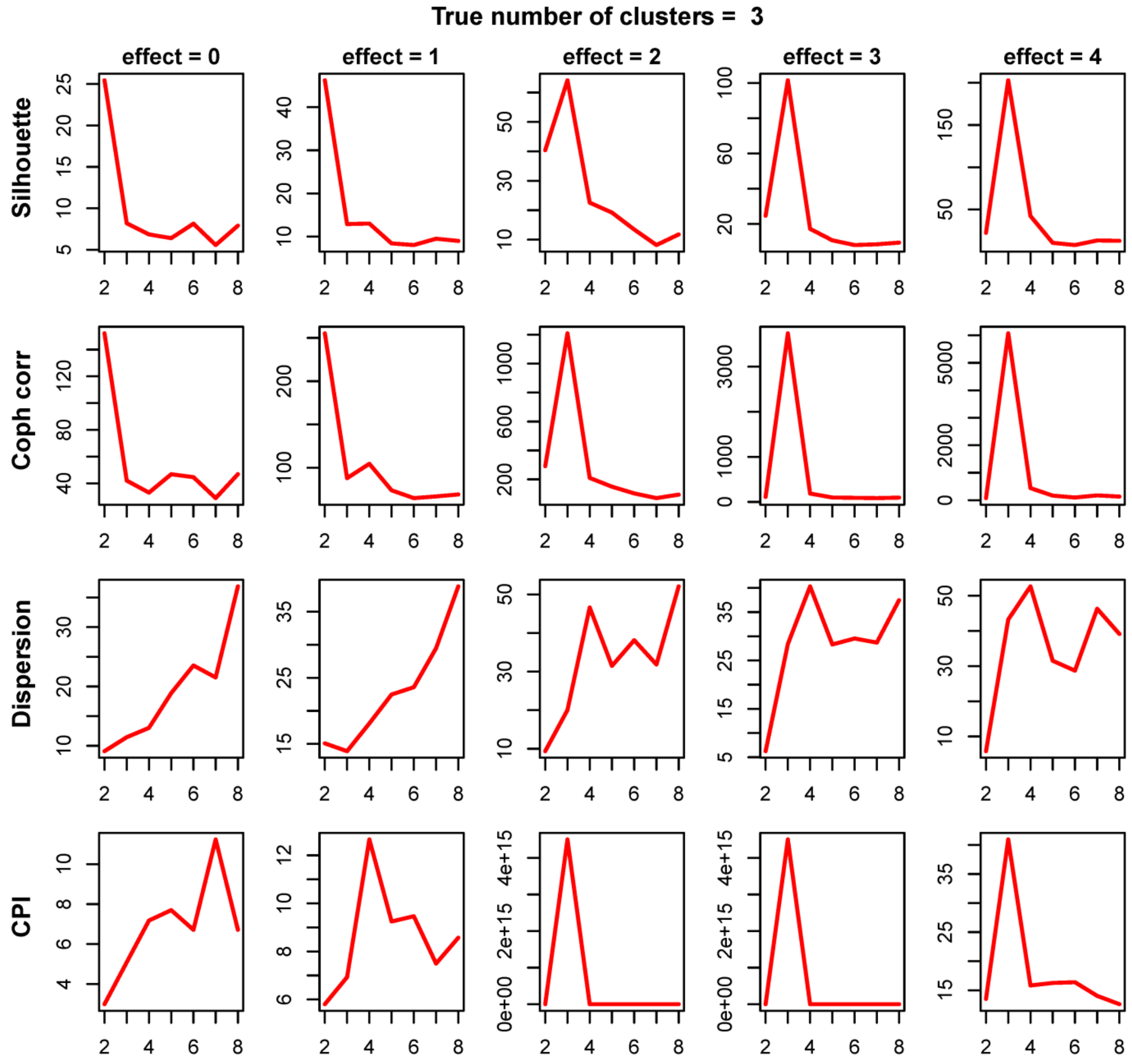

S35 Fig: Signal to noise ratio. Plots showing the signal to noise ratio (mean/sd) for the four parameters for true number of clusters k=3 with varying effect sizes.

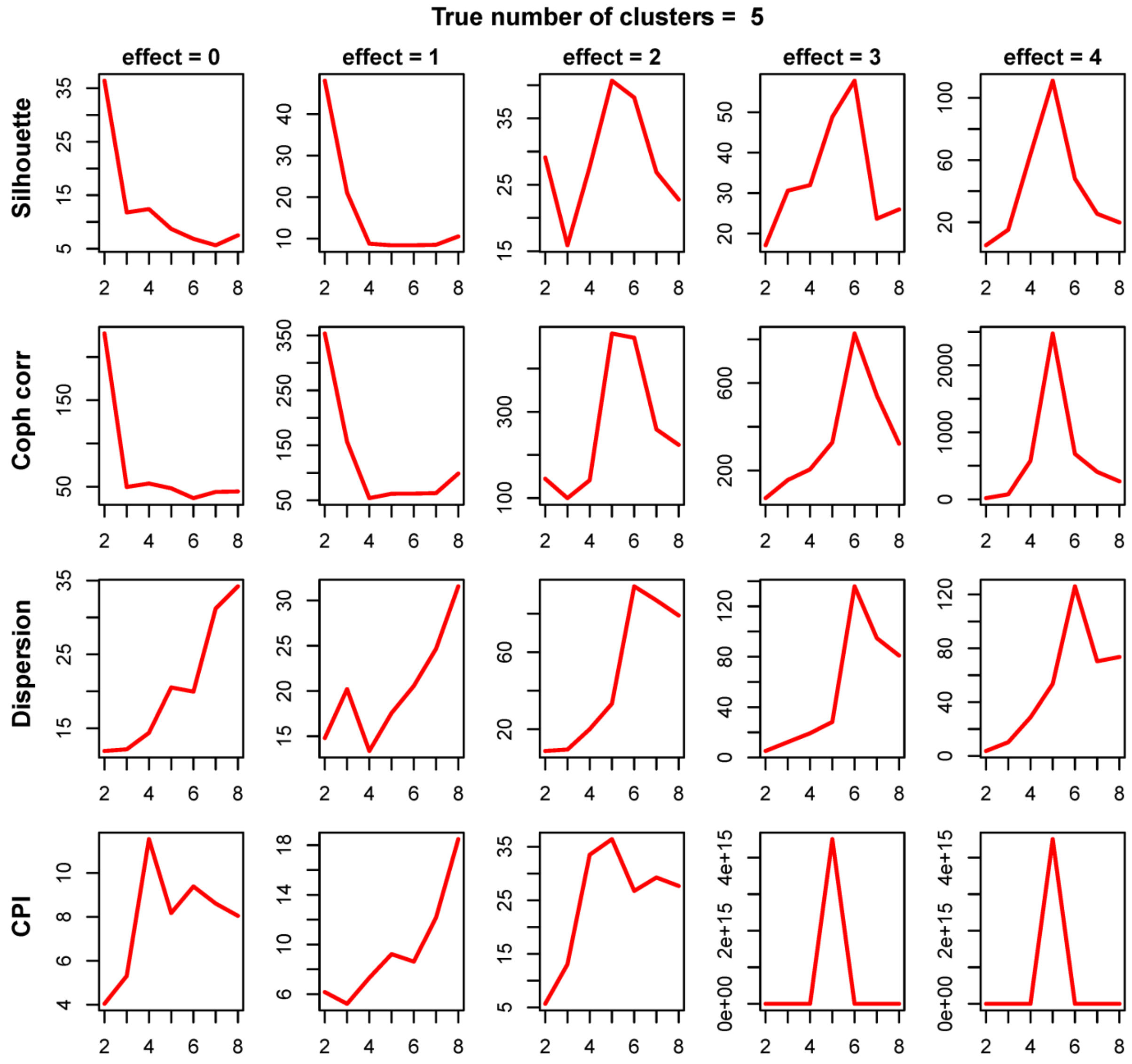

S36 Fig: Signal to noise ratio. Plots showing the signal to noise ratio (mean/sd) for the four parameters for true number of clusters k=5 with varying effect sizes.

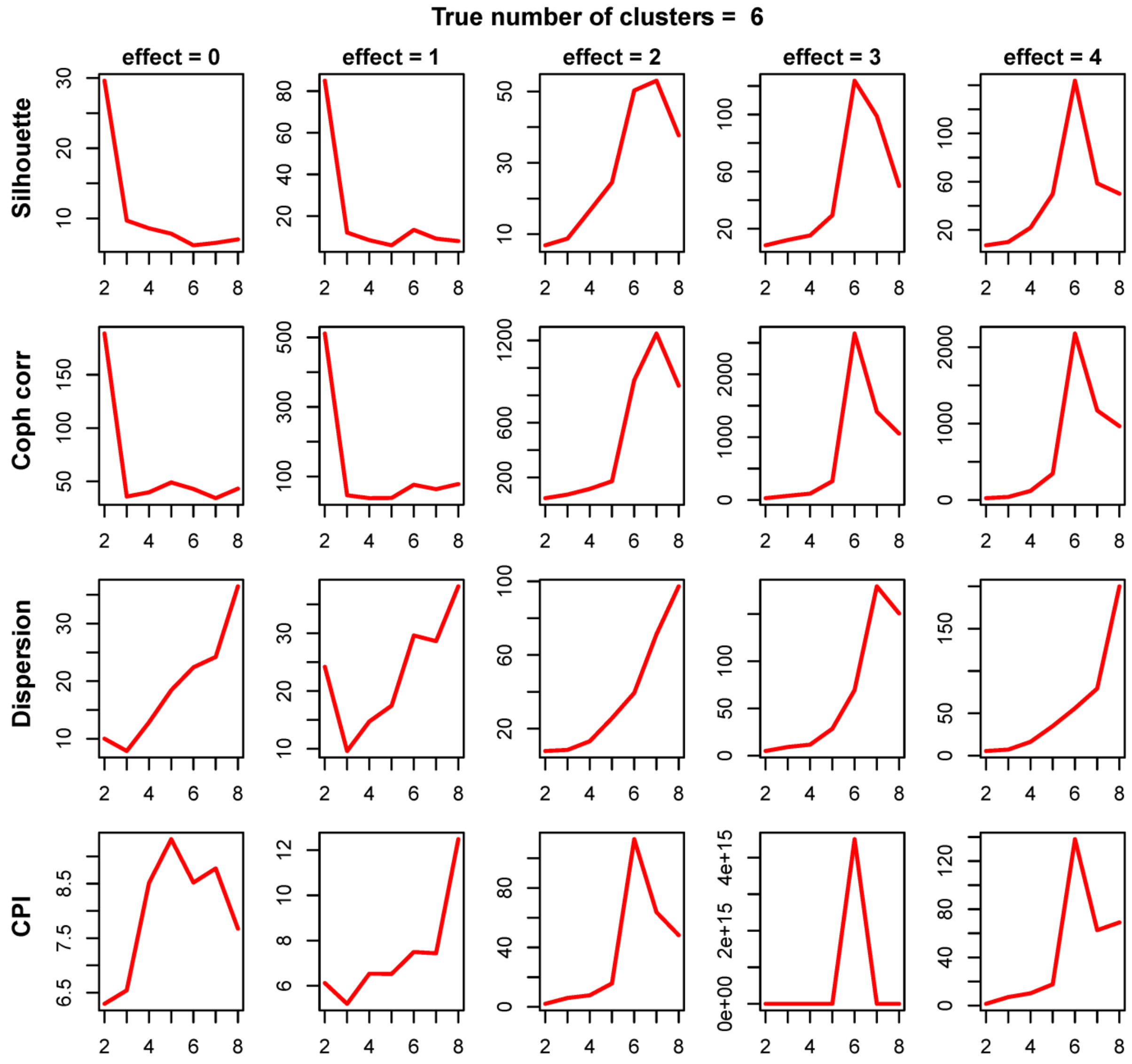

S37 Fig: Signal to noise ratio. Plots showing the signal to noise ratio (mean/sd) for the four parameters for true number of clusters k=6 with varying effect sizes.

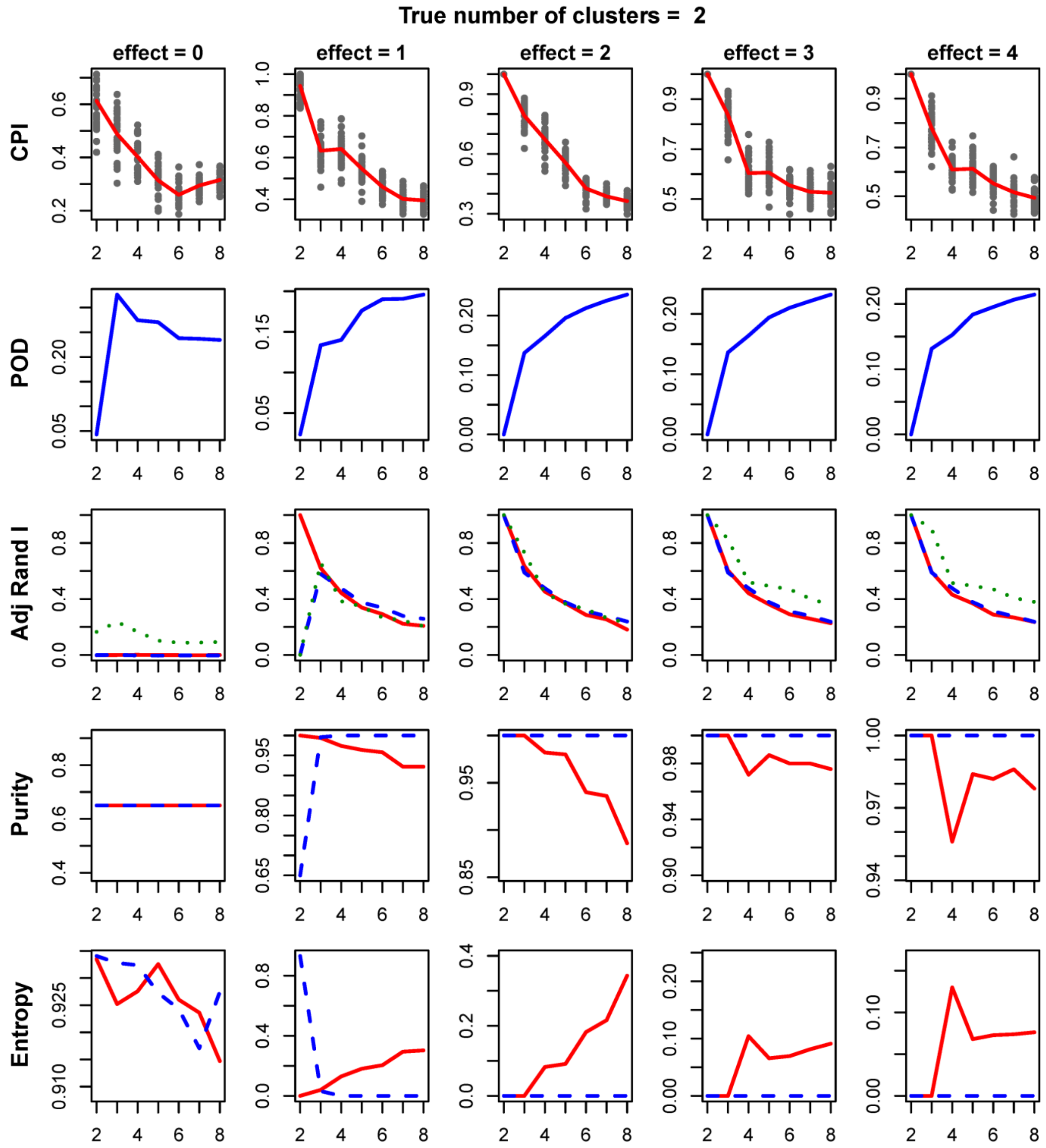

S38 Fig: Comparison of intNMF and iCluster over varying effect sizes and k=2. First row represents the cluster prediction index, second row represents the plot of proportion of deviance (POD) given by iCluster method and third row represents adjusted rand index between (i) true and intNMF-clusters (red), (ii) true and iCluster-clusters (blue) and (iii) intNMF-clusters and iCluster-clusters (green). Fourth and fifth rows represent the plot of *purity* and *entropy* for intNMF (red) and iCluster (blue).

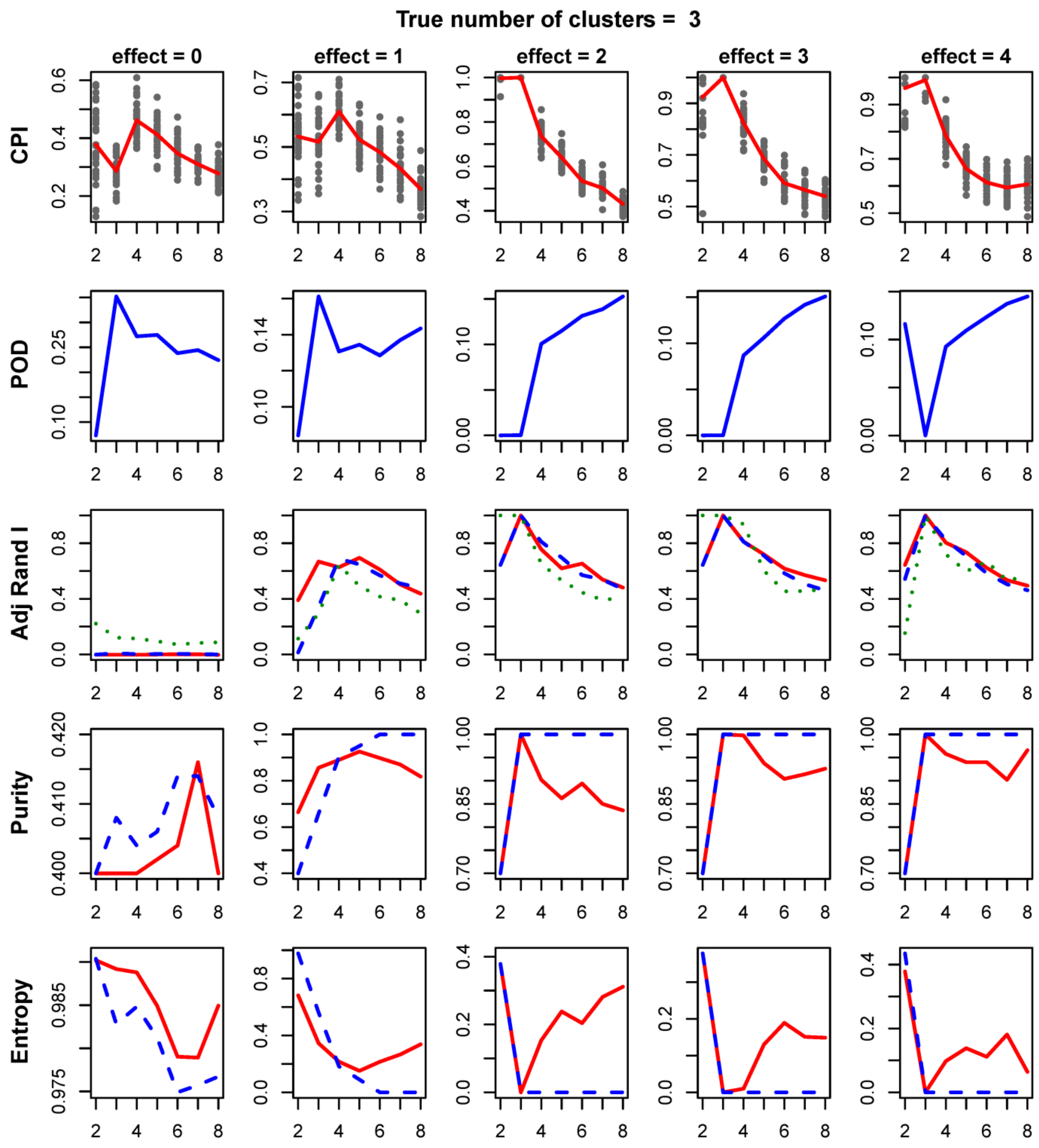

S39 Fig: Comparison of intNMF and iCluster over varying effect sizes and k=3. First row represents the cluster prediction index, second row represents the plot of proportion of deviance (POD) given by iCluster method and third row represents adjusted rand index between (i) true and intNMF-clusters (red), (ii) true and iCluster-clusters (blue) and (iii) intNMF-clusters and iCluster-clusters (green). Fourth and fifth rows represent the plot of *purity* and *entropy* for intNMF (red) and iCluster (blue).

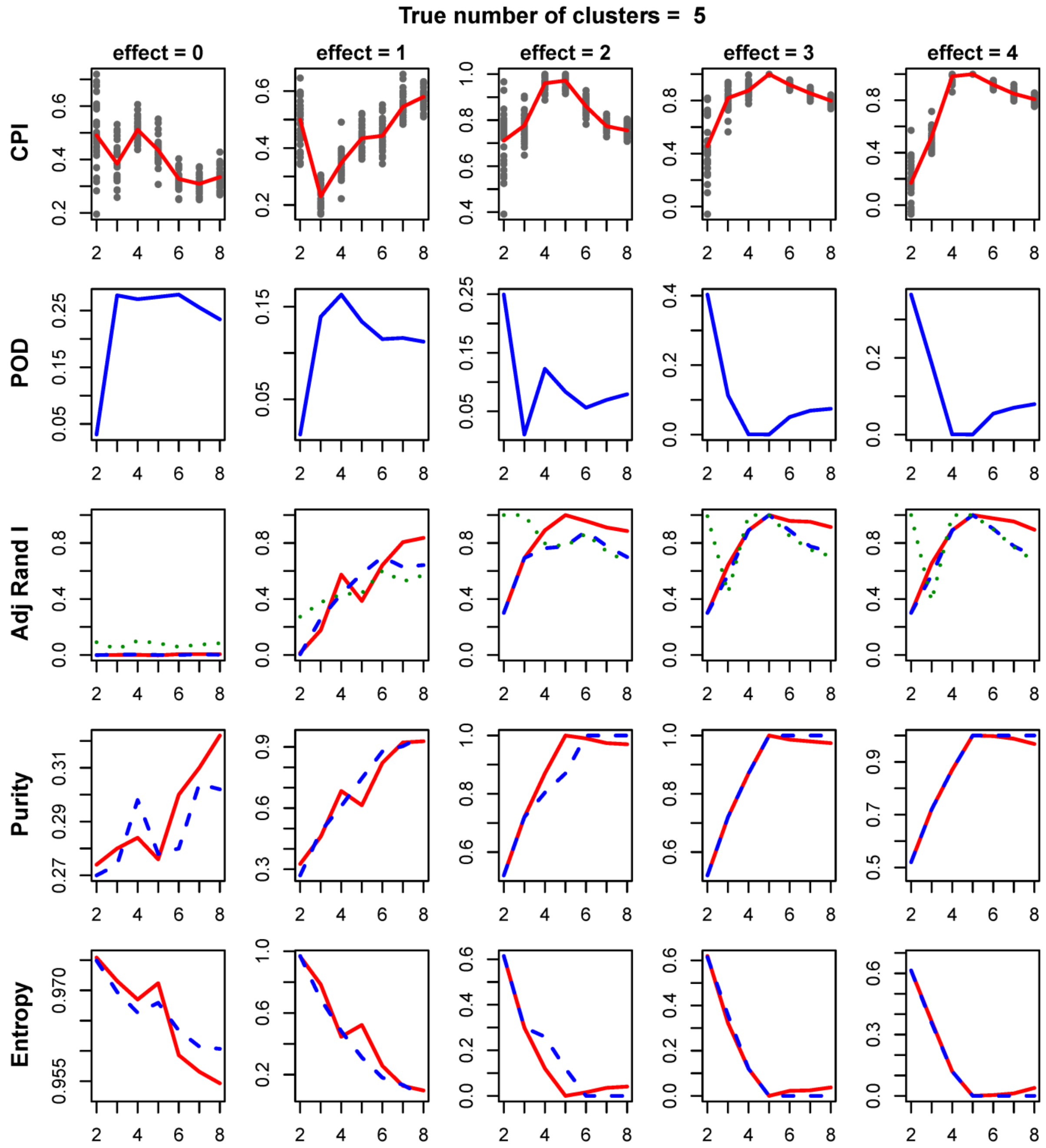

S40 Fig: Comparison of intNMF and iCluster over varying effect sizes and k=5. First row represents the cluster prediction index, second row represents the plot of proportion of deviance (POD) given by iCluster method and third row represents adjusted rand index between (i) true and intNMF-clusters (red), (ii) true and iCluster-clusters (blue) and (iii) intNMF-clusters and iCluster-clusters (green). Fourth and fifth rows represent the plot of *purity* and *entropy* for intNMF (red) and iCluster (blue).

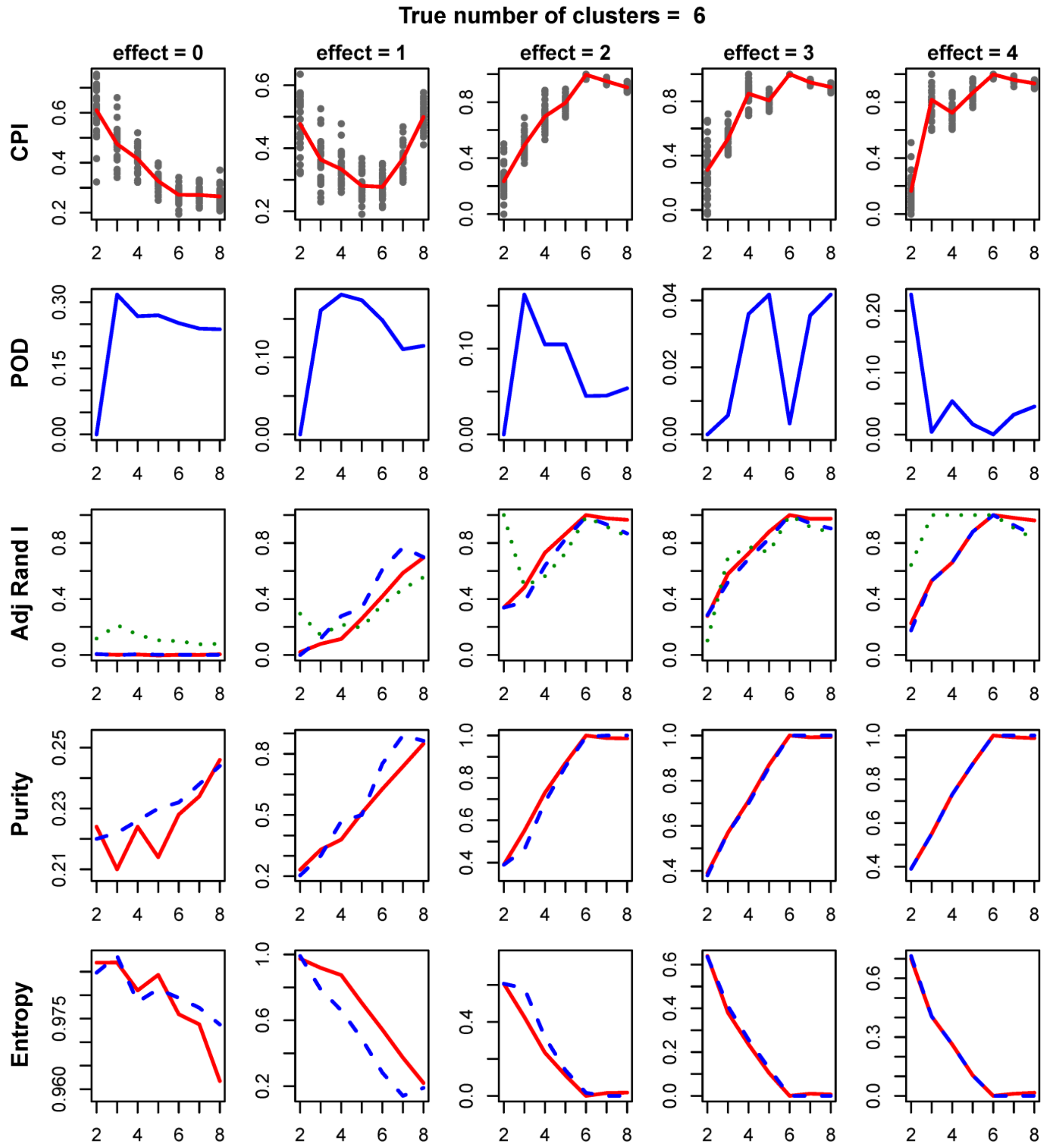

S41 Fig: Comparison of intNMF and iCluster over varying effect sizes and k=6. First row represents the cluster prediction index, second row represents the plot of proportion of deviance (POD) given by iCluster method and third row represents adjusted rand index between (i) true and intNMF-clusters (red), (ii) true and iCluster-clusters (blue) and (iii) intNMF-clusters and iCluster-clusters (green). Fourth and fifth rows represent the plot of *purity* and *entropy* for intNMF (red) and iCluster (blue).
