# Supplementary figures and images for: Integrative clustering of multi-level ‘omic data based on non-negative matrix factorization algorithm
Source: PLoS One. 2017 May 1;12(5):e0176278. doi: 10.1371/journal.pone.0176278 (PMC5411077; doi:10.1371/journal.pone.0176278)

True number of clusters = 4

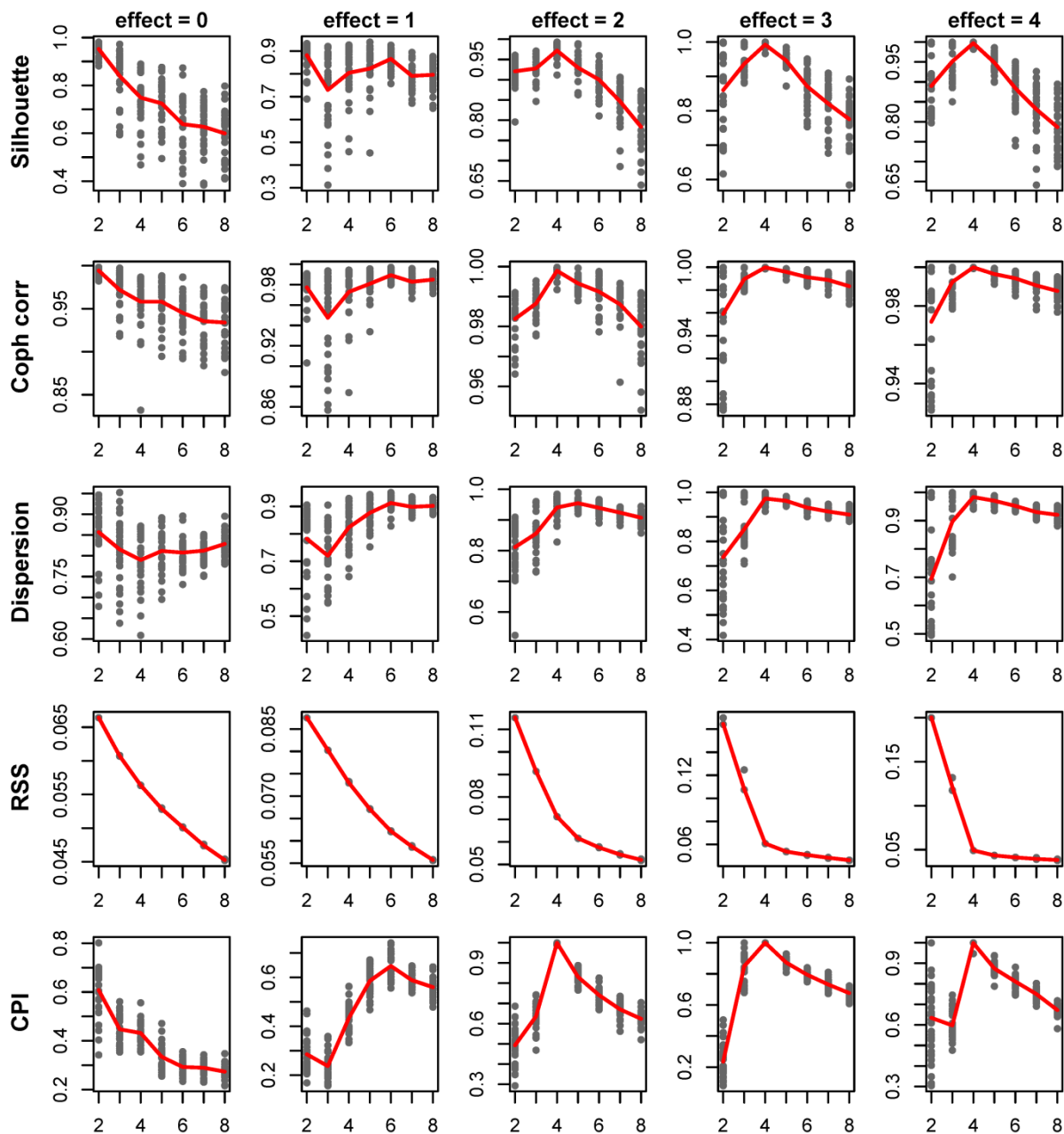

Supplement: S1 Fig — Plots showing the comparison of five different methods of finding optimum number of clusters on the dataset generated using varying effect sizes for true number of clusters k = 4. First row represents silhouette width over k = 2:8 for each of five different scenarios of true clusters 2, 3, 4, 5 and 6 over 30 runs of simulation. The average value of the silhouette widths over 30 runs are overlaid on the plots as line. Cophenetic correlation, Dispersion, Residual Sums of Squares and Cluster Prediction Index are shown on second, third, fourth and fifth rows respectively. (PDF) [file pone.0176278.s003.pdf]

Effect size = 3.5

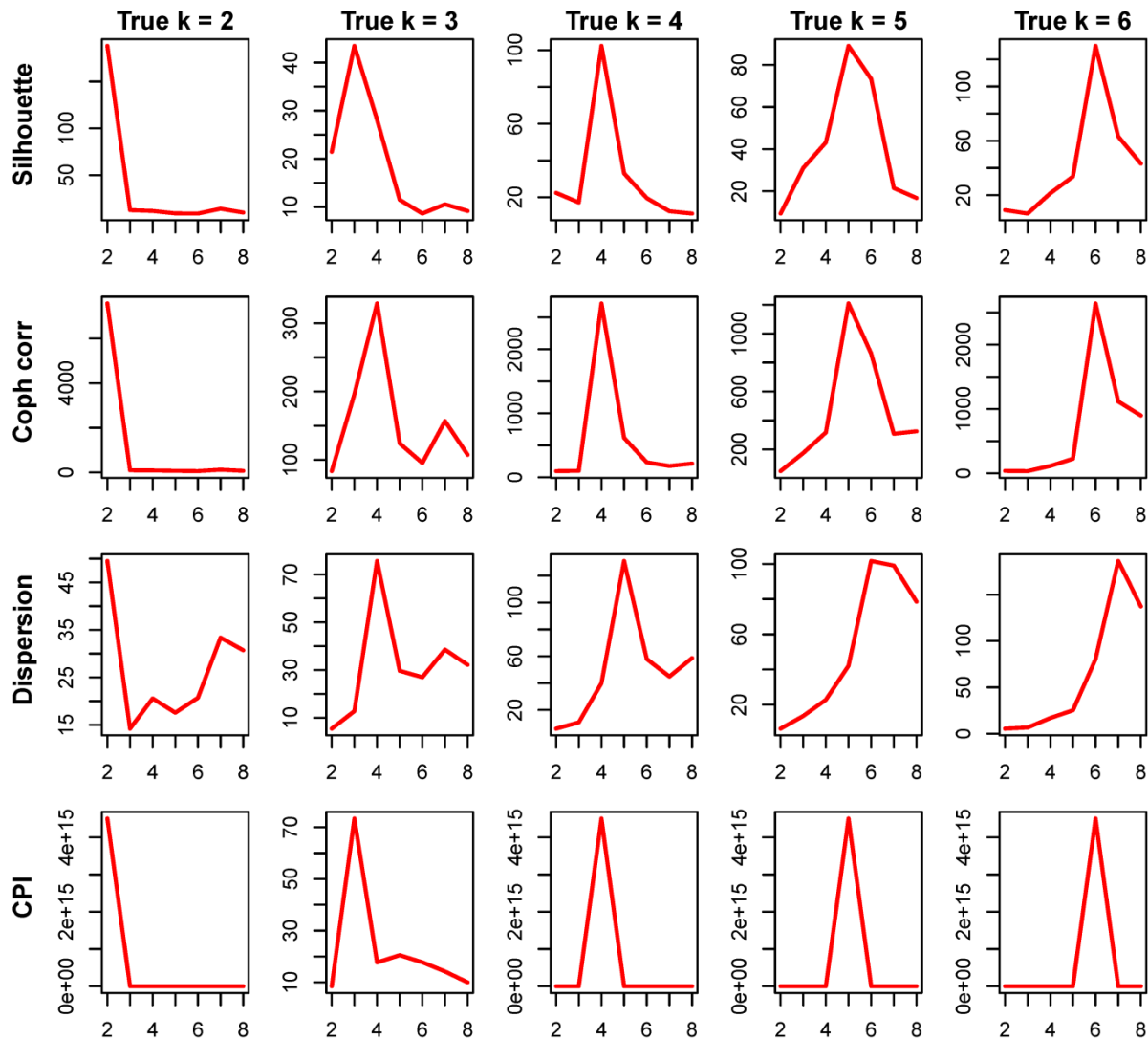

Supplement: S2 Fig — Plots showing the signal to noise ratio (mean/sd) for the four types of measures, Silhouette, Cophenetic correlation, Dispersion and Cluster Prediction Index for finding optimum number of clusters for cluster mean shift effect size of 3.5 and varying scenarios of true number of clusters. Cluster prediction index has the best ability of finding optimum number of clusters with maximum value at the true number of clusters with best precision. (PDF) [file pone.0176278.s004.pdf]

# True number of clusters = 4

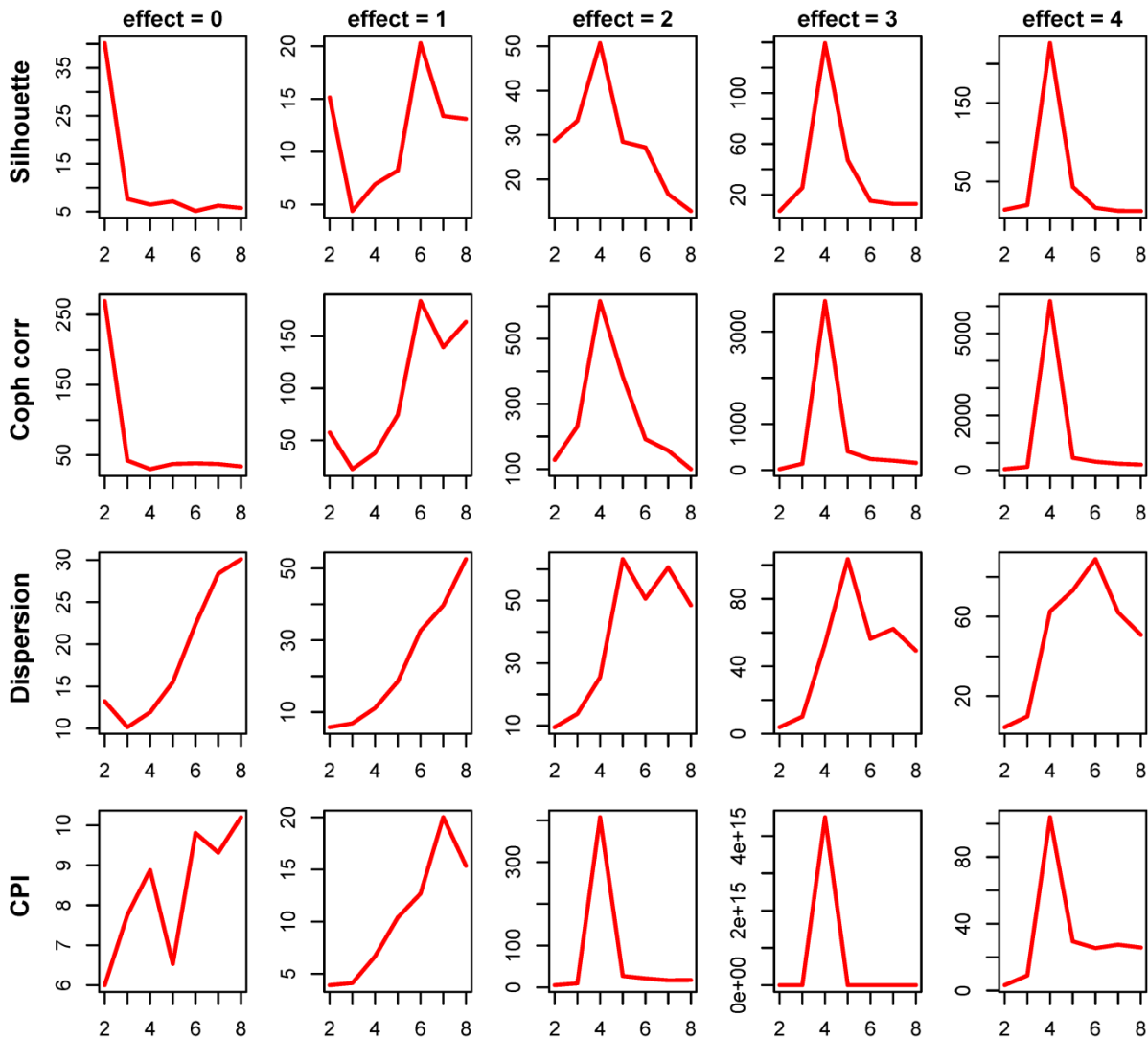

Supplement: S3 Fig — Plots showing the signal to noise ratio (mean/sd) for the four types of measures, silhouette, cophenetic correlation, dispersion and cluster prediction index for finding optimum number of clusters for true number of clusters 4 and varying sizes of cluster mean shift effect. Cluster prediction index has the best ability of finding optimum number of clusters with maximum value at the true number of clusters with best precision if the cluster shift effect size is adequate. (PDF) [file pone.0176278.s005.pdf]

Effect size = 3.5

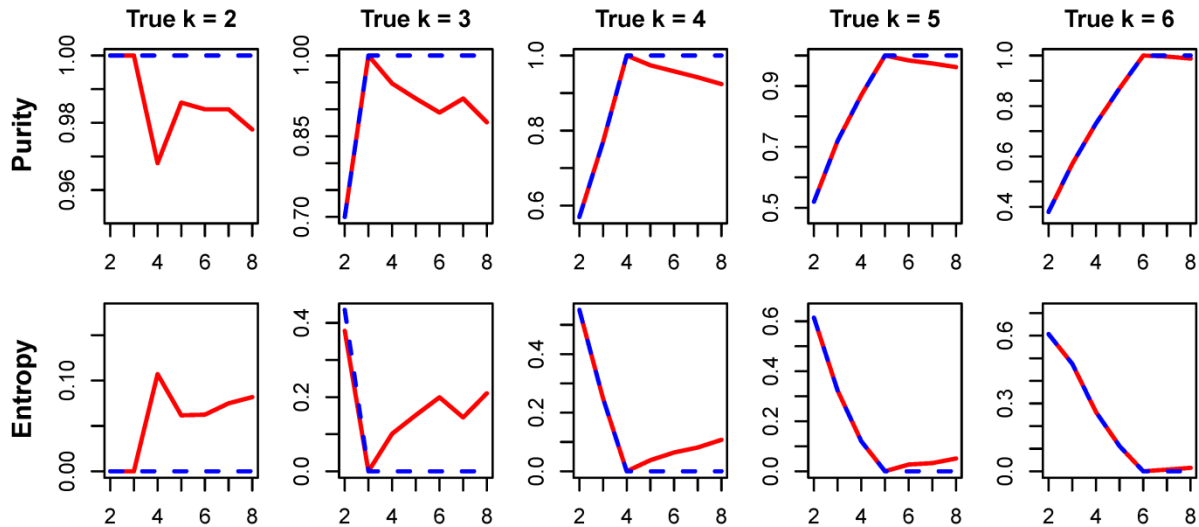

Supplement: S4 Fig — First row represents the plot of purity for intNMF (red) and iCluster (blue) and second row represents plot of entropy for intNMF and iCluster. Purity is expected to result in maximum and entropy is expected to result in minimum at true number of clusters. (PDF) [file pone.0176278.s006.pdf]

# True number of clusters = 4

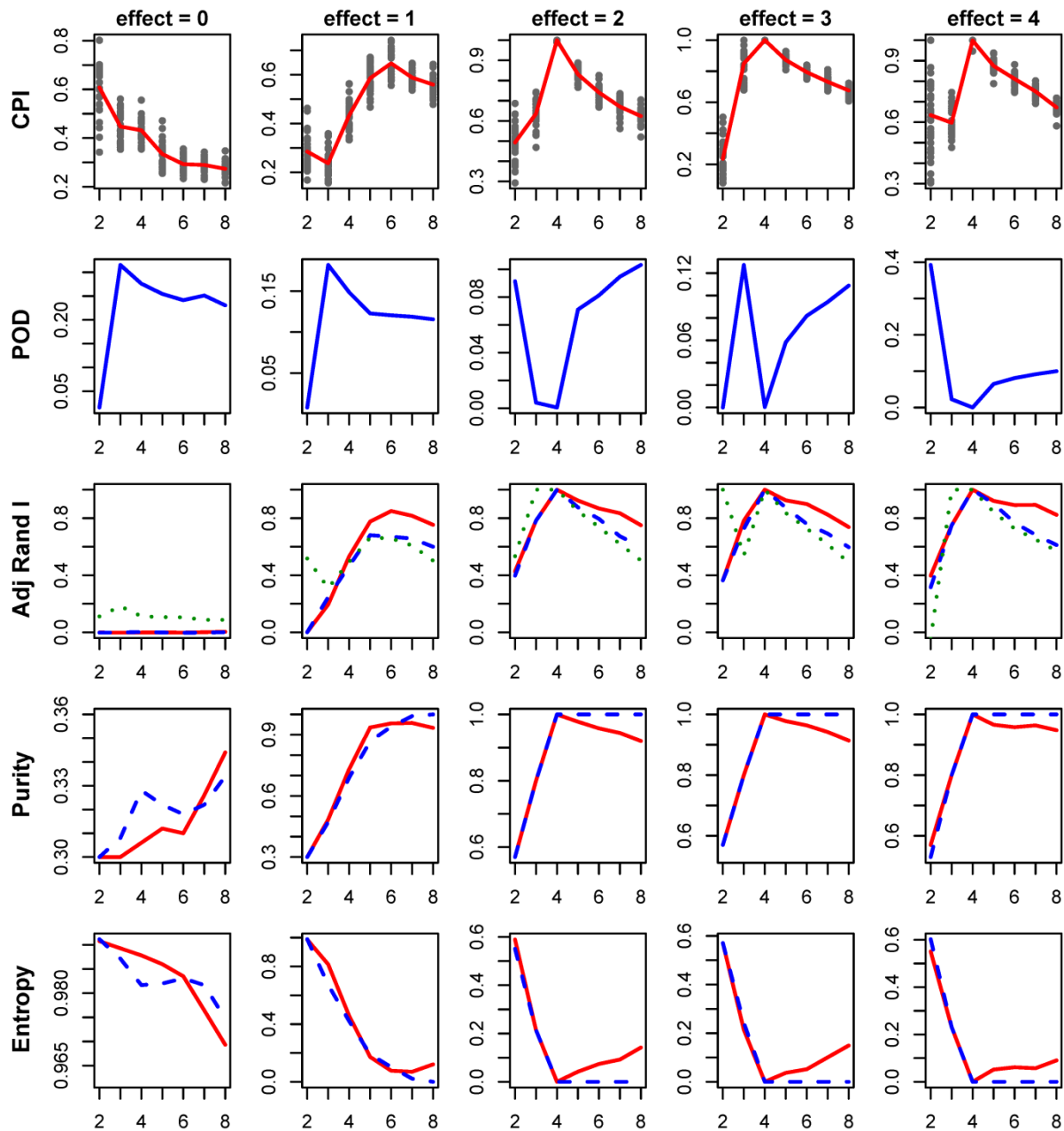

Supplement: S5 Fig — First row represents the cluster prediction index, second row represents the plot of proportion of deviance (POD) given by iCluster method, third row represents adjusted rand index between (i) true and intNMF-clusters (red), (ii) true and iCluster-clusters (blue) and (iii) intNMF-clusters and iCluster-clusters (green), fourth row represents the plot of purity for intNMF and iCluster and fifth row represents plot of entropy for intNMF and iCluster. The POD and entropy are expected to result in minimum at true number of clusters. In other plots, maximum is expected at true number of clusters. (PDF) [file pone.0176278.s007.pdf]

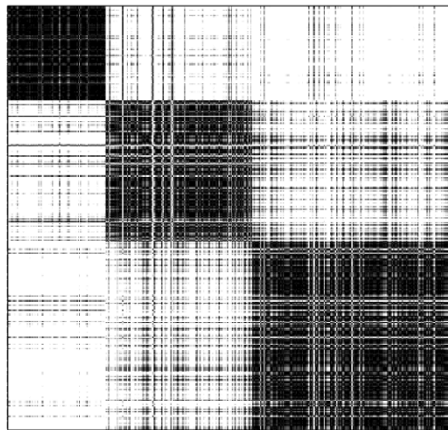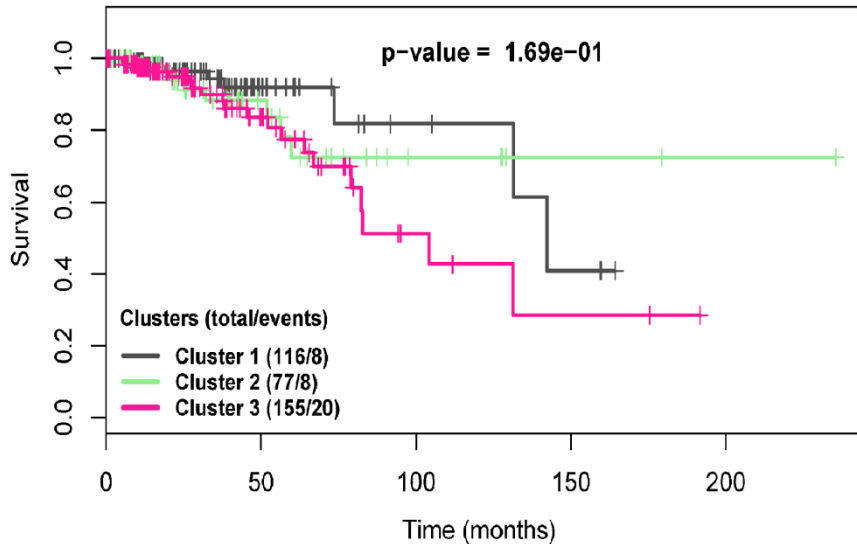

Supplement: S6 Fig — (PDF) [file pone.0176278.s008.pdf]

**(a)**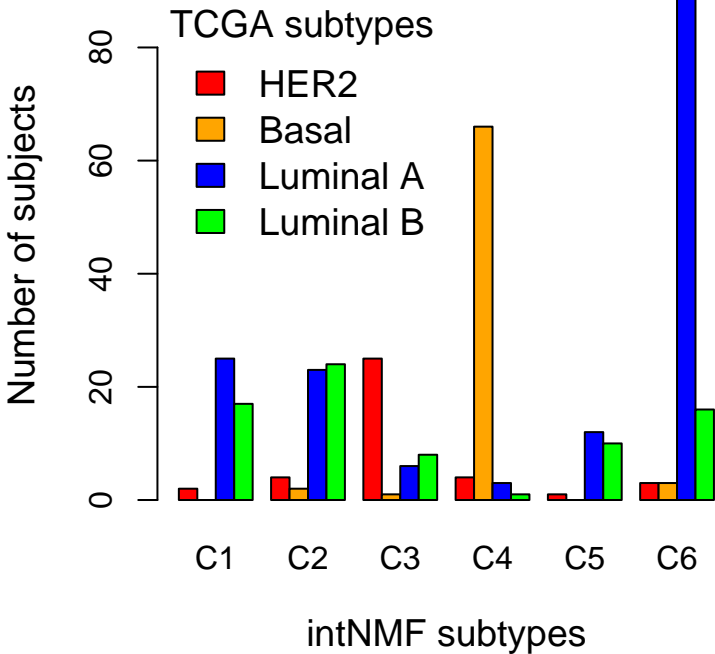**(b)**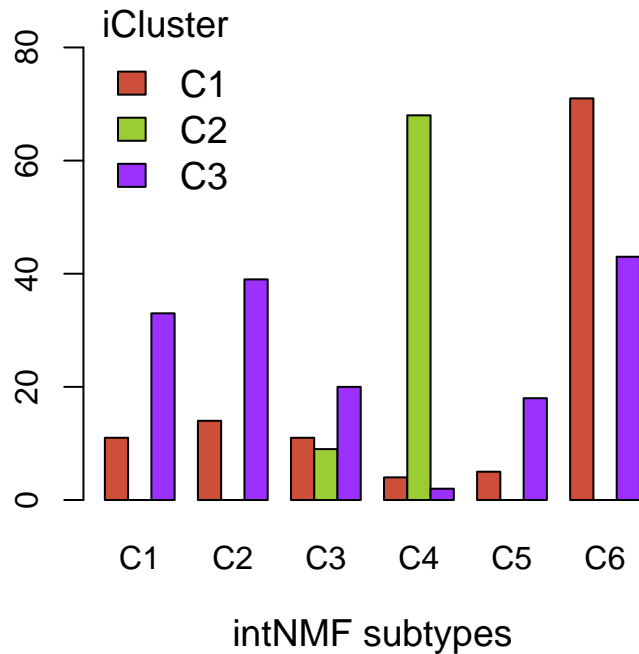

Supplement: S7 Fig — These figures are the graphical representation of Table 1 in the paper. (PDF) [file pone.0176278.s009.pdf]

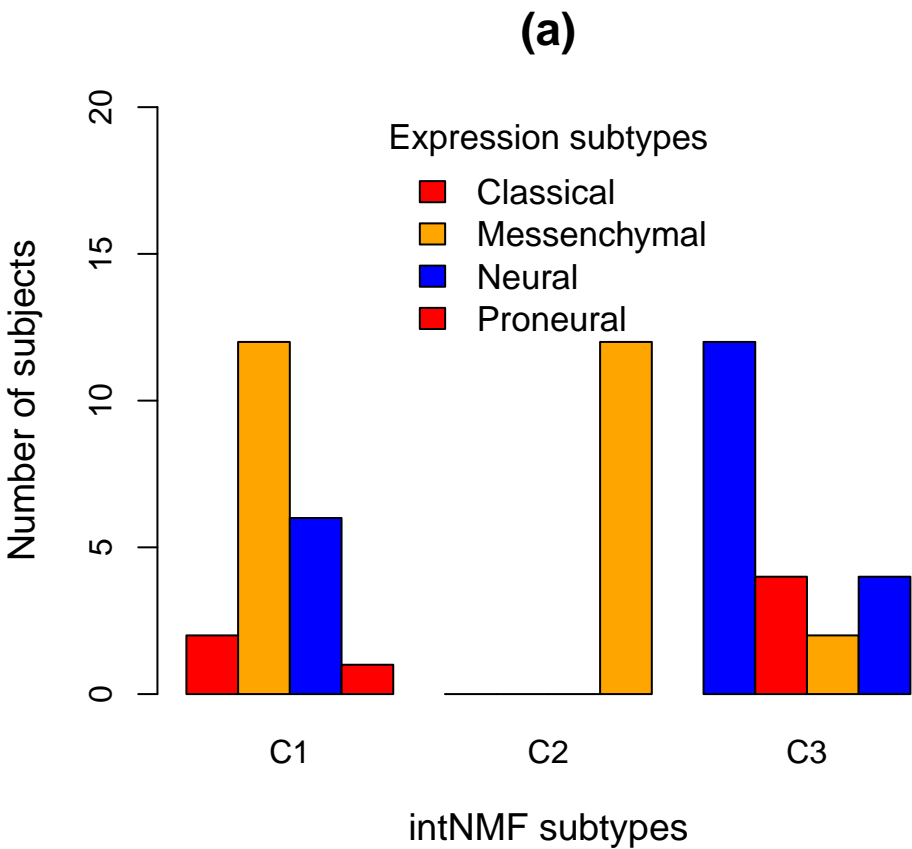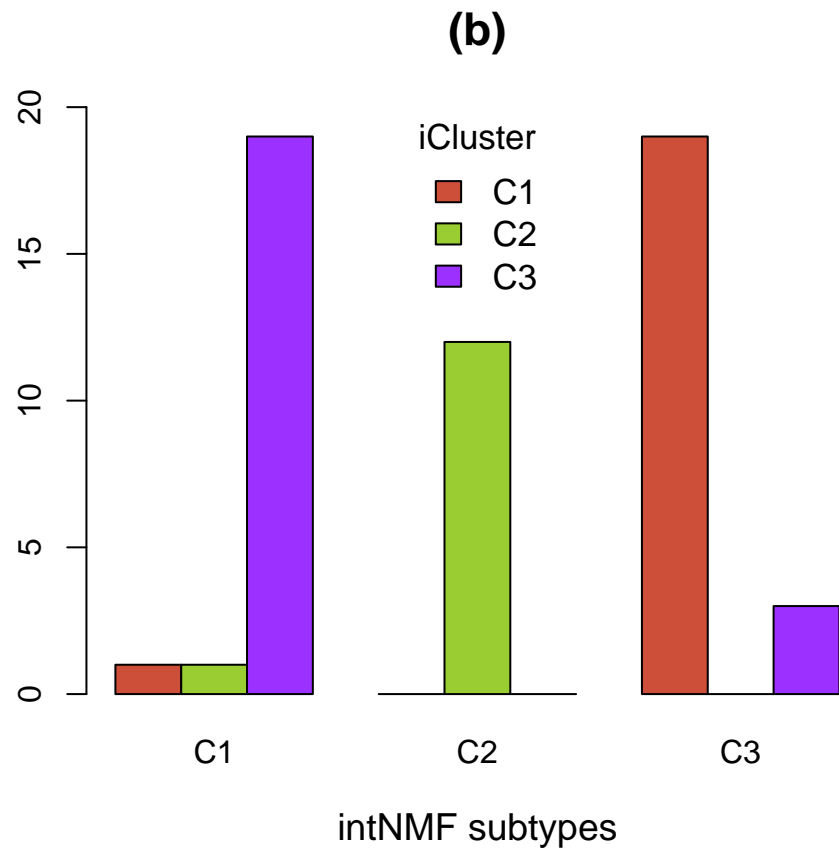

Supplement: S8 Fig — These figures are the graphical representation of Table 2 in the paper. (PDF) [file pone.0176278.s010.pdf]
