# Supplementary material for: Integrative clustering of multi-level ‘omic data based on non-negative matrix factorization algorithm
Source: PLoS One. 2017 May 1;12(5):e0176278. doi: 10.1371/journal.pone.0176278 (PMC5411077; doi:10.1371/journal.pone.0176278)
Supplement: S1 Table — (PDF) [file pone.0176278.s011.pdf]

**TCGA Subtypes**	**iCluster Subtypes**			**Total**
	**C1**	**C2**	**C3**	
**(1) HER2**	14	8	17	**39**
**(2) Basal**	5	65	2	**72**
**(3) Luminal A**	89	4	68	**161**
**(4) Luminal B**	8	0	68	**76**
**Total**	**116**	**77**	**155**	**348**
